# CD147 promotes NSCLC metastasis by inducing secretory autophagy-dependent exosome secretion via TRIM56-mediated ubiquitination and degradation of GCN2

**DOI:** 10.1038/s41418-025-01636-y

**Published:** 2025-12-18

**Authors:** Jie Yang, Chenggong Liao, Xiaohua Liang, Yuan Ke, Ying Sun, Minmin Huang, Meirui Qian, Xu Yang, Hongyong Cui, Huijie Bian, Zhinan Chen, Lingmin Kong

**Affiliations:** 1 Department of Cell Biology, National Translational Science Center for Molecular Medicine, Fourth Military Medical University, Xi’an, China; 2 State Key Laboratory of New Targets Discovery and Drug Development for Major Diseases, Xi’an, China; 3 Department of Oncology, Tangdu Hospital, Fourth Military Medical University, Xi’an, China; 4 Department of Thoracic Surgery, Tangdu Hospital, Fourth Military Medical University, Xi’an, China

**Keywords:** Non-small-cell lung cancer, Autophagy, Ubiquitins

## Abstract

Tumor-derived exosome secretion dynamically correlates with malignant progression, although the mechanisms by which tumor-associated antigens regulate exosome production remain unclear. Here, we found that the number of plasma exosomes increased significantly with the progression of non-small-cell lung cancer (NSCLC) patients and identified that CD147 as a crucial mediator of exosome secretion using mass spectrometry. CD147 exhibited a positive correlation with exosomes release in NSCLC patients and various cell lines and it drove the release of exosome to promote tumor metastasis in vitro and in vivo. Transcriptomic profiling of transgenic CD147 models identified differential gene expression patterns enriched in autophagy-related pathways. Intriguingly, CD147 was found to specifically enhance autophagosome and amphisome biogenesis to promote exosomes release by using transmission electron microscopy, high-sensitivity structured light microscope, RFP-GFP-LC3 adenovirus reporters and immunofluorescence, which indicated the role of CD147 in mediating non-canonical autophagy processes. Mechanistically, CD147 activated the GCN2/EIF2α/ATG12 signaling axis to drive autophagosome assembly but blocked autolysosome maturation by inhibiting VAMP8/STX17/SNAP29-dependent fusion, leading to amphisome accumulation. Proteomics identified TRIM56 as a novel E3 ligase mediating K619 ubiquitination-dependent GCN2 proteasomal degradation. Subsequently, we found that CD147 suppresses TRIM56 expression, thereby stabilizing GCN2 to activate the GCN2/EIF2α/ATG12 axis. Meanwhile, CD147-induced IP3R3-mediated calcium overload facilitated the fusion of autophagosomes with multivesicular bodies to form amphisomes, thus enhancing exosome release. Collectively, our findings reveal a novel mechanism whereby CD147 promotes crinophagy-mediated exosome secretion through dual regulation of GCN2 stability and calcium homeostasis, thereby accelerating NSCLC progression. Our work establishes a new molecular link between autophagy modulation and cancer progression.

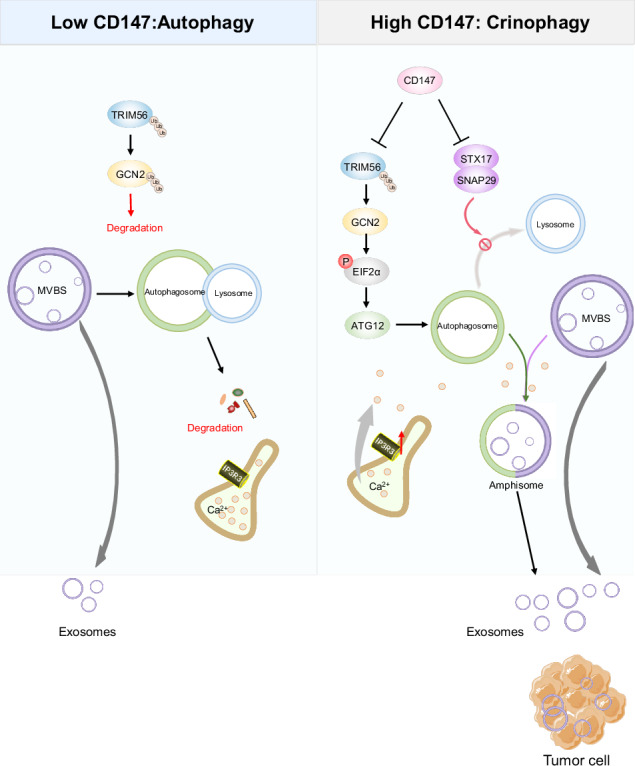

## Introduction

The high metastasis and recurrence of non-small cell lung cancer (NSCLC) contribute to the low 5-year survival rate of patients [1]. Tumor cells can secrete more exosomes, which have been shown to play a crucial role in signal transduction during tumor invasion and metastasis [2–4]. However, the key molecules regulating the release of tumor exosomes and thereby promoting tumor progression remain poorly understood.

The biogenesis of exosomes follows a distinct endosomal pathway: originating from inward budding of endosomal membranes to form multivesicular bodies (MVBs) containing intraluminal vesicles (ILVs). The fate of MVBs dictates exosomal release through plasma membrane fusion (liberating ILVs as exosomes) or intracellular degradation via lysosomal/autophagic pathways [5, 6].

As a principal pathway regulating MVBs turnover, pharmacological or genetic inhibition of autophagic-lysosomal flux has been proposed as a strategy to reduce the degradation of MVBs, thereby potentiating exosome release [7, 8]. However, emerging evidence suggests a new relationship between autophagy activation and exosomal secretion. Since the formal characterization of crinophagy (a non-canonical secretory autophagy pathway) in 2009, scientific attention has increasingly focused on alternative autophagy-mediated secretion mechanisms. The distinctive feature of crinophagy is the formation of amphisomes [9]. Amphisomes are hybrid organelles formed by autophagosome-MVB fusion under pathological or stress conditions. Rab GTPases mediate amphisome fusion with the plasma membrane, releasing exosomes contained within MVBs. This represents a principal mechanism for crinophagy-dependent exosome release [10]. Subsequent studies further established the functional significance of crinophagy-dependent exosomal trafficking in cancer metastasis [11, 12]. Despite these advances, the potential involvement of tumor-associated regulatory factors in modulating crinophagy-mediated exosome release remains poorly understood.

CD147, a tumor-associated type I transmembrane glycoprotein ubiquitously expressed across malignancies [13], has been implicated in diverse oncogenic processes, including enhanced proliferation [14], apoptotic resistance [15], and metabolic reprogramming of lipid/glucose pathways [16, 17]. Recent investigations reveal its novel role in extracellular vesicle biology: CD147 was shown to potentiate small extracellular vesicle (sEV) release in colon cancer stem cells [18]. Thakur et al. further demonstrated hypoxia-induced exosomal hypersecretion in malignant gliomas, correlating with upregulated CD147 and MCT1 expression via label-free quantitative proteomics [19]. Nevertheless, the precise molecular mechanisms underlying CD147-mediated regulation of exosomal secretion remain unclear.

In this study, we identified that CD147 was positively correlated with plasma exosome levels and TNM stages in NSCLC patients. In vitro and in vivo experiments indicated that CD147 induced tumor-derived exosome secretion to promote NSCLC progression. Through integrative multi-omics analysis (mass spectrum and transcriptomic profiling) complemented by functional assays, we systematically elucidated the molecular circuitry that CD147 activated crinophagy via GCN2/EIF2α/ATG12-dependent autophagosome formation, which fused with MVBs in a calcium-dependent manner to form amphisomes. Overall, our findings provide mechanistic insights into CD147-mediated crinophagy activation as a novel regulatory axis controlling tumor exosome release and metastatic progression, potentially identifying new targets for NSCLC treatment.

## Methods and materials

### Data sources

ExoCarta (http://www.exocarta.org/) is a web-based tool for exosome related protein analysis. The human protein atlas (https://www.proteinatlas.org/) is the open access resource for human proteins. TCGA database (https://www.cancer.gov/tcga) is a comprehensive resource library that analyzes patient tumor samples. PhosphoSitePlus (https://www.phosphosite.org) is a database of post-translational modification sites for proteins.

### Patient specimens and information

Blood samples were collected from patients at Tangdu Hospital, Fourth Military Medical University. All participants provided written informed consent prior to specimen acquisition.

Venipuncture-derived whole blood was collected in EDTA-anticoagulated vacutainers and immediately subjected to sequential centrifugation protocols for cellular component separation. Initial processing involved primary fractionation through centrifugation at 1900 × *g* for 10 min at 4 °C to eliminate erythrocytic components, followed by careful transfer of the acellular plasma fraction to sterile polypropylene vessels. Secondary clarification was performed through high-speed centrifugation at 3000 × g under identical thermal conditions to ensure complete removal of residual platelets and cellular debris.

The resultant platelet-poor plasma was aliquoted into cryovials and cryopreserved at −80 °C in ultralow temperature storage systems pending subsequent exosomal isolation procedures. Comprehensive clinicopathological characteristics of the NSCLC cohort, including detailed TNM staging parameters and demographic distributions, are systematically presented in Supplementary Table 1.

### Cell lines and treatment

The human bronchial epithelial cell line BEAS-2B, fibroblast lines HFL1 and MRC-5, along with NSCLC-derived lines H1975, A549, H2170, H460, H661, H226, H358, H292, and murine LLC cells were procured from the National Molecular Medicine Transformation Center Cell Resource Repository. All cell models were authenticated by the suppliers and maintained in RPMI-1640 medium supplemented with 10% FBS and 5% mycillin under standard culture conditions (37 °C, 5% CO₂ humidified atmosphere). Identify the source of cell lines and report if they were recently authenticated.

Isogenic variants including CD147-knockdown H460 (shCD147-H460), CD147-overexpressing A549 (CD147-OE-A549), co-expression of VAMP8/STX17/SNAP29, CD147-overexpressing LLC (CD147-OE-LLC), were generated through lentiviral transduction.

Reverse transfection was performed using lipofectamine 2000 (Invitrogen, California, USA) according to optimized reverse transfection methodology. The lentiviral transfer plasmid pCDH-CMV-MCS-EF1-Puro (System Biosciences) was engineered to express target genes (GCN2, NEDD4L, ITCH, TRIM56, TRIM69) through restriction enzyme-mediated cloning (XbaI/EcoRI). Recombinant plasmids were subsequently transformed into Stbl3 chemically competent E. coli (Thermo Scientific, Massachusetts, USA) for amplification under ampicillin selection (100 μg/mL).

Experimental groups received either chloroquine (CQ, 20 μM) and GM130 (10 μM) through 12-h, Rap (200 nm) and BAF (100 nm) through 24-h continuous exposure in complete medium prior to endpoint analyses. All treatments were conducted under normoxic culture conditions (37 °C, 5% CO₂).

### Isolation and characterization of exosomes

A differential ultracentrifugation protocol was implemented for exosome isolation from conditioned cell culture media and plasma samples.

Following 48-h serum deprivation in exosome-depleted complete medium (prepared through 18-h ultracentrifugation at 120,000 × *g*), cellular supernatants were harvested from subconfluent cultures (70% confluence) and subjected to sequential clarification steps. Initial debris removal was achieved through progressive centrifugation: 3000 × *g* for 10 min (4 °C), 2000 × *g* for 10 min (4 °C), and 10,000 × *g* for 40 min (4 °C). The clarified supernatant was ultracentrifuged in dedicated centrifuge tubes at 100,000 × *g* for 70 min (4 °C). Pelleted vesicles were resuspended in sterile-filtered PBS (pH 7.4) and subjected to wash ultracentrifugation under identical parameters. Purified exosome fractions were quantified via BCA Protein Assay Kit (Beyotime, Shanghai, China) and aliquoted (50–100 μL) in cryoprotective buffer for long-term storage at −80 °C.

A NanoSight analyzer was used to determine the particle sizes of exosomes. Western blotting was performed to determine the expression of exosome marker proteins. An ExoET exosome Quantitation Kit (SBI, California Bay, USA) was used to determine the concentration of exosomes. The quantitative methods for plasma exosomes were based on a study by Wu [20].

### Mice

All animals used in this study were approved by the Animal Care and Welfare Committee of the Fourth Military Medical University. Age-matched male C57BL/6J, Balb/c and athymic nude mice with body mass index maintained at 21 ± 0.5 g was housed under specific pathogen-free conditions. Orthotopic xenograft models were generated via systemic dissemination of CD147-engineered NSCLC or A549 variants through tail vein injection. Following the experimental design, mice were randomly assigned into strain-specific groups with five animals per group (*n* = 5 per group).

### Xenograft tumor model

Female nude mice were used to construct the mouse subcutaneous tumor model. In total, 3 × 10^6^ cells and 100 μg different exosomes in 0.1 mL PBS were subcutaneously inoculated into the posterior flank of nude mice. Following 1 week of subcutaneous injection, the size and weight of the tumor were recorded at 5-day intervals.

About 3 × 10^6^ cells-luciferase were collected in 0.1 mL PBS with or without different exosomes, and then subcutaneously inoculated into the posterior flank of nude mice. When the tumor size reached about 100 mm^3^, the mice were observed by Carestream MISE in vivo imaging system.

### Exosome labeling and tracking in vivo

Exosomes (~5 μg/μL) and 1 mM DIR or DIO (Yeasen, Shanghai, China) were co-incubated at the volume ratio of 200:1 for 20 min and filtered with a 0.22 filter. Approximately 100 μg of DiR-labeled exosomes injected into the mice were incubated in tail veins. Exosome localization in cells and individual organs was observed using Carestream MISE in vivo imaging system.

### Transmission electron microscope

Negative staining transmission electron microscopy (TEM) was performed using optimized sample preparation protocols. Purified exosomes in PBS suspension were loaded onto Formvar-carbon-coated 200 mesh copper grids (Electron Microscopy Sciences) via glass micropipette deposition. Following 5-min, excess fluid was removed using Whatman #1 filter paper in blotting configuration. Grids were subsequently stained with 2% (w/v) phosphotungstic acid for 15 s under controlled humidity (40-50%), followed by air-drying in desiccated chambers. High-resolution imaging was conducted.

The electron microscopy observation of cells was completed by the Electron Microscopy Center at the Fourth Military Medical University.

### Autophagic flow detection

Stable RFP-GFP-LC3 reporter cell lines were established in A549 and H460 NSCLC models through lentiviral transduction (GENECHEM, LV-RFP-GFP-LC3; GC-LV-028). The detailed steps were carried out strictly according to the Virus instructions.

### Intracellular calcium concentration detection

Cytosolic Ca²⁺ levels were measured using Fluo-4 AM calcium probe (Beyotime, Shanghai, China) according to standardized protocols. Cells pretreated with 7 μM Mon (Sigma, Missouri, USA) or 30 μM BAPTA-AM (MCE, New Jersey, USA) for 5 h at 37 °C were loaded with 2 μM Fluo-4 AM in PBS for 30 min. Fluorescence intensity was captured using fluorescence microscope and Flow Cytometry.

### Flow cytometry

Fluorescently labeled LLC cells pre-treated with experimental exosomes were administered via tail vein injection. After 7-day metastatic progression, lungs were perfused with ice-cold PBS and enzymatically dissociated using mixed enzymes (including 5 g/L Collagenase I, 0.15 g/L DNaseI and Hyaluronidase 0.4 g/L) for 40 min at 37 °C. Single-cell suspensions were obtained through 40 μm cell strainer filtration (Falcon), followed by erythrocyte lysis using ACK buffer (Gibco, California, USA). Finally, the sample was resuspended in 1 mL PBS and placed on the machine of flow cytometry.

### Mass spectrometry

Tryptic digests of cellular and exosomal lysates were analyzed on an Orbitrap Fusion Lumos Tribrid mass spectrometer (Thermo Scientific, Massachusetts, USA) operated in data-dependent acquisition mode. SEQUEST algorithm against a nonredundant human protein database (NCBI, Feb 2007) were further used to analyze the resulting tandem mass spectrometry spectra.

### Wound-healing assay

Confluent monolayers of A549 and H460 (6-well plates, Corning, New York, USA) were wounded using 200 μL sterile pipette tips (Eppendorf). After PBS washing, exosome-supplemented serum-free RPMI-1640 was applied. Wound closure kinetics were monitored at 0/24 h using microscope imaging system (Olympus) with 10× phase objective.

### Transwell metastasis assay

24-well transwells (8 mm pore size; Corning, New York, USA) were used to observe the cancer cell invasion ability. A549, H460 and different exosomes were added to the upper chamber, in addition, 10% FBS-containing medium was added to the lower chamber. After 24 h incubation, the cells were fixed and stained with 4% PFA and 0.2% crystal violet respectively. Finally, the chambers were then washed with PBS and counted under a microscope.

### BrdU staining

We performed the BrdU incorporation assay according to the manufacturer’s protocol (Cell Signaling Technology) Briefly, BrdU was diluted to a final concentration of 0.03 mg/mL in fresh RPMI-1640 medium and applied to cells grown on coverslips. Cells were treated with 1.5 M HCl and fixed in 70% ice-cold ethanol for 5 min. After blocking with 3% BSA, samples were immunostained overnight with an anti-BrdU antibody at a 1:1000 dilution. The following day, samples were washed three times with PBS, incubated with a fluorescent secondary antibody for 1 h at room temperature, and washed again three times with PBS. Nucleus were counterstained with DAPI. Representative images were acquired using a fluorescence microscope.

### Co-IP

Protein-protein interactions were investigated using the Pierce™ Co-Immunoprecipitation Kit (Thermo Scientific, Massachusetts, USA) following optimized protocols. Briefly, 20 μg of affinity-purified anti-GCN2 antibody was covalently conjugated to NHS-activated agarose beads through 2-h rotation (25 rpm) at 4 °C in coupling buffer (pH 7.4). Post-conjugation beads were incubated overnight (4 °C/360° rotation) with pre-cleared cell lysates. Bead-bound complexes underwent six sequential washes with ice-cold IP lysis/wash buffer. Elution was performed using low-pH glycine buffer (0.1 M, pH 2.8) followed by immediate neutralization. Eluates were subjected to LC-MS/MS analysis and immunoblot validation.

### Quantitative real-time PCR

Total RNA was isolated using TRI Reagent® (Sigma, Missouri, USA) with phase-separation methodology. Cell cultures (80% confluence) were lysed directly in 6-well plates, followed by chloroform extraction and isopropanol precipitation. RNA integrity was verified. Reverse transcription was performed with PrimeScript™ RT Master Mix (Takara, Kyoto, Japan) under thermal cycling conditions. The detail step was same with the previously described procedure [21].

### Protein preparation and western blot

Cellular/exosomal proteins were extracted using RIPA buffer (25 mM Tris-HCl, 150 mM NaCl, 1% NP-40, 0.5% sodium deoxycholate, 0.1% SDS) supplemented with 1× Halt™ Protease Inhibitor Cocktail (Thermo Scientific, Massachusetts, USA). Protein quantification was performed via BCA assay (Pierce™ 23225) with bovine serum albumin standards. Denatured samples (95 °C/5 min in Laemmli buffer with 5% β-mercaptoethanol) were resolved on Tris-glycine gels (5% stacking/7-15% gradient separation gels) using Mini-PROTEAN Tetra System (Bio-Rad) at 80 V (stacking) → 120 V (separation).

Electrophoretically transferred proteins (PVDF membranes, 0.45 μm; Immobilon®-P) were blocked with 5% non-fat dry milk in TBST (Tris-buffered saline + 0.1% Tween-20) for 2 h. Primary antibodies (Supplementary Table 2) were incubated overnight at 4 °C (1:1000 dilution in blocking buffer), followed by HRP-conjugated secondary antibodies (1:3000; Proteintech, Chicago, USA) at 25 °C/1 h. Chemiluminescent signals were developed using SuperSignal™ West Pico PLUS Substrate (Thermo Scientific, Massachusetts, USA) and quantified via Image Lab 6.1 (Bio-Rad) with background subtraction.

### Immunofluorescence staining

Cells were plated on confocal dishes at 1 × 10^4^cells/cm² density. Following PBS washes (3 × 5 min), samples were fixed in 4% paraformaldehyde for 20 min at 25 °C. Permeabilization was performed using 0.2% Triton X-100 in PBS for 10 min. Non-specific binding was blocked with 5% sheep serum 1 h. Primary antibodies (Supplementary Table 2) were incubated overnight at 4 °C in antibody diluent, followed by Alexa Fluor-conjugated secondaries (1:500; Thermo Scientific, Massachusetts, USA) for 2 h at RT. Nuclear counterstaining utilized DAPI (1 μg/mL; Sigma, Missouri, USA) in Vectashield mounting medium (Vector Labs). Imaging was performed on SIM (High Sensitivity Structured Illumination Microscope).

### Immunohistochemistry (IHC)

Formalin-fixed paraffin-embedded (FFPE) sections were baked at 60 °C for 16 h, deparaffinized in xylene (3 × 20 min), and rehydrated through graded ethanol series (100% → 75%). Antigen retrieval was performed in citrate buffer using a Decloaking Chamber (Biocare Medical) at 95 °C for 20 min. Endogenous peroxidase activity was quenched with 3% H₂O₂ for 15 min. Subsequent immunostaining followed the VECTASTAIN® ABC protocol (Vector Labs) with DAB chromogen development (5 min), counterstained with Mayer’s hematoxylin (Sigma, Missouri, USA). The ImageJ software was used for quantitative analysis of IHC. The average optical density (AOD) was employed to represent the average expression level of the target protein in the tissue sections.

### H & E staining

H & E staining was conducted using the Varistain Gemini ES automated stainer (Thermo Scientific, Massachusetts, USA). Sections were dewaxed as above, stained with Harris hematoxylin (5 min), differentiated in 0.5% HCl/70% ethanol (30 s), and blued in 0.02% ammonia water. Eosin Y (0.5% alcoholic) staining (45 s) was followed by dehydration through ethanol-xylene series. Coverslipping used Permount™ mounting medium (Fisher Chemical).

### Statistics

All quantitative data represent mean ± SEM from three individual biological replicates. Two-group comparisons employed unpaired Student’s *t*-test with Welch’s correction. Multi-group analyses used one-way ANOVA with *Tukey’s* post hoc test (GraphPad Prism 9.4.1). Significance thresholds: *p < 0.05, **p < 0.01, ***p < 0.001.

## Results

### CD147 is involved in the secretion of tumor exosomes

**Fig. 1:**
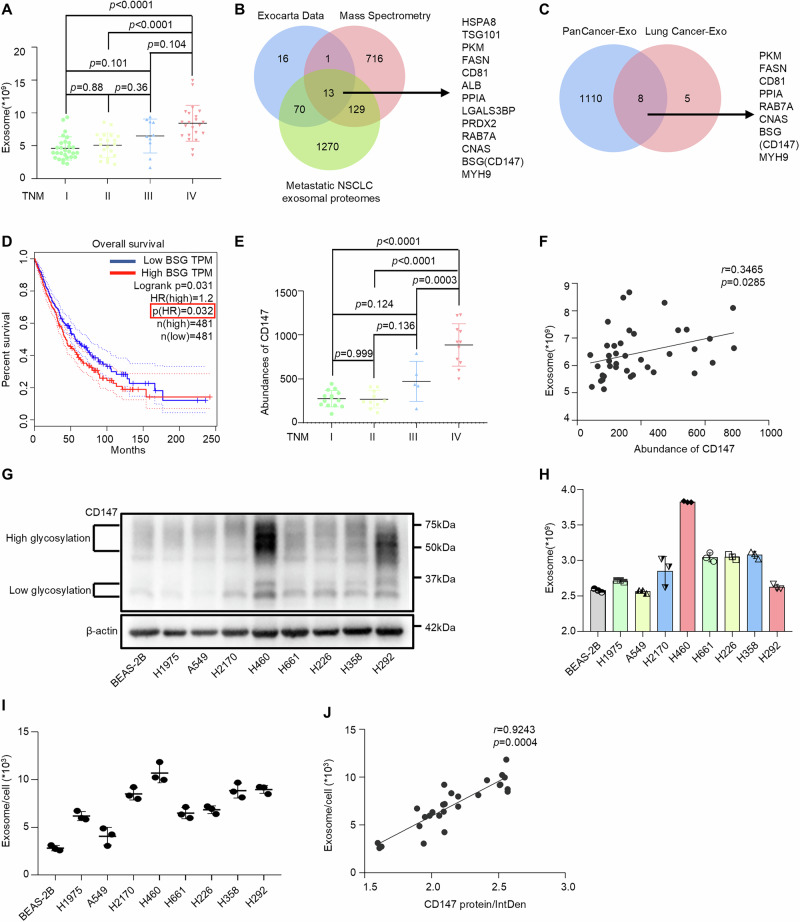
CD147 expression positively correlates with exosome secretion in NSCLC progression. **A** Stage-dependent elevation of circulating exosome levels in NSCLC patients (TNM I-IV, *n* = 40), quantified via ExoCET assay (*****p < 0.001, one-way ANOVA). **B** Comparative mass spectrometry of NSCLC-derived exosomes (current study), published cancer exosomal proteomes, and ExoCarta top 100 exosomal proteins. Venn intersection highlights conserved vesicular cargo. **C** Overlap analysis between pan-cancer exosomal proteome (Extended Data Fig. 1D) and NSCLC-specific signatures (current study). **D** Kaplan-Meier survival curves stratifying NSCLC patients by BSG (CD147) **E** TNM-stage-associated enrichment of exosomal CD147 via quantitative mass spectra. **F** Spearman correlation (*r* = 0.9654, *p* < 0.05) between exosomal CD147 expression and exosome secretion in plasma samples. **G** Basal CD147 expression in NSCLC cells via immunoblotting. **H** Exosome secretion capacity across NSCLC lines normalized to 20 μg exosomal protein. **I** Ultracentrifugation-fractionated exosome yield per 10⁶ cells. **J** Spearman correlation (*r* = 0.9243, *p* < 0.001) between cellular CD147 expression (normalized densitometry) and exosome secretion rate.

To investigate the role of plasma exosomes in NSCLC progression, we collected plasma samples from patients with NSCLC at different tumor node metastasis (TNM) stages (*n* = 40, the information of the NSCLC patients was listed in Supplementary Table 1). Then, plasma-derived exosomes were isolated through standardized ultracentrifugation protocols for quantitative analysis via ExoCET exosome quantification system (vesicles/μg protein). Quantitative analysis revealed a significant increase in plasma exosome levels with the progression of TNM stage, with stage IV patients exhibiting the highest exosome yield, which was statistically significant compared to early-stage cohorts (*p* < 0.05, Fig. 1A). To delineate tumor-associated molecular determinants modulating exosome release, plasma-derived exosomal proteins were isolated and subjected to high-resolution mass spectrometry profiling (The raw data were provided in Supplementary Table 1). A comprehensive proteomic repository was established through integration of three principal data sources: (1) Plasma exosome mass spectrometry data from our institutional cohort; (2) Curated exosome-associated proteins (Top 100) from exosome database (ExoCarta); (3) Metastatic NSCLC exosomal proteomes (liver/brain metastasis subgroups) from established scholarly investigations [22]. Thirteen consensus candidate proteins were identified (Fig. 1B). Furthermore, complementary pan-cancer exosomal proteome mining from ExoCarta was conducted (Extended Data Fig. 1A). A joint analysis of between pancancer-exos and lung cancer-exos was conducted, and eight exosomal proteins were selected for further analysis (Fig. 1C). Kaplan-Meier survival modeling demonstrated significant prognostic stratification wherein elevated exosomal CD147 and PKM concentrations correlated with reduced median overall survival (*p* < 0.05, Fig. 1D and Extended Data Fig. 1B). No significant associations were observed for other candidates (*p* > 0.05, Extended Data Fig. 1B). Quantification of exosomal proteins across TNM staging revealed stage-dependent accumulation of CD147 (Fig. 1E), while PKM levels remained stable across progression states (Extended Data Fig. 1C). Spearman correlation revealed strong positive association between plasma exosomal CD147 expression and exosome secretion levels across TNM staging (Fig. 1F). Comprehensive analysis of the TCGA database reveals a progressive upregulation of CD147 transcript levels corresponding to advancing TNM staging in both lung adenocarcinoma and squamous cell carcinoma (Extended Data Fig. 1D, E). In parallel, proteomic profiling through the CPTAC database demonstrates significantly elevated CD147 protein expression in malignant pulmonary tissues compared to normal controls (Extended Data Fig. 1F, G). Importantly, it has been reported that CD147 expression was associated with clinical stage and distant metastasis status of NSCLC [23].

Given the cellular origin of exosomal CD147 and its significant upregulation in NSCLC cells, we hypothesized CD147 expression may modulates exosome secretion. To analyze the correlation between CD147 expression and exosome release, human pulmonary epithelial cells (BEAS-2B) and various NSCLC lines were cultured to extract exosomes by ultracentrifugation. The typical characteristics of exosomes were selected and presented. Exosomal markers (CD9, CD63, TSG101) were confirmed by immunoblot, with GM130 (a cell-specific marker) absent (Extended Data Fig. 1H). TEM showed characteristic cup-shaped morphology (Extended Data Fig. 1I). Nanosight vesicles tracking analysis revealed 50–150 nm size distribution (Extended Data Fig. 1J). Collectively, these data indicated that the small vesicles extracted were exosomes. Quantitative immunoblotting demonstrated cell line-specific CD147 expression (Fig. 1G). Nanosight vesicles tracking analysis was carried out to quantification quantify of the vesicle yield (Fig. 1H). The number of exosomes per cell type was calculated on the basis on the number of cells (Fig. 1I). Spearman correlation revealed strong positive association between CD147 expression and exosome secretion levels in NSCLC lines (Fig. 1J). In addition to these cells, positive correlation between CD147 expression and exosomes release were also observed in lung fibroblasts (MRC-5 and HFL1) (Extended Data Fig. 1K-M). These findings indicate that CD147 is significantly associated with exosome secretion and may play a role in modulating this process.

### CD147 induces tumor-derived exosome secretion from NSCLC cells

**Fig. 2:**
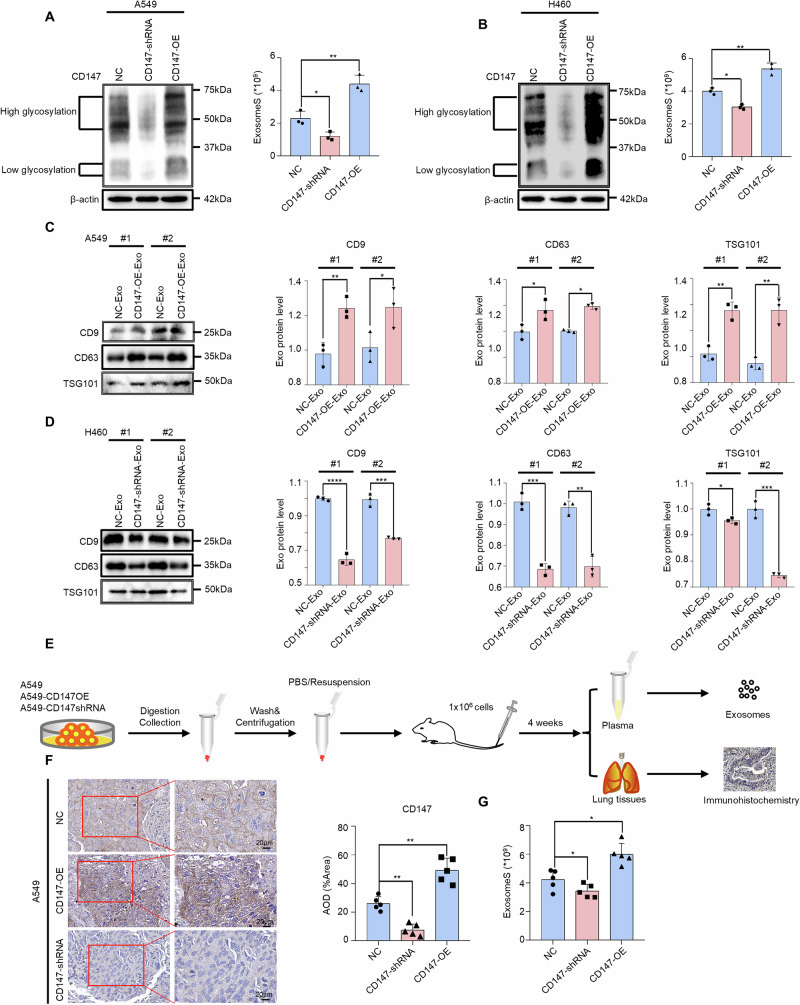
CD147 promotes exosomal secretion in NSCLC cells. **A**, **B** CD147 modulates exosome release in NSCLC cells. Left: Immunoblot analysis of CD147 expression in A549 and H460 cells under genetic perturbations; Right: Dose-dependent correlation between CD147 expression and exosome yield. NC: non-targeting control; shRNA: CD147-knockdown; OE: CD147-overexpression. Exosomal secretion quantified via ultracentrifugation-coupled nanoparticle tracking (Right). **C**, **D** Immunoblot profiles of canonical exosomal markers (CD9, CD63, TSG101) in CD147-engineered A549 and H460 cell lines. **E** In vivo validation of CD147-driven exosomal secretion. Schematic of orthotopic lung cancer model generated via tail vein injection of CD147-modified A549 cells into female nude mice. **F** CD147 overexpression enhances tumor exosome production. Left: Representative IHC staining of CD147 in lung lesions (Scale bar: 100 μm); Right: Immunohistochemical quantitative analysis by using ImageJ. AOD indicates the average optical density of CD147 in the lung tissues. **G** Circulating exosome levels. Plasma exosome concentrations quantified via ExoCET assay (20 μg exosomal protein input, *p < 0.05 vs. NC).

To further clarify the function of CD147 in exosome secretion, A549 and H460 were selected to construct cell lines with CD147 overexpression and knockdown by third-generation lentiviral vectors (pLenti-CMV-T2A-Puro backbone) (Extended Data Fig. 2A, B). ExoCET quantification revealed a dose-dependent relationship between CD147 expression levels and exosomal production (Fig. 2A, B). The level of exosomal protein was considered a criterion for evaluating the relative content of exosomes [24]. As illustrated in Fig. 2C and Fig. 2D, CD147 overexpression significantly enhanced the canonical exosomal biomarkers’ expression (CD9, CD63 and TSG101), whereas the opposite effect was observed in CD147-knockdown cells. Then, we established lung cancer models of mice bearing different CD147 expression to ascertain the role of CD147 in exosome secretion (Fig. 2E). Immunohistochemical quantification confirmed successful CD147 modulation in tumor tissues (Fig. 2F). The number of plasma exosomes was significantly higher in mice bearing CD147-OE cells than in the control group. Conversely, exosomes secreted by CD147-knockdown tumor cells were less abundant than those secreted by control cells (Fig. 2G). These in vivo observations exhibited strong concordance with in vitro data, conclusively demonstrating CD147’s central role in NSCLC-derived exosome secretion.

### Exosomes released driven by CD147 notably promote NSCLC progression in vitro and in vivo

**Fig. 3:**
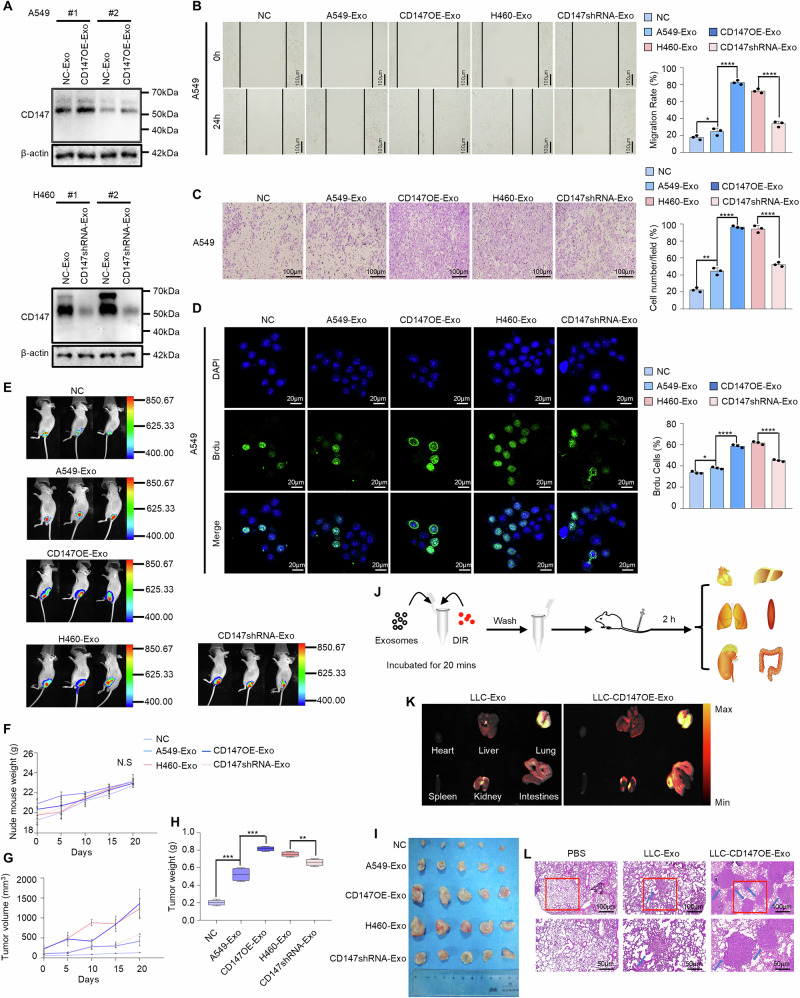
CD147-derived NSCLC exosomes accelerate tumor progression. **A** Immunoblot analysis of CD147 expression in exosomes derived from CD147-engineered (OE) versus CD147-knockdown (shRNA) A549 and H460 cells. Exosomes were isolated via differential ultracentrifugation. **B** Exosomal CD147 enhances migratory capacity. Left: Phase-contrast images of wound closure in A549 monolayers treated with exosomes from indicated groups (0/24 h); Right: Quantification of wound healing rates. A549-Exo indicates the exosomes extract from A549 cell cultural medium; CD147OE-Exo indicates the exosomes extract from CD147 overexpressed A549 cell cultural medium; H460-Exo indicates the exosomes extract from H460 cell cultural medium; CD147shRNA-Exo indicates the exosomes extract from CD147 knockdown H460 cell cultural medium; Other groups have similar meanings. **C** Chemotaxis potentiation by CD147-derived exosomes. Transwell membranes post 24 h migration. Migrated cell counts normalized to control. **D** Exosomal CD147 enhances proliferation capacity. Left: Fluorescence microscope of Brdu in A549 cells treated with exosomes from indicated groups; Right: Quantification of Brdu positive cell proportion. **E** Tumorigenic effects of CD147-derived exosomes. Representative fluorescence images of luciferase signals captured from subcutaneous tumors are shown. A549-Exo indicates subcutaneous tumors formed by injection of A549 cells incubated with exosomes extract from A549 cell cultural medium. Other groups have similar meanings. **F**-**I** Subcutaneous tumor weights, tumor growth curves, and body weight trajectories of nude mice during 4-week exosome administration. **J** Experimental workflow showed the fluorescence tracing of exosomes in vivo. **K** Representative ex *vivo* fluorescent images of various organs in mice injected with DiR-labeled exosomes. **L** Histopathological confirmation. H&E-stained lung sections showing micrometastases (arrows). Scale bars: 100 μm.

To investigate the contribution of CD147-triggered exosomes to metastatic cascades, we performed immunocapture-based quantification of exosomal CD147 across NSCLC cell lines. The results revealed a significant enrichment of CD147 in exosomes from CD147-overexpressing cellular models compared to the control. Conversely, CD147 knockdown resulted in a marked reduction of exosomal CD147 expression (Fig. 3A). Subsequently, we co-cultured exosomes derived from diverse cellular sources with multiple tumor cell lines. Scratch wound healing assays quantified a significant acceleration in gap closure, while transwell migration chambers revealed markedly increased invasive capacity in recipient cells exposed to CD147OE or endogenously high CD147 H460 cells-derived exosomes compared to corresponding controls (Fig. 3B, C and Extended Data Fig. 3A, B). Brdu analysis demonstrated that CD147 overexpression derived exosomes significantly promotes the proliferation of A549 and H460 tumor cells in vitro (Fig. 3D and Extended Data Fig. 3C). Functional validation through co-culture systems demonstrated, exosomes with high CD147 expression significantly enhance the migratory and proliferative capabilities of recipient cells under identical concentrations.

To further elucidate the impact of varying exosome concentrations on tumor progression, exosomes were isolated from equal numbers of A549, A549-CD147OE, H460, and H460-CD147shRNA cells following 48-h culture in exosome-depleted medium. Tumor cells were subsequently incubated with 10 μL of exosomes derived from each of the four cell lines (approximate exosome concentrations: A549: 36 μg; A549-CD147OE: 70 μg; H460: 58 μg; H460-CD147shRNA: 42 μg). Results from wound healing assays, Transwell assays, and Brdu analysis consistently demonstrated that, compared to exosomes derived from A549 cells, exosomes from A549-CD147OE cells significantly augmented tumor cell migration and proliferation. Conversely, exosomes from H460-CD147shRNA cells significantly reversed these pro-tumorigenic phenotypes relative to exosomes from parental H460 cells (Extended Data Fig. 3D–I). Collectively, these findings indicate that the tumor-promoting effects of CD147-overexpression-induced exosomes are attributable to both their elevated exosome concentration and their enriched CD147 cargo in exosome.

To dissect systemic proliferation and metastatic mechanisms, tumor models were established in nude mice, C57BL/6 mice, and Balb/c mice. Nude mice were used to verify the proliferation effects of different exosomes on human lung cancer cells. As shown in Fig. 3F, no significant differences in body weight were observed among the experimental groups. However, lung cancer-derived exosomes markedly increased tumor proliferation compared to PBS control, meanwhile, CD147OE-drived exosome significantly enhanced tumor proliferation compared to the corresponding control group (Fig. 3E, G–I). Subsequently, mouse lewis lung carcinoma line (LLC) was used for further verified in C57BL/6 mice. we tracked the enrichment of exosomes derived from different CD147 expression cells in internal organs and showed that CD147 overexpression promotes the enrichment of exosomes into the lungs and intestines (Fig. 3J, K). Next, metastatic dissemination was quantified through dual-modality analysis (Fig. 3L and Extended Data Fig. 3J, K): Histopathological evaluation (H&E staining) confirmed macro metastatic nodule formation burden in CD147OE-derived exosome cohorts at 4 weeks post-injection; Flow cytometric enumeration of GFP^+^ LLC cells in pulmonary tissues demonstrated 4.9-fold increased metastatic burden in CD147OE-derived exosome cohorts at 1-week post-injection. Additionally, following the tail vein injection of 4T-1 cells resuspended in exosomes or PBS into Balb/c mice, lung metastasis was monitored. It was found that exosomes with high CD147 expression markedly enhanced the pulmonary metastasis of 4T-1 cells (Extended Data Fig. 3L). Taken together, these data demonstrated that CD147-derived exosomes promote NSCLC metastasis and proliferation.

Subsequent analysis of CD147 expression in tumor cells incubated with these distinct exosomes revealed that exosomes exhibiting high CD147 expression significantly upregulated CD147 expression in recipient cells (Extended Data Fig. 3M). Therefore, it is hypothesized that exosomes may contribute to the regulation of tumor cell metastasis and proliferation by augmenting CD147 expression within recipient cells. Elevated CD147 expression has been extensively documented to exert broad pro-tumorigenic effects during cancer progression. Our previous study in hepatocellular carcinoma (HCC) identified that high CD147 expression promotes HCC migration and proliferation via the epithelial-mesenchymal transition (EMT) process [25]. Furthermore, CD147 significantly downregulates E-cadherin expression in T cells, and targeting CD147 has been shown to prevent thymic degeneration by inhibiting EMT in thymic epithelial cells [26]. Subsequently, we examined the expression of EMT marker proteins in lung cancer cell lines with either CD147 overexpression or knockdown. These investigations revealed that CD147 overexpression significantly suppressed E-cadherin expression while markedly increasing Vimentin expression. Conversely, CD147 knockdown significantly enhanced E-cadherin expression and suppressed Vimentin expression (Extended Data Fig. 3N). Further analysis of EMT marker expression in lung cancer cells incubated with the different exosome types demonstrated that CD147-high exosomes significantly decreased E-cadherin expression and significantly increased Vimentin expression in recipient cells (Extended Data Fig. 3O). Collectively, we speculate that tumor-derived exosomes promote lung cancer progression may through the EMT process in recipient cells by modulating CD147.

### CD147 promotes exosomes release through amphisomes formation

**Fig. 4:**
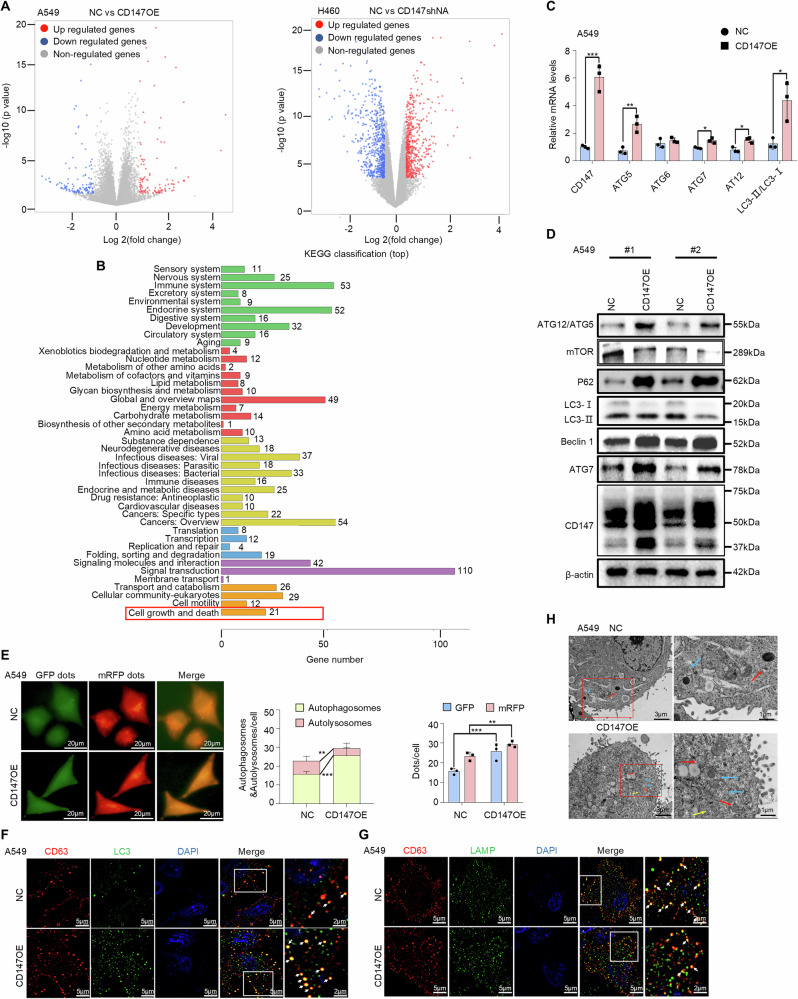
CD147 promotes amphisome formation in NSCLC. **A**, **B** Transcriptomic Profiling of CD147-modulated Pathways. Volcano plot showing differentially expressed genes in CD147-overexpressing (OE) versus knockdown (shRNA) A549/H460 cells; KEGG classification of differential genes. **C** Validation of Autophagic Flux Regulation. qRT-PCR analysis of autophagy-related transcripts normalized to β-actin; **D** Immunoblot of autophagy-related proteins levels. Densitometry quantified versus β-actin. **E** Spatiotemporal Autophagosome-Lysosome Dynamics. Left: Fluorescence microscope of RFP-GFP-LC3 reporter cells. Yellow puncta: autophagosomes (RFP^+^GFP^+^); Red puncta: autolysosomes (RFP^+^GFP^−^); Right: Quantification of autophagic flux index. **F**, **G** Super-resolution Imaging of Organelle Crosstalk. Structured illumination microscopy (SIM) of CD63^+^ MVBs (Alexa Fluor 555, red) co-localizing with LC3^+^ autophagosomes and LAMP^+^ lysosome (Alexa Fluor 488, green). Scale bars: 2 μm. **H** Ultrastructural Evidence of Amphisome Biogenesis. Transmission electron micrographs showing: Red arrows–autophagosomes; Yellow arrows–amphisomes (autophagosome-MVB hybrids); Green arrows–multivesicular bodies (MVBs). Scale bars: 5 μm (overview).

To elucidate the molecular mechanisms underlying CD147-driven exosome release, comparative transcriptomic analyses were conducted in CD147-overexpressing A549 cells (low endogenous CD147 levels) and CD147-knockdown H460 cells (high endogenous CD147 expression) (Fig. 4A and Extended Data Fig. 4A). We found the differentially expressed genes (about 971 both in A549 and H460) were predominantly enriched in biological processes associated with cell growth and death excluding ubiquitous pathways such as signal transduction or cancers overview et al (Fig. 4B). MVBs, serving as precursors to exosomes, undergo one of two distinct intracellular fates: fusion with the plasma membrane for exosomal release or trafficking to the autolysosomal compartment for degradative processing [27]. Emerging evidence has demonstrated that autophagy inhibition could enhance exosomal secretion [7, 28]. Based on this premise, we hypothesized that CD147 might regulate exosome biogenesis through inhibiting autophagy. Contrary to our initial hypothesis, experimental data revealed CD147 enhances autophagic activation through upregulation of core autophagy machinery components including ATG5/ATG12, ATG7, Beclin-1, and LC3Ⅱ/LC3Ⅰ ratios. Notably, paradoxical upregulation of P62 was observed in CD147-overexpressing cells, suggesting impaired autophagic flux despite enhanced autophagic initiation (Fig. 4C, D). To investigate autophagic flux dynamics, we employed RFP-GFP-LC3 lentiviral reporters. This system enables differential visualization of autophagic compartments, with autophagosomes manifesting as yellow puncta (RFP^+^GFP^+^) and autolysosomes as red-exclusive puncta (RFP^+^GFP^-^). Quantitative analysis revealed CD147 overexpression induced significant accumulation of yellow puncta with concomitant reduction of red puncta, demonstrating autophagic flux inhibition (Fig. 4E). LC3 and LAMP were established as canonical markers for autophagosomes and lysosomes, respectively. To elucidate amphisome biogenesis, we examined spatial co-localization patterns between LC3/LAMP and CD63, an established MVB marker. High sensitivity structured illumination microscope (SIM) was implemented for precise co-localization analysis. SIM analysis demonstrated that CD147 overexpression enhanced LC3-CD63 co-localization while diminishing LAMP-CD63 interactions (Fig. 4F, G), which suggests that autophagosomes show greater fusion with MVBs while having reduced interaction with lysosomes when CD147 expression is increased. Ultrastructural analysis via TEM confirmed increased autophagosome and amphisome accumulation in CD147-overexpressing cells (Fig. 4H). Conversely, CD147 knockdown cells exhibited opposite results. Knockdown down of CD147 significantly attenuated the expression of core autophagy regulators, including ATG5-ATG12 complexes, ATG7, Beclin-1, and LC3-II/LC3-I ratios, while simultaneously elevating p62 accumulation (Extended Data Fig. 4B, C). Complementary ultrastructural and functional analyses revealed impaired autophagic flux, decreased in CD63-LAMP colocalization, suppressed autophagosome and amphisome biogenesis (Extended Data Fig. 4D–G). These findings collectively demonstrate CD147 facilitates amphisome formation through coordinated regulation of autophagosome-MVBs interactions.

### High expression of CD147 is necessary for amphisomes formation

**Fig. 5:**
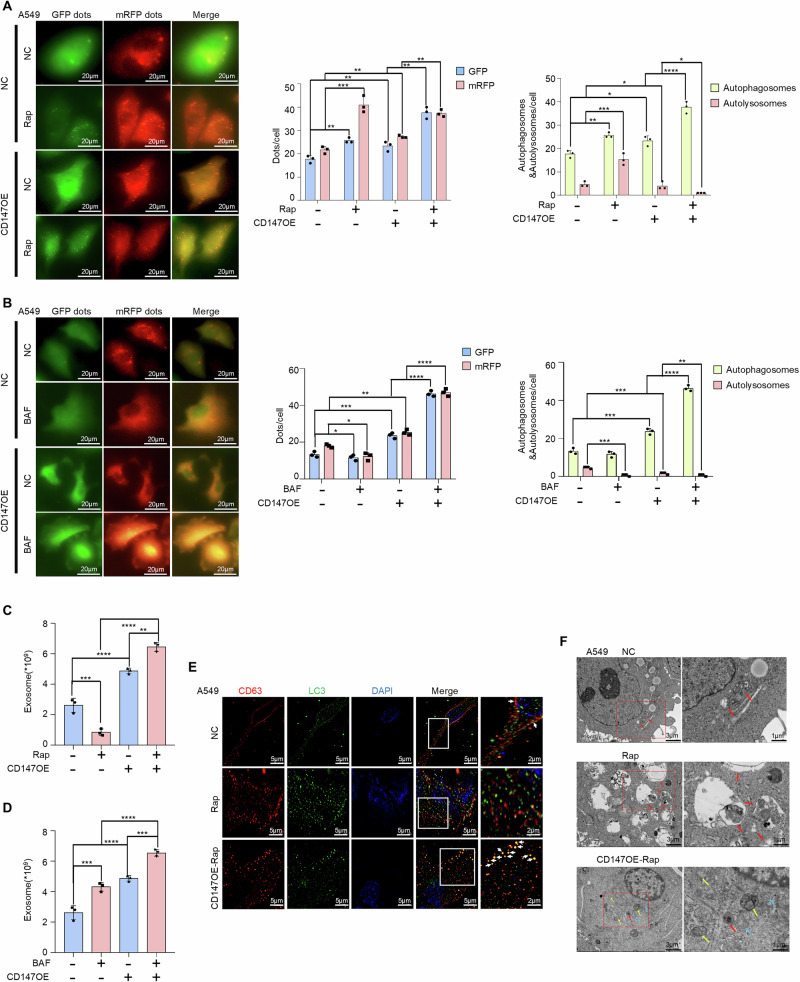
CD147 is essential for amphisomes formation. **A**, **B** Cells were transduced with lentiviral vectors expressing RFP-GFP-LC3 reporters. Representative fluorescence micrographs demonstrate subcellular localization patterns, with quantitative analysis of autophagic puncta: yellow signals (autophagosomes, GFP^+^RFP^+^) and red signals (autolysosomes, GFP^−^/RFP^+^). Data represent mean ± SEM. **C**, **D** Exosome secretion profiles of A549 and H460 cells under CD147 modulation were analyzed using ExoCET exosome quantification assay. Cells were subjected to CD147 overexpression or shRNA-mediated knockdown combined with Rapamycin (200 nM) or Bafilomycin A1 (100 nM) treatment. Exosome concentrations were normalized to 20 μg of exosomal protein. **E** Super-resolution microscopy (SIM) images showing spatial colocalization of CD63 (Alexa Fluor™ 555) and LC3(Alexa Fluor™ 488) in membrane compartments. Scale bars: 5 μm (overview), 2 μm (insets). **F** Ultrastructural evidence of autophagic flux by transmission electron microscopy. Red arrows–autophagosomes; Yellow arrows–amphisomes (autophagosome-MVB hybrids); Green arrows–multivesicular bodies (MVBs). Scale bars: 5 μm (overview).

To elucidate the pivotal role of CD147 in amphisome biogenesis, systematic investigations were conducted utilizing autophagy modulators-Rapamycin (Rap, activator) and Bafilomycin A1 (BAF, inhibitor). Autophagic flux analysis revealed that Rap administration induced complete autophagic progression, marked by significant accumulation of both autophagosomes and autolysosomes. Notably, Rap-mediated autophagosome generation while suppressing autolysosome formation in CD147 overexpression cell line (Fig. 5A). Pharmacological inhibition of autophagy with BAF demonstrated that CD147 overexpression counteracted the suppressive effects of BAF on autophagosome biogenesis (Fig. 5B). CD147 knockdown abolished Rapamycin-induced autophagosome formation and potentiated BAF-mediated autophagic flux inhibition (Extended Data Fig. 5A, B). Exosomal secretion profiling revealed paradoxical regulatory effects: Rap suppressed exosome release in normal autophagy-competent cells despite enhanced autophagosome formation, this suppression was converted to exosomal hypersecretion in CD147-overexpressing models (Fig. 5C). Parallel experiments in H460 cell lines demonstrated CD147 knockdown reversed rapamycin-induced exosomal elevation (Extended Data Fig. 5C). Similar regulatory patterns emerged in BAF-treated cells, where CD147 overexpression exacerbated while CD147 knockdown mitigated BAF-induced exosomal hypersecretion (Fig. 5D and Extended Data Fig. 5D). Observed intercellular variability likely stems from intrinsic CD147 expression heterogeneity. Multimodal imaging validation through immunofluorescence co-localization studies (LC3-CD63) and TEM provided ultrastructural evidence: Rap treatment preserved baseline LC3-CD63 co-localization compare with control group, but significantly amplified colocalization signals in CD147-overexpressing cells (Fig. 5E). Conversely, CD147 knockdown accelerated BAF-induced disruption of LC3-CD63 spatial correlation (Extended Data Fig. 5E). TEM quantification confirmed CD147’s regulatory capacity while Rap increased autophagosome counts without affecting amphisomes in wild-type cells, and induced amphisome biogenesis in CD147 overexpression cell line (Fig. 5F). BAF decreased autophagosome biogenesis and reciprocal effects were observed in BAF-treated CD147 knockdown models (Extended Data Fig. 5F). Collectively, these findings establish CD147 as an essential molecular determinant governing amphisome formation through its regulatory interplay with autophagic machinery.

### Activation of GCN2/EIF2α/ATG12 pathway and inhibition of STX17/SNAP29 are involved in CD147-dependent autophagosome formation

**Fig. 6:**
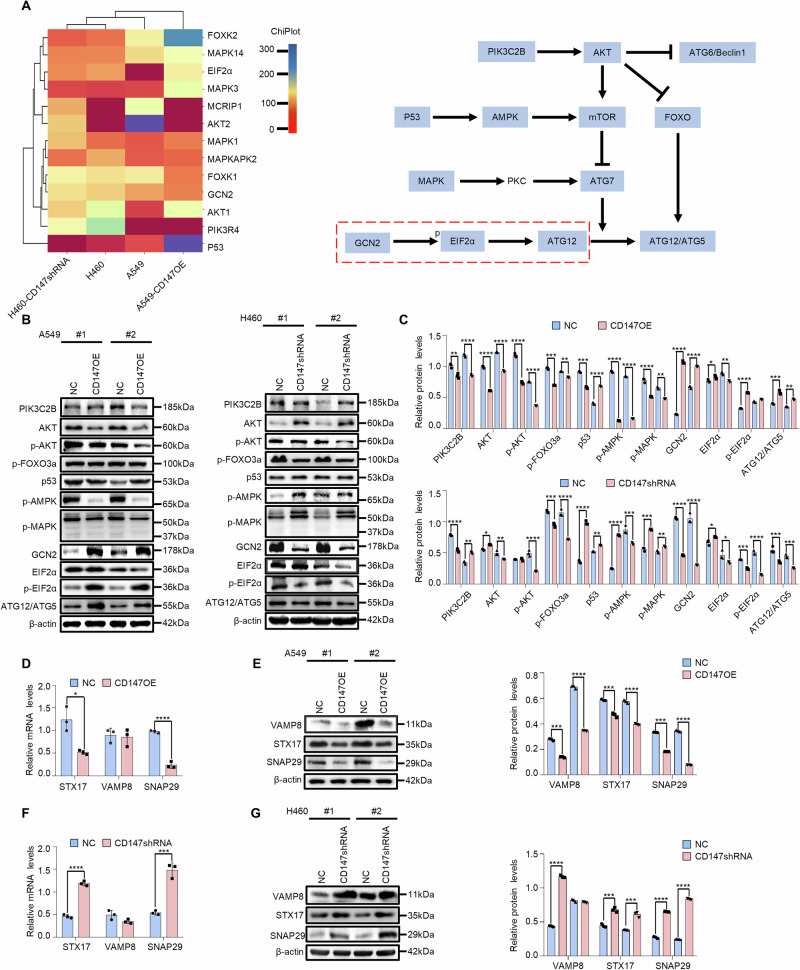
CD147 activates the GCN2/EIF2α/ATG12 signaling and inhibition of VAMP8/STX17/SNAP29 pathway to induce autophagosome formation. **A** Left**:** Mass spectrometry reveals differential expression patterns of autophagy-related pathway through heatmap visualization; right: Systems biology integration of transcriptomic data with canonical pathway analysis identifies potential regulatory networks in autophagy. **B**, **C** Western blot (Left) and quantitative analysis (Right) of autophagy pathway related genes components in A549-NC vs A549-CD147OE and H460-NC vs H460-CD147KD cell models**. D**, **F** qRT-PCR quantification of STX17, VAMP8, and SNAP29 mRNA levels in H460 and A549 cells following CD147 overexpression or knockdown. **E**, **G** Western blot (Left) and quantitative analysis (Right) of STX17, VAMP8, and SNAP29 protein expression in corresponding A549 and H460 cell lines under CD147 manipulation.

To investigate the molecular mechanism underlying CD147-dependent regulation of amphisome biogenesis, we conducted mass spectrometry and performed systematic screening of differentially expressed autophagy-related proteins across cell lines with differential CD147 expression levels. Integrated pathway analysis identified four putative regulatory axes: PI3KC2B/AKT, P53/AMPK, MAPK/ATG7, and GCN2/EIF2α/ATG12 signaling cascades (Fig. 6A). Subsequent validation experiments using western blot analysis demonstrated that CD147 overexpression markedly upregulated protein expression levels of GCN2, p-EIF2α, and the ATG12-ATG5 conjugation complex. Conversely, CD147 suppression substantially diminished phosphorylation of GCN2 and reduced ATG12-ATG5 complex formation (Fig. 6B, C), establishing CD147 as a positive regulator of autophagosome formation through the GCN2/EIF2α/ATG12 signaling axis. Notably, our investigation revealed that CD147 overexpression significantly downregulated STX17 (syntaxin-17) and SNAP29 (synaptosome-associated protein 29) expression (Fig. 6D, E), whereas CD147 knockdown cells exhibited upregulation of these factors (Fig. 6F, G). Given the established role of VAMP8/STX17/SNAP29 complex in mediating autophagosome-lysosome docking, these findings suggest CD147 exerts inhibitory effects on autolysosome formation. Collectively, our data delineate a dual regulatory mechanism whereby CD147 facilitates amphisome maturation by coordinately promoting autophagosome biogenesis through GCN2-mediated signaling while suppressing autolysosome generation via VAMP8/STX17/SNAP29 downregulation.

Furthermore, to explore whether an increased autolysosome maturation induced by VAMP8/STX17/SNAP29 activation could abrogate exosome secretion, we performed rescue experiments targeting VAMP8, STX17, and SNAP29 in CD147-overexpressing cell lines, followed by assessment of exosome secretion. Notably, we found that the activation of VAMP8/STX17/SNAP29 significantly inhibit the CD147-dependent exosome secretion, which indicated that inhibition of autolysosome formation is an important factor for CD147-depedent exosome release (Extended Data Fig. 6A, B).

### Inhibition of GCN2/EIF2α/ATG12 pathway reverses CD147 induced amphisomes formation and exosome release

**Fig. 7:**
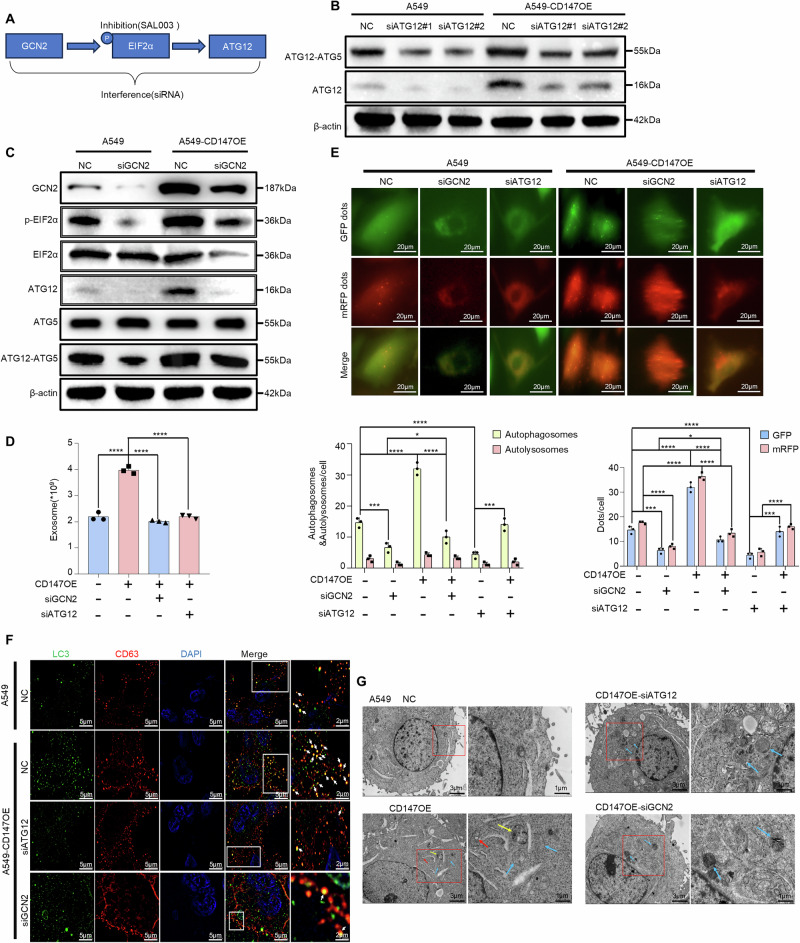
Pharmacological and genetic treatment of the GCN2/EIF2α/ATG12 axis compromises CD147-mediated amphisome formation. **A** Schematic delineation of the GCN2/EIF2α/ATG12 signaling axis. **B**, **C** Western blot analysis of pathway components in A549 cells subjected to siRNA-mediated knockdown of ATG12 (siATG12) or GCN2 (siGCN2) with concomitant CD147 overexpression (CD147OE). **D** Exosomal secretion dynamics quantified by ExoCET assay in A549 cells under combinatorial modulation of CD147 (OE: overexpression) with pathway perturbations (siGCN2/siATG12). Data normalized to 20 μg exosomal protein. **E** Dual-fluorescence microscopy of RFP-GFP-LC3 reporters: Representative images (upper) and quantitation of autophagic compartments (down). Yellow puncta (GFP^+^RFP^+^): autophagosomes; Red puncta (GFP^-^RFP^+^): autolysosomes. **F** Super-resolution microscopy (SIM) images showing spatial colocalization of CD63 (Alexa Fluor™ 555) and LC3(Alexa Fluor™ 488) in membrane compartments. Scale bars: 5 μm (overview), 2 μm (insets). **G** Ultrastructural evidence of autophagic flux by transmission electron microscopy. Red arrows–autophagosomes; Yellow arrows–amphisomes (autophagosome-MVB hybrids); Green arrows–multivesicular bodies (MVBs). Scale bars: 5 μm (overview).

To systematically investigate the mechanistic contributions of the GCN2/EIF2α/ATG12 signaling axis in CD147-mediated amphisome biogenesis and exosomal secretion, we implemented complementary genetic and pharmacological intervention strategies. These included targeted gene silencing (siRNA-GCN2 and siRNA-ATG12) and pharmacological inhibition using the selective EIF2α phosphatase inhibitor SAL003 (Fig. 7A). Immunoblotting analyses demonstrated that CD147 overexpression-induced upregulation of GCN2 and ATG12/ATG5 complexes was effectively abrogated by their respective siRNA-mediated knockdown (Fig. 7B, C). Notably, siRNA-GCN2 administration substantially attenuated CD147-driven elevations in ATG12, ATG12/ATG5 conjugation, and p-EIF2α levels, while maintaining basal ATG5 and total EIF2α expression unaffected (Fig. 7C). Quantitative assessments confirmed significant reductions in GCN2 and ATG12 protein levels in H460 cells following their respective siRNA treatments (Extended Data Fig. 7A, B). Pharmacological modulation via SAL003 successfully restored p-EIF2α expression diminished by CD147 depletion (Extended Data Fig. 7C). Exosomal quantification using the ExoCET assay revealed that genetic suppression of GCN2 or ATG12 significantly attenuated CD147-overexpression-enhanced exosome secretion (Fig. 7D). Parallel experiments in H460 cells demonstrated similar suppression of exosomal release following GCN2/ATG12 knockdown, though SAL003 failed to rescue exosome production in CD147-deficient cells (Extended Data Fig. 7D). Autophagic flux monitoring through dual fluorescent labeling showed marked reduction in CD147-induced autophagosome accumulation (yellow puncta) following pathway inhibition, with consistent observations across both A549 and H460 cellular models (Fig. 7E and Extended Data Fig. 7E). Intriguingly, SAL003 treatment enhanced autophagosome formation in wild-type H460 cells while promoting autolysosomal maturation (red puncta) in CD147-depleted counterparts (Extended Data Fig. 7E). Co-localization studies and TEM provided ultrastructural validation. Both A549 and H460 systems exhibited significant reduction in LC3-CD63 colocalization following GCN2/ATG12 silencing, with SAL003 demonstrating limited efficacy in restoring amphisome formation in CD147-knockdown models (Fig. 7F and Extended Data Fig. 7F). Ultrastructural analyses confirmed decreased autophagosome-amphisome populations in genetically modified cell lines, while p-EIF2α activation via SAL003 preferentially enhanced autophagosome biogenesis without promoting amphisome biosynthesis in CD147-low-expressing cells (Fig. 7G and Extended Data Fig. 7G). Collectively, these multimodal investigations establish that pharmacological or genetic disruption of the GCN2/EIF2α/ATG12 axis effectively impedes CD147-dependent amphisome assembly. Crucially, our findings demonstrate CD147 as an essential molecular coordinator required for GCN2/EIF2α/ATG12-mediated amphisome generation.

### CD147 prevents proteasomal degradation of GCN2 by inhibiting the E3 ubiquitin ligases (TRIM56) expression

**Fig. 8:**
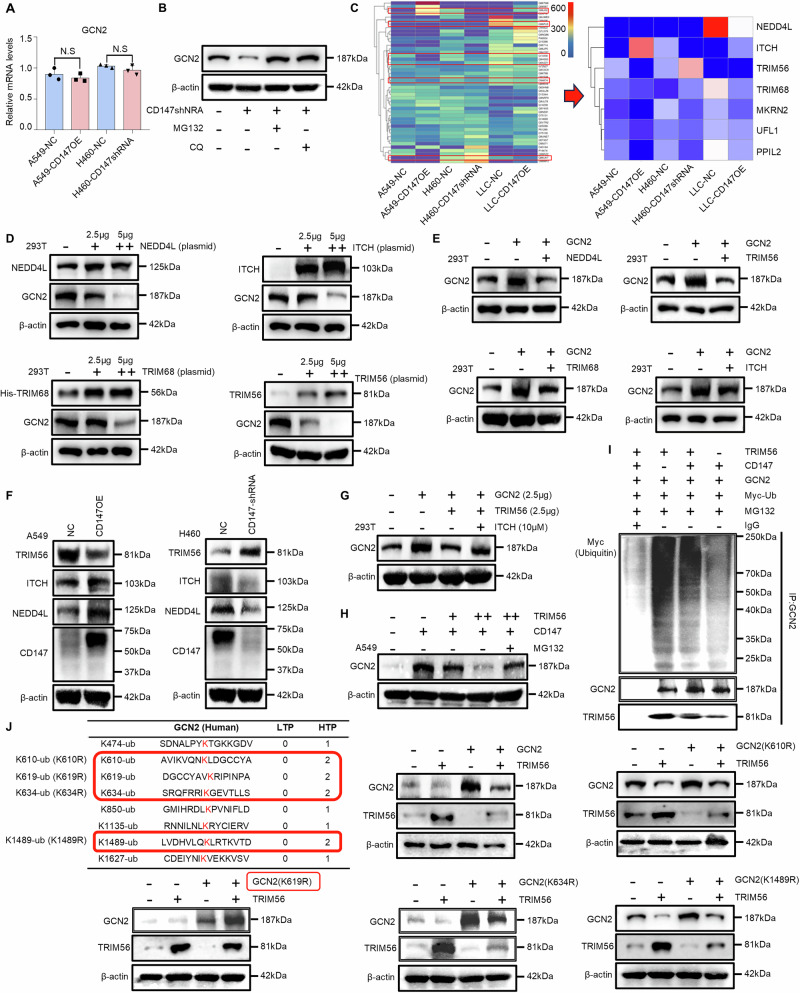
CD147 stabilizes GCN2 through TRIM56-mediated ubiquitin-proteasomal regulation. **A** qRT-PCR quantification of GCN2 mRNA levels in H460 and A549 cells following CD147 overexpression or knockdown. **B** Immunoblot profiling and densitometric analysis of GCN2 protein dynamics in H460 parental vs. CD147-depleted (shRNA) cells treated with lysosomal inhibitor chloroquine (CQ, 20 μM) or proteasomal inhibitor MG132 (10 μM). **C** Heatmap analysis reveals differential expression profiles of E3 ubiquitin ligases across A549, H460, and LLC cell lines under CD147 genetic manipulation (OE: overexpression; shRNA: knockdown), as determined by quantitative mass spectrometry. **D** Systematic screening of candidate E3 ligases (NEDD4L, ITCH, TRIM56, TRIM68) through plasmid titration in HEK293T cells, with 2.5 μg transfection dose established for functional studies. **E** Competitive regulation assay: Immunoblot detection of GCN2 expression in HEK293T cells co-transfected with fixed-dose GCN2 (2.5 μg) and NEDD4L, ITCH, TRIM56, TRIM68 expression plasmids. **F** E3 ligase expression patterns in CD147-manipulated A549 and H460 models assessed by immunoblotting. **G**, **H** Pharmacological rescue experiments: Immunoblot analysis of GCN2 stability in HEK293T and A549-CD147OE systems treated with proteasomal inhibitor MG132 (10 μM) under TRIM56 overexpression. **I** Co-IP assays using anti-GCN2 antibody followed by immunoblot detection of ubiquitination states under combinatorial modulation of CD147, TRIM56, and MG132 treatment. **J** Site-directed mutagenesis screen: Immunoblot validation of TRIM56-mediated ubiquitination using GCN2 mutants with lysine residue substitutions (K610R, K619R, K634R, K1489R).

To elucidate the molecular mechanisms underlying CD147-mediated regulation of the GCN2/EIF2α/ATG12 signaling axis, we performed comparative analysis of GCN2 transcriptional expression in stable CD147-overexpressing and knockdown cellular models. Quantitative RT-PCR analysis revealed no statistically significant alterations in GCN2 mRNA levels across experimental groups (Fig. 8A). Subsequently, CD147-deficient H460 cellular models were subjected to pharmacological intervention using CQ (a canonical autophagy inhibitor) and MG132 (a proteasome-specific inhibitor). Western blotting revealed that both pharmacological agents effectively reversed the GCN2 expression downregulation resulting from CD147 knockdown (Fig. 8B). Complementary evidence from Fig. 6 demonstrates CD147’s capacity to suppress autophagosome biogenesis through decreasing the expression of STX17 and SNAP29. This regulatory mechanism provides a plausible explanation for CQ-mediated mitigation of GCN2 depletion in CD147-compromised cells. Thus, our study identifies the characterization of CD147-driven ubiquitination processes that regulate GCN2 proteasomal degradation. Through comprehensive multicellular proteomic profiling analysis, seven E3 ligases were identified as potential regulators of GCN2 protein degradation via the ubiquitin-proteasome pathway (Fig. 8C). Based on the consistency of their mass spectrometry detection across multiple cell lines, NEDD4L, ITCH, TRIM56, and TRIM68 were selected for subsequent investigation (Extended Data Fig. 8A). Expression vectors encoding GCN2 and these four E3 ligases were successfully constructed. Transfection optimization in 293 T cells demonstrated that 2.5 μg plasmid concentration effectively achieved protein overexpression, establishing this parameter as our standardized transfection condition (Fig. 8D and Extended Data Fig. 8B). Additionally, the expression of GCN2 in 293 T cells exhibited a dose-dependent attenuation correlating with escalating transfection concentrations of NEDD4L and TRIM56, while ITCH and TRIM68 demonstrated no significant alteration in GCN2 expression at a transfection concentration of 2.5 μg. Meanwhile, the expression of GCN2 is significantly decreased when the concentration of transfection is 5 μg for ITCH and TRIM68 (Fig. 8D). Furthermore, co-transfection experiments in 293 T cells revealed that NEDD4L and TRIM56 significantly enhanced GCN2 degradation (Fig. 8E). This identifies NEDD4L and TRIM56 as E3 ligases for GCN2. Subsequent evaluation in CD147-modulated cell models demonstrated that CD147 overexpression substantially suppressed TRIM56 expression, while CD147 knockdown conversely upregulated TRIM56. Other E3 ligases exhibited inverse regulatory patterns, suggesting CD147-mediated GCN2 stabilization occurs primarily through TRIM56 suppression (Fig. 8F). MG132 was employed to further confirm the function of TRIM56. MG132 treatment markedly inhibited TRIM56-mediated GCN2 degradation, confirming ubiquitin-proteasome pathway involvement (Fig. 8G). Rescue experiments in CD147-overexpressing cells demonstrated dose-dependent restoration of GCN2 degradation by TRIM56 transfection, which was again blocked by MG132 co-treatment (Fig. 8H). Moreover, Co-immunoprecipitation assays showed that TRIM56 could interact with GCN2 in HEK293T cells. TRIM56 overexpression significantly enhanced GCN2 ubiquitination whereas the overexpression of CD147 remarkably reduced GCN2 ubiquitination in the presence of MG132 for 12 h (Fig. 8I). These results delineate a tripartite regulatory axis wherein TRIM56-mediated ubiquitination of GCN2 is counterbalanced by CD147’s deubiquitination-like activity, suggesting competitive modulation of the integrated stress response pathway. Collectively, our findings established that CD147 attenuates ubiquitin-proteasome-mediated degradation of GCN2 by suppressing TRIM56 expression. This regulatory axis ultimately activates the GCN2/eIF2α/ATG12 signaling pathway.

TRIM56 is identified a new E3 ubiquitin ligase for GCN2. To delineate the ubiquitination sites through which TRIM56 exerts regulatory effects on GCN2, we performed comprehensive ubiquitination site prediction for GCN2 using the *PhosphoSitePlus* website. Based on the prediction outcomes and cross-species sequence conservation analysis, four candidate lysine residues (K610, K619, K634, and K1489) were selected for systematic investigation (Extended Data Fig. 8C, D). Site-directed mutagenesis was conducted to substitute these lysine residues with arginine (K → R), thereby generating a series of GCN2 mutant constructs. Western blot analysis revealed significant upregulation of GCN2 protein expression when transfected at 2.5 μg plasmid concentration across all constructs. Subsequent co-transfection experiments in 293T cells demonstrated that while TRIM56 effectively promoted proteasomal degradation of wild-type GCN2 in accordance with previous observations, this degradation was specifically abolished when TRIM56 was co-expressed with the K619R mutant variant. Notably, the other three mutants (K610R, K634R, and K1489R) maintained normal degradation responsiveness to TRIM56 regulation (Fig. 8J). These findings collectively suggest that 619 K serves as the critical ubiquitination site mediating TRIM56-dependent degradation of GCN2.

### Activation of TRIM56 inhibits CD147 induced amphisomes formation and exosome release

**Fig. 9:**
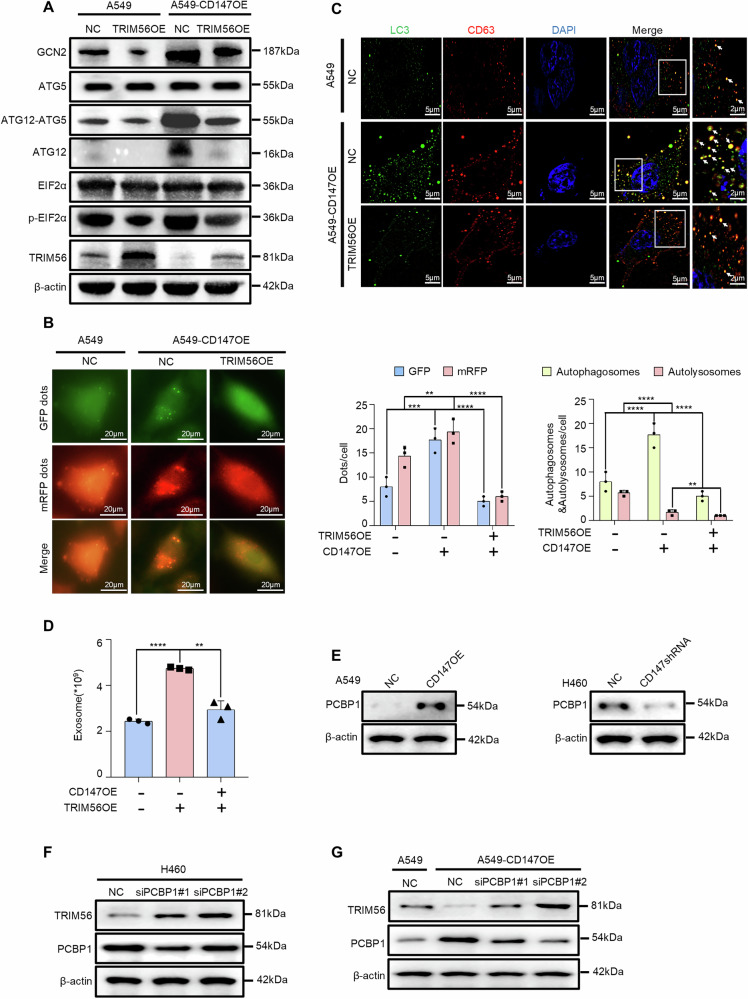
TRIM56 suppresses CD147-mediated activation of the GCN2/EIF2α/ATG12 axis and exosome release. **A** Immunoblot analysis of GCN2/EIF2α/ATG12 signaling components in A549 parental vs. CD147-overexpressing (CD147OE) cells following TRIM56 overexpression. **B** Autophagic flux quantification by RFP-GFP-LC3 reporters in in A549 parental vs. CD147-overexpressing (CD147OE) cells following TRIM56 overexpression: Representative fluorescence microscope (left) and quantitative analysis of autophagic puncta (right). Yellow signals (GFP^+^RFP^+^) denote autophagosomes; Red signals (GFP^-^RFP^+^) indicate acidified autolysosomes. **C** Super-resolution microscopy (SIM) images showing spatial colocalization of CD63 (Alexa Fluor™ 555) and LC3(Alexa Fluor™ 488) in membrane compartments. Scale bars: 5 μm (overview), 2 μm (insets). **D** Exosomal secretion profiles assessed by ExoCET assay in CD147-manipulated models (OE: overexpression; shRNA: knockdown) with concomitant TRIM56 modulation. Secretion levels normalized to 20 μg exosomal protein. **E** The PCBP1 expression was analyzed via immunoblotting in A549/H460 cells treated with CD147 overexpression or knockdown. **F** Immunoblot analysis of TRIM56 and PCBP1 in H460 parental vs. H460-siPCBP1 cells. **G** Immunoblot analysis of TRIM56 and PCBP1 in A549 parental vs. CD147-overexpressing (CD147OE) cells following PCBP1 knockdown.

To elucidate the role of TRIM56 in GCN2/EIF2α/ATG12 signaling pathway activation and autophagic vesicle formation induced by CD147, we employed transgenic technology to modulate TRIM56 expression in CD147-knockdown and CD147-overexpressing cell lines, respectively. Western blot analysis revealed that TRIM56 overexpression significantly reversed the TRIM56 reduction caused by CD147 overexpression. Furthermore, TRIM56 overexpression effectively suppressed GCN2/EIF2α/ATG12 signaling pathway activation induced by CD147 overexpression (Fig. 9A). Autophagic flux monitoring demonstrated that TRIM56 overexpression markedly inhibited the CD147 overexpression-induced increase in autophagosomes (yellow puncta) (Fig. 9B). Conversely, TRIM56 interference substantially reversed both the TRIM56 elevation and GCN2/EIF2α/ATG12 signaling inhibition resulting from CD147 knockdown (Extended Data Fig. 9A). Correspondingly, autophagic flux analysis showed that TRIM56 knockdown significantly augmented the CD147 knockdown-mediated reduction of autophagosomes (yellow puncta) (Extended Data Fig. 9B). Immunofluorescence staining confirmed that TRIM56 overexpression significantly attenuated the enhanced CD63-LC3 co-localization induced by CD147 overexpression (Fig. 9C). However, TRIM56 interference failed to significantly amplify the CD63-LC3 co-localization increase caused by CD147 knockdown (Extended Data Fig. 9C). Exosome quantification analysis paralleled these findings, showing that TRIM56 overexpression effectively reduced CD147 overexpression-induced exosome secretion enhancement (Fig. 9D). Notably, TRIM56 interference did not significantly alter the CD147 knockdown-mediated suppression of exosome secretion (Extended Data Fig. 9D). We propose that these phenomena may be closely associated with intracellular CD147 levels. CD147 downregulation appears to deprive cells of the cellular microenvironment essential for autophagic vesicle formation, which conclusion consistent with our previous research findings. This mechanistic relationship between CD147 expression levels and vesicular trafficking regulation aligns with established paradigms in intracellular membrane dynamics.

A previous study found that TRIM56 downregulation via PCBP1-mediated translational suppression in ovarian cancer [29]. The Cancer Genome Atlas (TCGA) demonstrated PCBP1 overexpression is positive correlation with CD147 expression (*R* = 0.13; *p* = 0.000069) (Extended Data Fig. 9E). We therefore hypothesized CD147-mediated TRIM56 reduction occurs via PCBP1 upregulation. Experimental validation showed: CD147 overexpression significantly increased PCBP1 expression, and CD147 knockdown reduced PCBP1 levels (Fig. 9E). Additionally, PCBP1 depletion reversed CD147-induced TRIM56 suppression in CD147 high cell lines (Fig. 9F, G). These results establish PCBP1 as the mechanistic link between CD147 and TRIM56 downregulation. Additionally, it has been demonstrated in a study on osteoarthritis that during chondrocyte calcification, a significant rise in intracellular calcium concentration promotes the release of calcified EVs (extracellular vesicles), a process accompanied by suppression of the VAMP8/STX17/SNAP29 pathway [30]. Given the parallel observation of cytosolic calcium overload in CD147-regulated exosome release, we postulate that this mechanism may underlie the impact of CD147 on the VAMP8/STX17/SNAP29 pathway. Collectively, our findings suggest that CD147 could regulate the TRIM56 or VAMP/STX17/SNAP29 through multiple mechanisms, though detailed regulatory mechanisms warrant further investigation. Though detailed regulatory mechanisms warrant further investigation.

### CD147-induced amphisomes formation is associated with IP3R3 mediated cytoplasmic calcium overload

**Fig. 10:**
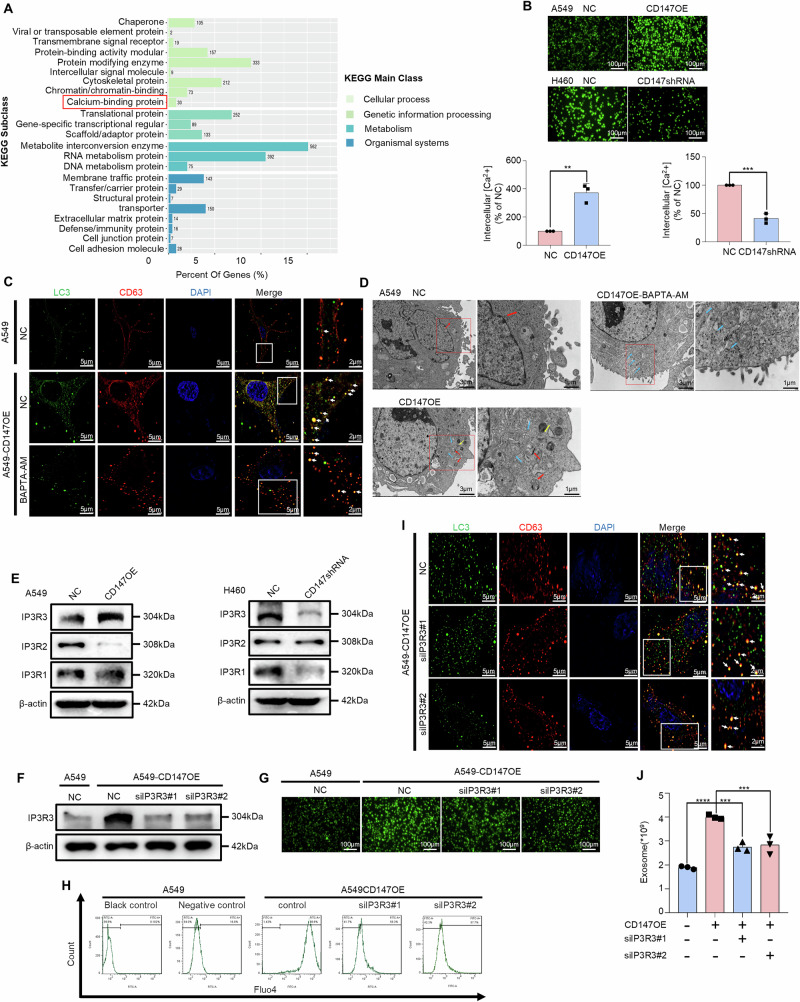
CD147 promotes amphisome biogenesis through IP3R3-mediated calcium homeostasis dysregulation. **A** Transcriptomic profiling of CD147-modulated models reveals differentially expressed calcium signaling components. **B** Quantitative cell calcium imaging using Fluo-4 AM (5 μM): Representative fluorescence micrographs (upper) and ImageJ-based fluorescence intensity quantification (down) in CD147-manipulated systems. **C** Structured illumination microscopy (SIM) demonstrating subcellular colocalization of CD63 (Alexa Fluor™ 555) and LC3 (Alexa Fluor™ 488) in A549 cells treated with calcium chelator BAPTA-AM (30 μM). **D** Transmission electron micrographs documenting autophagic progression: Autophagosomes (red arrows), amphisomes (yellow arrows; autophagosome-MVB hybrids), and multivesicular bodies (green arrows). Scale bars: 5 μm (overview). **E** IP3R1, IP3R2and IP3R3 expression patterns in CD147-manipulated A549 and H460 models assessed by immunoblotting. **F** Western blot analysis of IP3R3 in A549 cells subjected to siRNA-mediated knockdown of IP3R3 (siIP3R3) with concomitant CD147 overexpression (CD147OE). **G** The cells were measured by the fluorescence intensity of Fluo-4 AM (green fluorescence, 5 mM) and observed fluorescence microscope. **H** The cells were measured by the fluorescence intensity of Fluo-4 AM (green fluorescence, 5 mM) and observed by FlowJ. **I** SIM visualization of CD63-LC3 spatial coordination in IP3R3-depleted (siIP3R3) A549 cell models. Scale bars: 5 μm (overview), 2 μm (insets). **J** Exosomal secretion dynamics assessed by ExoCET assay in CD147-OE modelswith IP3R3 perturbation. Secretion levels normalized to 20 μg exosomal protein.

Previous investigations have demonstrated the pivotal regulatory function of CD147 in autophagosome biogenesis. However, a critical question that remains to be addressed is the mechanistic basis underlying the correlation between autophagosomal proliferation and subsequent amphisome generation. Specifically, the causal relationship between autophagosome accumulation and amphisome formation cannot be axiomatically assumed. Given that amphisome biogenesis fundamentally requires the fusion of autophagosomes with MVBs, we hypothesized the existence of potential mediators orchestrating this membrane fusion process. To investigate this mechanism, we performed comprehensive bioinformatics analysis of transcriptomic datasets with particular emphasis on pathway enrichment of differentially expressed genes. Our computational analyses revealed significant enrichment of calcium signaling pathway components among the differentially regulated genes (Fig. 10A and Extended Data Fig. 10A). This finding aligns with prior reports establishing calcium’s role in facilitating autophagosome-polycystic vesicle docking processes [31]. In the current investigation, we identified that CD147 overexpression induces cytoplasmic calcium hyperhomeostasis, whereas CD147 knockdown attenuates intracellular calcium concentrations (Fig. 10B). To functionally validate these observations, we employed immunofluorescence co-localization studies and ultrastructural analysis via TEM. The results demonstrated that calcium chelation using BAPTA-AM significantly attenuated CD147-overexpression-induced LC3-CD63 co-localization signals (Fig. 10C). Conversely, CD147 depletion reduced Mon-mediated calcium activation effects on LC3-CD63 interaction (Extended Data Fig. 10B). Ultrastructural evidence further confirmed that CD147 facilitated amphisome assembly, while BAPTA-AM treatment effectively abrogated this phenomenon (Fig. 10D). Notably, Mon administration rescued amphisome formation in CD147-deficient models (Extended Data Fig. 10C).

Previous studies have established that CD147 potentiates hepatocellular carcinoma progression via upregulation of inositol 1,4,5-trisphosphate receptor type 1 (IP3R1)-mediated cytosolic calcium overload [32]. To investigate functional conservation of this oncogenic axis in pulmonary malignancies, we initially hypothesized that CD147 might similarly regulate IP3R1-dependent calcium signaling in NSCLC. Contrary to this established paradigm, our systematic investigation revealed that CD147 overexpression or knockdown exerted minimal regulatory effects on IP3R1 or IP3R2 expression, but demonstrated profound modulatory effects on its isoform IP3R3 upregulation (Fig. 10E). To mechanistically validate this isoform-specific regulatory axis, we implemented siRNA-mediated IP3R3 knockdown in both CD147-overexpressing A549 cells (Fig. 10F) and endogenously CD147 high H460 cells (Extended Data Fig. 10D). Furo-4 AM calcium imaging and flow cytometric quantification consistently demonstrated that IP3R3 silencing abrogated CD147-induced cytosolic calcium overload (Fig. 10G, H). Normalized pathological calcium concentrations in CD147 high cells (Extended Data Fig. 10F). Notably, IP3R3 silencing significantly attenuated CD147-overexpression and high endogenous CD147-induced LC3-CD63 co-localization signals (Fig. 10I and Extended Data Fig. 10G). Exosomal quantification revealed that genetic suppression of IP3R3 significantly attenuated CD147-overexpression-enhanced exosome secretion (Fig. 10J and Extended Data Fig. 10H). Collectively, these findings establish that CD147-mediated amphisome biogenesis is mechanistically linked to calcium-dependent regulatory pathways.

Our previous findings demonstrated that CD147 induces autophagosome formation via the TRIM56-mediated GCN2/EIF2α/ATG12 pathway and concurrently triggers IP3R3-dependent calcium overload. To investigate potential crosstalk between the IP3R3-regulated calcium signaling axis and the GCN2/EIF2α/ATG12 pathway, we performed gain- and loss-of-function analyses. Specifically, TRIM56 was overexpressed or knocked down in cell lines with either CD147 overexpression or depletion, followed by assessment of IP3R3 expression. Notably, modulation of TRIM56 expression exerted no significant effect on IP3R3 levels in either CD147-manipulated background (Extended Data Fig. 10I, J). Subsequently, we knockdown IP3R3 expression in both CD147-overexpressing cells and cells endogenously expressing high CD147 levels, then detected key components of the GCN2/EIF2α/ATG12 cascade. Strikingly, IP3R3 knockdown did not significantly alter the expression of GCN2 or the ATG12/ATG5 complex in either cellular context. However, subtle alterations in total EIF2α or p-EIF2α levels were observed upon IP3R3 perturbation (Extended Data Fig. 10K, L). Collectively, these results indicate that the CD147-induced GCN2/EIF2α/ATG12 pathway and the IP3R3-dependent calcium pathway represent largely independent signaling axes. While this functional segregation does not preclude all molecular regulation, it underscores the intricate complexity of their regulatory networks.

### CD147 promotes amphisomes formation to increase exosome secretion in mouse Lewis lung carcinoma cells

To validate the translational relevance of these findings in mouse, we established a CD147 overexpression model using murine lung adenocarcinoma cells (LLC) (Extended Data Fig. 11A). Both exosomal marker protein expression profiles and quantitative exosome assays demonstrated that CD147 overexpression significantly enhanced exosome secretion (Extended Data Fig. 11B). Immunofluorescence analysis revealed pronounced co-localization of CD63 with LC3 in CD147-overexpressing cells, indicative of augmented amphisomes formation (Extended Data Fig. 11C). Autophagic flux monitoring via dual-fluorescence labeling (mRFP-GFP-LC3 system) showed increased accumulation of autophagosomes (yellow puncta) upon CD147 overexpression (Extended Data Fig. 11D). Intracellular calcium imaging confirmed that CD147 amplification substantially elevated cytosolic calcium concentrations (Extended Data Fig. 11E). Subsequent pathway validation experiments established that CD147-mediated exosome hypersecretion was mechanistically dependent on GCN2/p-EIF2α/ATG12 activation (Extended Data Fig. 11F), as evidenced by complete abrogation of exosome overproduction following GCN2 and ATG12 knockdown (Extended Data Fig. 11G, H). These experimental outcomes are fully congruent with our previous mechanistic conclusions.

### The proposed model of CD147 promotes exosomes release through activation of crinophagy, thus facilitating tumor progression

**Fig. 11:**
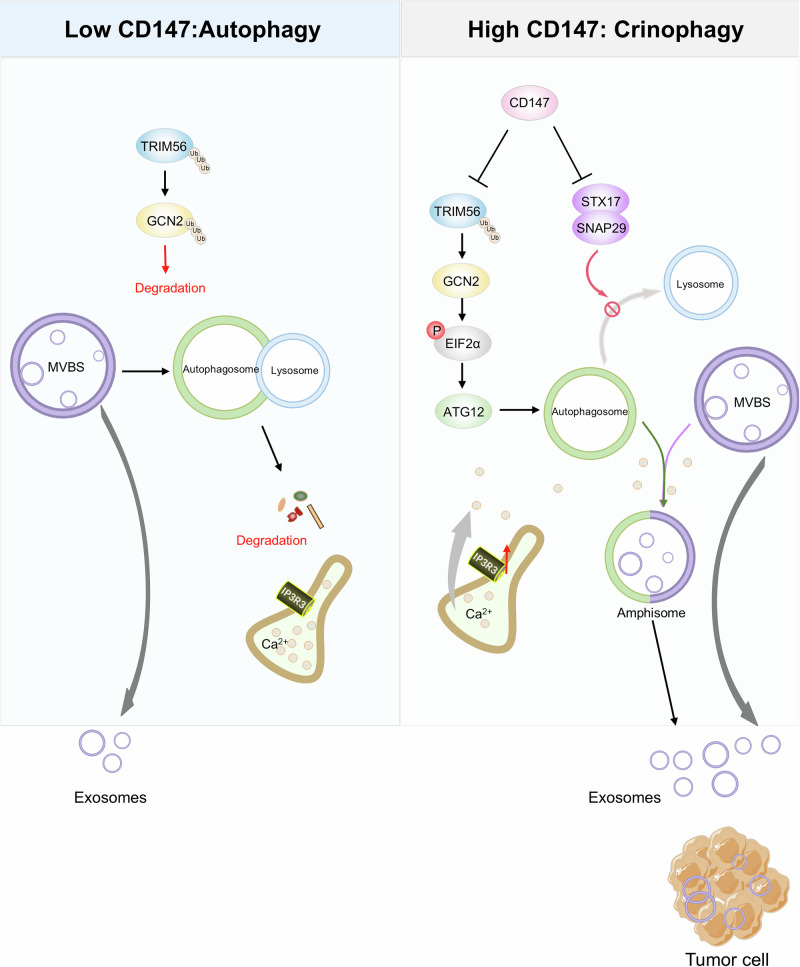
The proposed model of CD147 promotes exosomes release through activation of crinophagy, thus facilitating tumor metastasis. CD147-overexpressing tumor cells establish a calcium-enriched intracellular niche by upregulating IP3R3 that potentiates amphisome biogenesis through enhanced autophagosome-MVBs fusion. This oncoprotein orchestrates a dual regulatory mechanism in autophagy flux: facilitating autophagosome assembly via TRIM56 suppression-mediated GCN2/EIF2α/ATG12 pathway activation, while simultaneously impeding autophagosome-lysosome docking through VAMP8/STX17/SNAP29 downregulation. Ultimately, the CD147-driven exosomal program emerges as a critical metastasis-promoting axis.

As shown in Fig. 11, CD147-overexpressing tumor cells establish a calcium-enriched intracellular niche by upregulating IP3R3, which potentiates amphisome biogenesis through enhanced autophagosome-MVBs fusion. Concurrently, CD147 activated the GCN2/EIF2α/ATG12 signaling axis to drive autophagosome assembly but blocked autolysosome maturation by inhibiting VAMP8/STX17/SNAP29-dependent fusion, thereby supplying essential precursor components for amphisome assembly. The CD147-driven exosomal secretion machinery ultimately accelerates tumor progression.

## Discussion

The oncogenic significance of tumor-derived exosomes in cancer progression has been extensively documented in contemporary literature [33, 34]. Notably, malignant cells demonstrate markedly elevated secretory capacity for exosomal vesicles compared to their non-transformed counterparts [35]. While considerable progress has been made in elucidating exosomal biogenesis, the identification of tumor-associated molecular regulators governing this process remains an unmet challenge in the field. Our systematic investigation employing plasma specimens from NSCLC patients across varying TNM stages revealed a clinically significant positive correlation between exosomal burden and CD147 expression levels. Mechanistic validation studies further identified CD147 as a co-regulator exhibiting functional synergy in potentiating exosomal secretion. Complementing these observations, emerging evidence positions CD147 as a critical mediator of sEV release during malignant reprogramming of colon cancer stem cells [18], thereby corroborating our experimental paradigm.

Subsequently, to investigate the functional implications of CD147-promoted exosome secretion in tumor progression, we co-cultured exosomes derived from diverse cellular sources with multiple tumor cell lines. Through in vitro and in vivo experimentation, we demonstrated that CD147-enriched exosomes exert significant pro-tumorigenic effects. These findings underline the critical importance of elucidating the molecular mechanisms underlying CD147-driven exosome biogenesis, which may establish a pivotal theoretical foundation for developing anti-tumor therapeutic strategies targeting oncogenic exosome suppression.

To elucidate the molecular mechanisms underlying CD147-mediated regulation of exosome release, we engineered transgenic models with CD147 knockdown and overexpression for genome-wide transcriptome profiling. The analytical data implicated the autophagy pathway maybe a potential mechanistic mediator of CD147-dependent exosomal secretion. Given MVBs fate determination—either through plasma membrane fusion for exosome release or autolysosomal degradation—serves as the critical checkpoint in exosome biogenesis [27], we initially hypothesized that CD147 might enhance exosomal output via autophagy suppression. Contrary to this premise, subsequent functional analyses paradoxically revealed CD147-mediated activation of autophagic flux. Ultrastructural examination via TEM coupled with quantitative autophagic flux assays demonstrated that CD147 potentiates autophagosome formation, a finding that directly contradicts the prevailing paradigm wherein autophagy activation typically attenuates exosome production [28]. Through systematic investigation, we resolved this apparent paradox by demonstrating that CD147 selectively activates non-canonical autophagy pathways. Specifically, CD147 expression did not enhance autophagosome-lysosome fusion (a hallmark of complete autophagy), but rather promoted the formation of amphisomes characteristic of crinophagy [36].

Crinophagy, a non-canonical autophagic pathway, is mechanistically defined by impaired autophagosome-lysosome fusion and autophagosome-MVBs interactions [37]. This process has been implicated in extracellular vesicle-mediated pathophysiological processes, including neoplastic invasion [12], ectopic calcification [30] and so on. In the present study, SIM microscopy and TEM analyses conclusively demonstrated CD147 overexpression-induced combination of autophagosomes and amphisomes. Meanwhile, CD147-mediated inhibition of STX17 and SNAP29 expression, which are SNARE complex components critically required for autophagosome-lysosome fusion [38, 39]. Intriguingly, using autophagy modulators to validate CD147-mediated amphisome formation and exosome release, we observed opposing responses to rapamycin (RAP) in A549 and H460 cells. RAP is a classical inducer of autophagy, which functions by promoting the formation of autophagosomes and autophagolysosomes. As shown in Fig. 1G, evaluation of CD147 expression in various lung cancer cell lines and identified A549 as having low CD147 expression, while H460 exhibited high CD147 expression. Given our findings that CD147 promotes autophagosome formation but inhibits autophagosome-lysosome fusion, we thought this discrepancy may be due to endogenous CD147 expression differences. Low CD147 expression in A549 cells enhanced RAP-induced MVBs degradation, thereby suppressing exosome release. Conversely, high CD147 expression in H460 cells promoted secretory autophagy, subsequently augmenting exosomal secretion. Autophagic flux assays supported this mechanism: A549 showed complete autophagic progression after RAP treatment, while H460 exhibited impaired autophagic flux (Fig. 5A and Extended Data Fig. 5A). These observations establish CD147 as an essential molecular determinant of amphisome biogenesis. Furthermore, our results found targeting VAMP8, STX17, and SNAP29 in CD147-overexpressing significantly decreased the CD147-mediated exosome release patterns, which consistent with prior reports wherein VAMP8/SNAP29 promotion significantly reduced sEV secretion [40]. In our present study, we found that CD147 regulates exosome release through multiple mechanisms—including disrupted calcium homeostasis, impaired autolysosome formation, and amphisome accumulation. Activation of the VAMP8/STX17/SNAP29 complex may promote autolysosome formation, which would enhance MVBs degradation and consequently inhibit exosome release. However, we observed that the rescue group released more exosome than the untreated control. Given that CD147-mediated regulation of exosome release results from multifaceted mechanisms, solely activation of VAMP8/STX17/SNAP29 is insufficient to completely abrogate the pro-exosomal activity of CD147.

To delineate the upstream signaling governing CD147-driven autophagosome formation, we integrated transcriptomic profiling with literature curated pathway analyses, identifying four potential regulatory axes: PI3KC2B/AKT [41], P53/AMPK [42], MAPK/ATG7 [43], and GCN2/EIF2α/ATG12 [44]. Western blot validation confirmed selective activation of the GCN2/EIF2α/ATG12 cascade under CD147-overexpressing conditions. Through combinatorial genetic silencing (GCN2/ATG12) and pharmacological inhibition of pEIF2α dephosphorylation (SAL003), we unequivocally established CD147’s function in autophagic vesicle formation and exosomal secretion through this signaling axis. Notably, SAL003 administration in CD147-deficient systems elicited counterintuitive outcomes: Enhanced autophagosome generation concomitant with attenuated exosome secretion. This apparent paradox may be mechanistically reconciled through CD147’s dual regulatory capacity-while facilitating autophagosome biogenesis, it concurrently suppresses autolysosomal maturation by downregulating fusion machinery components. SAL003-mediated autophagosome formation in CD147-ablated systems permitted unimpeded autophagosome-lysosome convergence, thereby activating canonical degradative autophagy. This finding aligns with established autophagic flux-exosome secretion antagonism, wherein augmented lysosomal degradation inversely correlates with exosomal output [45]. The mechanistic coherence of our model is further substantiated by literature-documented roles of EIF2α phosphorylation in upregulating ATG12 expression both transcriptional and translational levels [46] and exerting tumor-suppressive effects in pulmonary adenocarcinoma [47]. This multilayered experimental evidence positions CD147 as a master regulator interfacing non-degradative autophagy programs with exosome secretion dynamics, providing novel insights into tumor cell adaptive plasticity.

Our ongoing investigation into the mechanistic underpinnings of CD147-mediated regulation of the GCN2/EIF2α/ATG12 pathway has revealed significant progress. Through proteomic profiling analysis, we identified four potential E3 ligases associated with GCN2: NEDD4L, ITCH, TRIM56, and TRIM68. Initial functional validation via expression vector construction and co-transfection assays suggested regulatory potential in all four candidates. Notably, while NEDD4L has been previously established as a canonical E3 ligase for GCN2 [48], comprehensive evaluation in CD147-knockdown and CD147-overexpressing models demonstrated TRIM56 to be a novel E3 ligase critically involved in CD147-mediated GCN2 pathway activation. Crucially, our data provide the first evidence that TRIM56 specifically mediates ubiquitin conjugation at lysine 619 (K619) of GCN2, thereby facilitating its ubiquitination. This discovery represents the inaugural documentation of TRIM56’s E3 ligase activity toward GCN2 within the ubiquitin-proteasome system.

While our investigations have conclusively demonstrated CD147’s dual capacity to potentiate autophagosome biogenesis and suppress autolysosomal maturation, it is imperative to emphasize that autophagosome accumulation does not mechanistically necessitate amphisome formation. This observation underscores that amphisome biogenesis constitutes a regulated process distinct from mere autophagosome quantity elevation. To delineate the underlying mechanisms, we performed systematic transcriptomic profiling, revealing calcium signaling pathways as critical regulatory nodes. This finding aligns with established literature: Prior studies have established that calcium-dependent molecular machinery governs the docking of autophagosomes with MVBs-the precursor compartments of exosomes-during vesicular trafficking [31, 49]. Strikingly, our functional analyses demonstrated that CD147 promotes amphisome assembly through intracellular calcium overload. This calcium dysregulation-mediated amphisome generation mechanistically links to CD147’s broader role in driving crinophagy, a process wherein autophagic components are extracellularly routed rather than lysosomal degraded.

Building upon these findings, we further demonstrated that CD147 promotes cytosolic calcium overload by upregulating IP3R3, a calcium channel protein localized to the endoplasmic reticulum membrane that mediates calcium homeostasis between the endoplasmic reticulum and cytosol. While our laboratory previously identified CD147-mediated calcium regulation via IP3R1 in hepatocellular carcinoma models [32], the current study failed to recapitulate this mechanism in lung cancer systems—a discrepancy potentially attributable to tumor heterogeneity. Notably, our data align with clinical observations that elevated IP3R3 expression drives lung cancer progression [50, 51]. Through systematic interrogation of IP3R3 functionality, we conclusively established that IP3R3-mediated calcium dyshomeostasis plays a pivotal role in CD147-induced autophagic vesicle formation. This mechanistic link between CD147, calcium flux regulation, and autophagic machinery activation provides novel insights into tumor-specific metabolic adaptations. However, the precise molecular mechanisms through which CD147 modulates IP3R3 activity to regulate cytosolic calcium dynamics remain incompletely characterized and necessitate further rigorous investigation.

Our current findings position CD147 as a master regulatory nexus coordinating oncogenic exosome biogenesis through three synergistic axes: [1] proteostatic regulation of GCN2/EIF2α/ATG12 signaling via TRIM56-mediated K619 ubiquitination (a novel E3 ligase activity identified in this study), and [2] IP3R3-dependent calcium dyshomeostasis precipitating amphisome formation. [3] Impairment of VAMP8/STX17/SNAP29-dependent autophagolysosome formation. This bifunctional mechanism drives crinophagic vesicular trafficking while subverting canonical autolysosomal degradation, thereby establishing an autophagy-exosome secretion coupling phenomenon that fuels cancer progression. The discovery of CD147’s dual modulation of ubiquitination circuitry and organellar calcium flux not only redefines its role as a tumor-associated antigen but unveils therapeutic vulnerabilities at the intersection of vesicular trafficking and metabolic adaptation.

## Supplementary information

Extended Data Figure Data

Mass spectrum data of plasma exosome

Complete transcript information of E3 ligase and GCN2

Unprocessed western blots

## Data Availability

All data presented and source data in this study are included in entire article and Supplementary Material and Extended Data. Further communication is recognized to the corresponding authors once there are any questions.
