# Supplementary material for: CD147 promotes NSCLC metastasis by inducing secretory autophagy-dependent exosome secretion via TRIM56-mediated ubiquitination and degradation of GCN2
Source: Cell Death Differ. 2025 Dec 18;33(6):1152–74. doi: 10.1038/s41418-025-01636-y (PMC13247162; doi:10.1038/s41418-025-01636-y)
Supplement: Supplementary file 1 — Extended Data Figure Data [file 41418_2025_1636_MOESM1_ESM.docx]

**
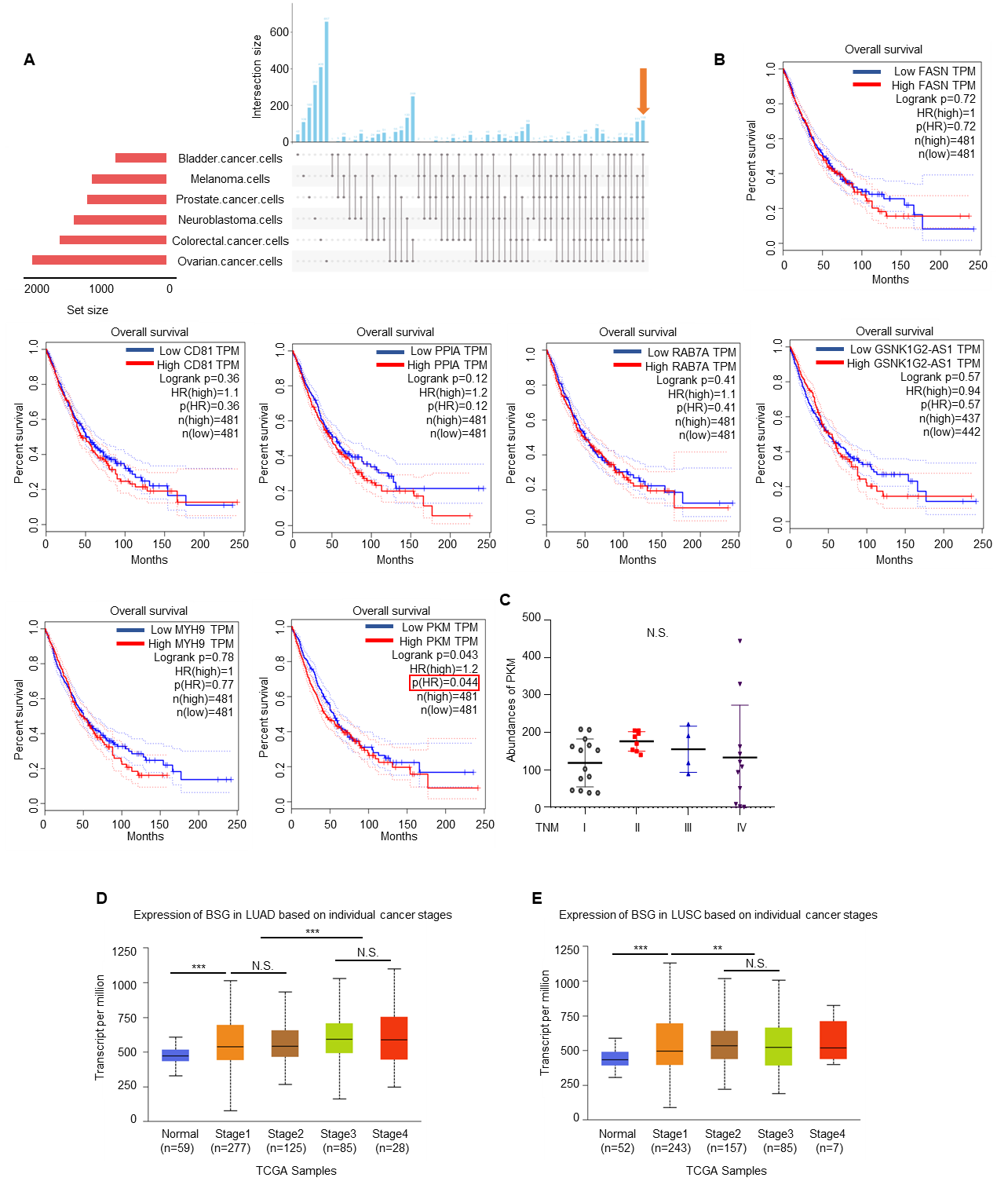
**

**
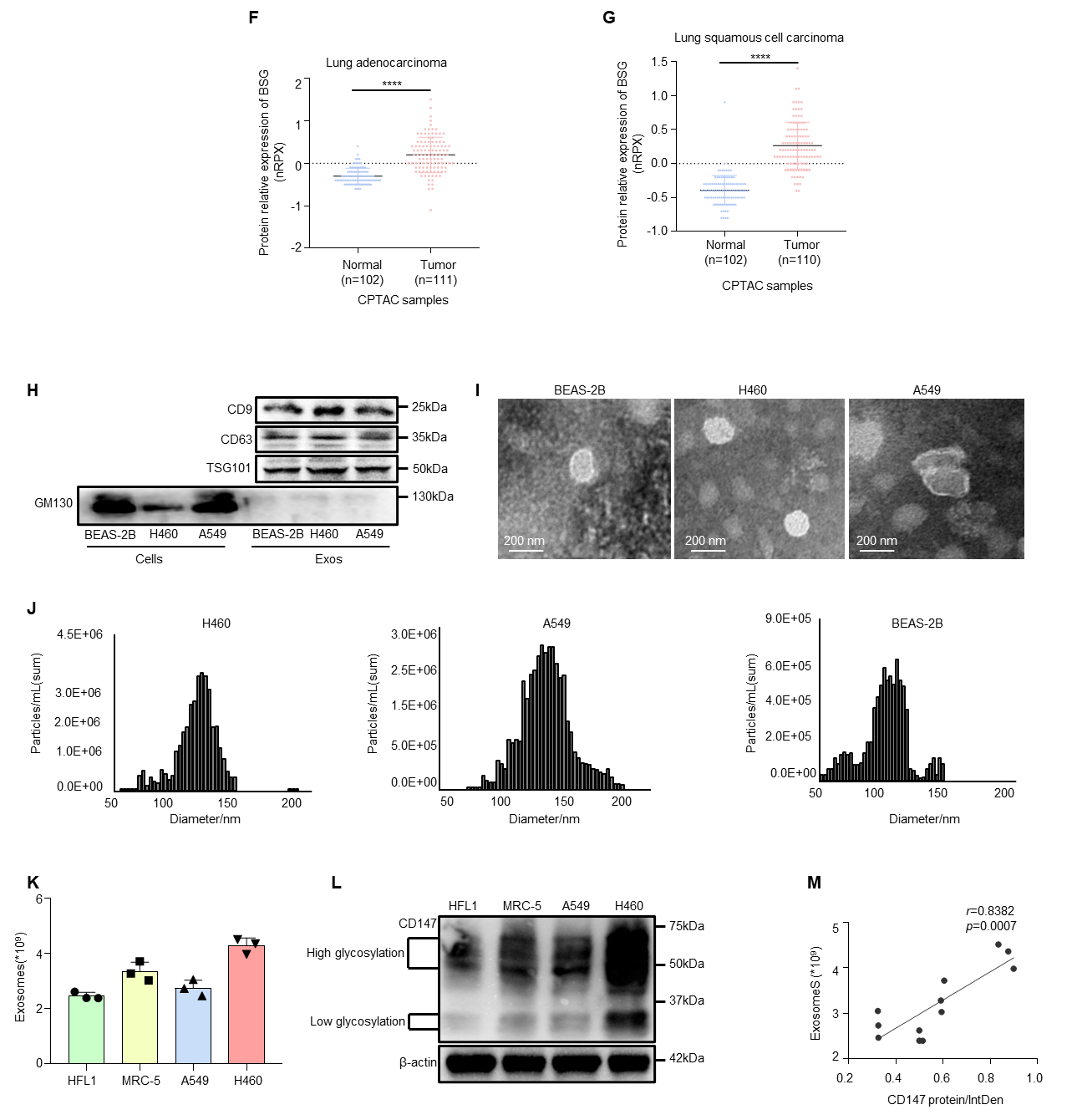
**

**Extended Data Fig. 1 Database analysis and exosomes concentration information.** **(A)** Comparative proteomic analysis of tumor-derived exosomes: UpSet plot visualization of shared proteins across multiple cancer types (ExoCarta database). The red arrow indicates the number of exosomal proteins shared by different cancer cells. **(B)** Overall survival rates of individuals with high and low potential genes were determined by Kaplan–Meier analysis. **(C)** TNM-stage-associated enrichment of exosomal PKM via quantitative mass spectra. **(D, E)** The transcript levels of BSG (CD147) in LUAD and LUSC based on different TNM stage from TCGA database. **(F, G)** The protein expression of BSG (CD147) proteomic profiling in LUAD and LUSC from the CPTAC database. **(H)** Immunoblot validation of exosomal markers (CD9/CD63/TSG101) and cellular contamination control (GM130 absence). **(I)** Transmission electron micrographs of exosomes isolated from bronchial (BEAS-2B) and NSCLC (H460/A549) cell lines. Scale bars: 200 nm. **(J)** Nanoparticle tracking analysis (NTA) showing exosome size distribution (mode: 120±30 nm). **(K)** Exosomal secretion profiles quantified by Exo-CET assay normalized to 20 μg exosomal protein (BCA quantification) **(L)** Immunoblot validation of CD147 expression across cellular models: Lung adenocarcinoma lines (A549, H460) versus normal lung fibroblasts (MRC-5, HFL1). β-actin served as loading control. **(M)** Spearman correlation analysis between the protein grayscale value of CD147 and the number of exosomes from different cell lines.


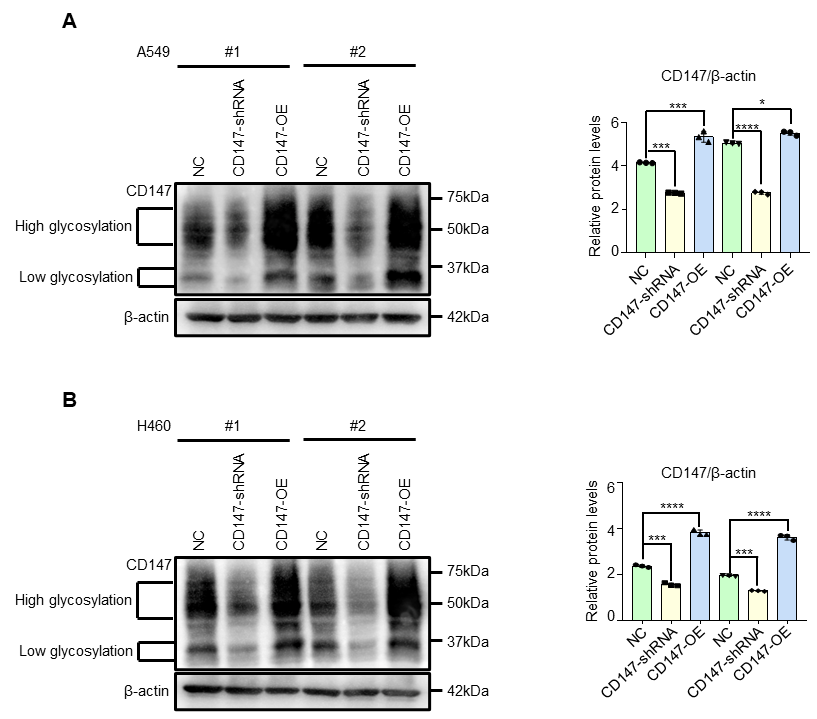


**Extended Data Fig. 2 Systematic characterization of CD147 expression modulation in NSCLC cellular models.** **(A, B)** Left: Immunoblot analysis of CD147 expression profiles in A549 and H460 cell lines under genetic manipulation; Fig. 2 Systematic characterization of CD147 expression modulation in NSCLC cellular models. Right: Densitometric quantification normalized to β-actin loading control. Experimental groups: NC (negative control), KD (shRNA-mediated CD147 knockdown), OE (CD147 overexpression).


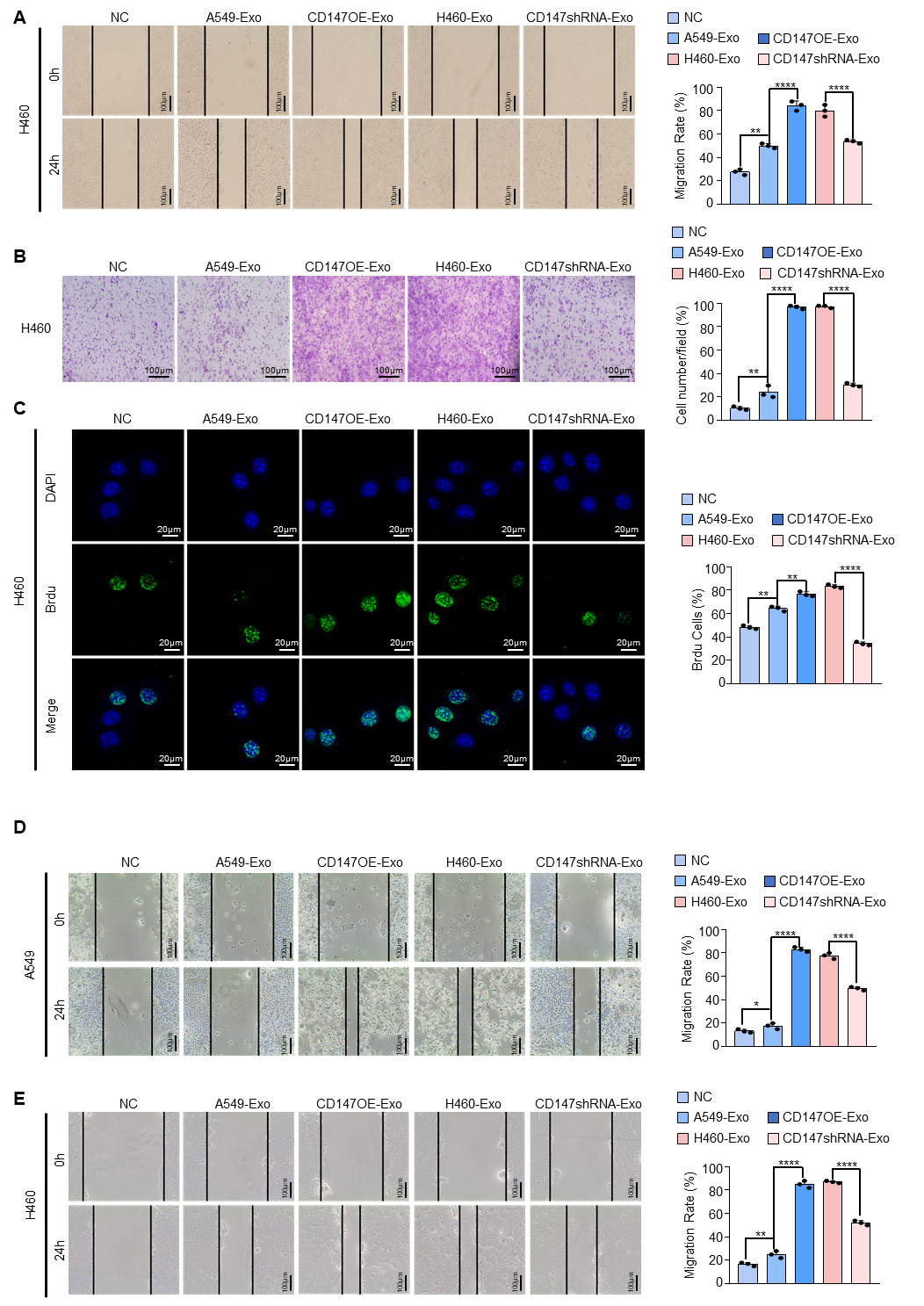

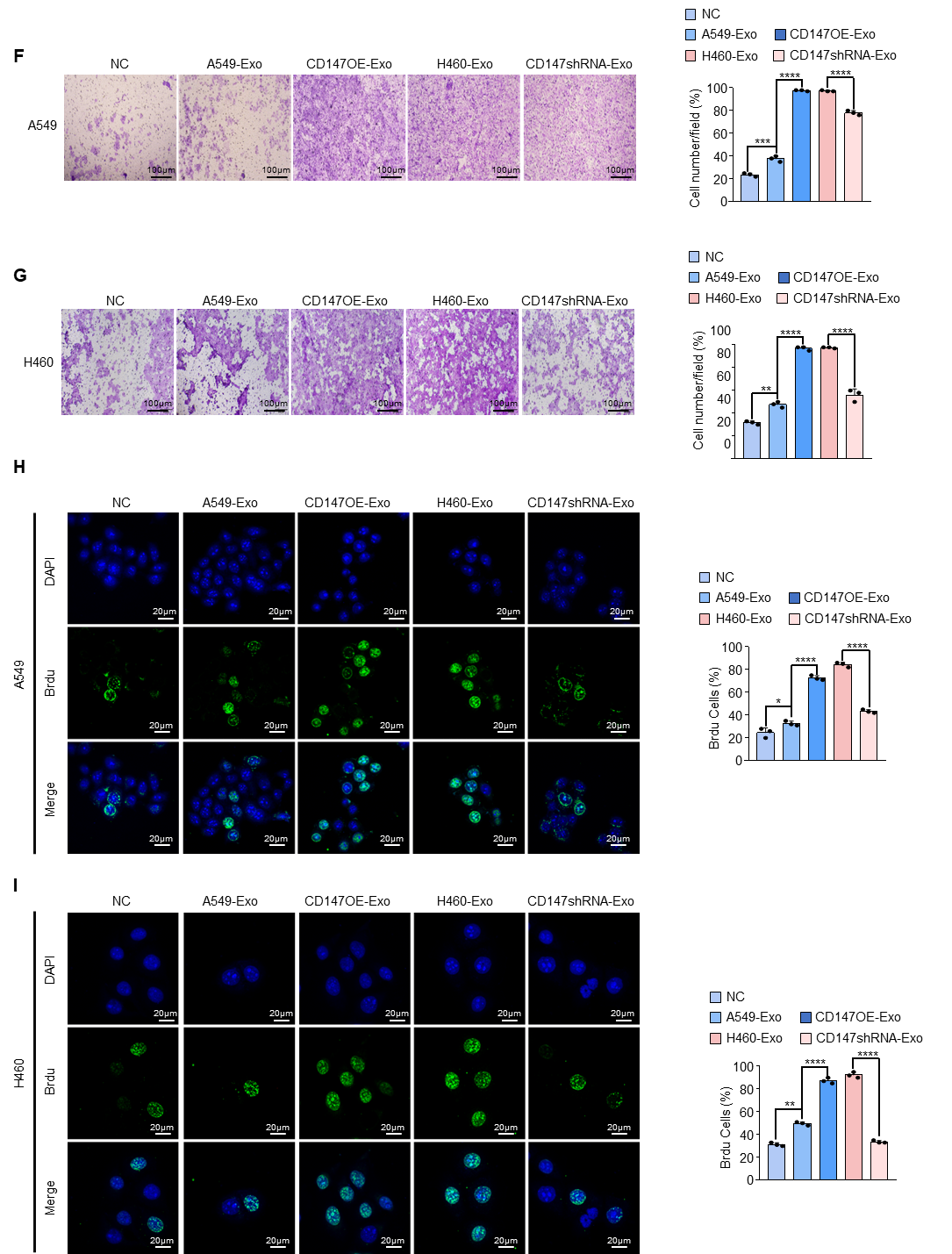

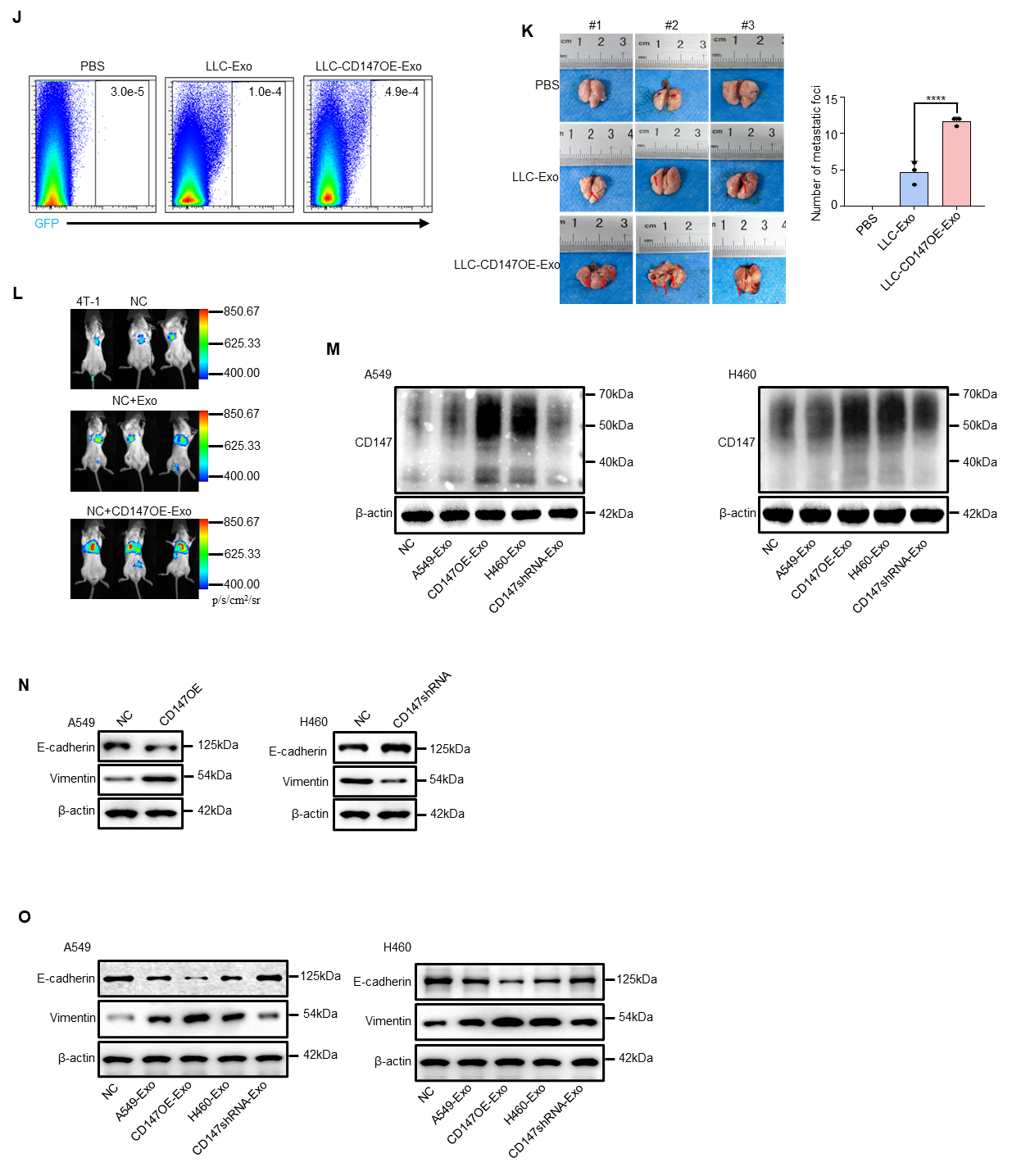
**Extended Data Fig. 3 Exosomes derived by CD147 facilitate metastatic progression through enhanced cellular metastatic potential.** **(A)** Exosomal CD147 enhances migratory capacity. Left: Phase-contrast images of wound closure in H460 monolayers treated with exosomes from indicated groups (0/24h); Right: Quantification of wound healing rates. **(B)** Chemotaxis potentiation by CD147-derived exosomes. Transwell membranes post 24h migration. Migrated cell counts normalized to control. **(C)** Exosomal CD147 enhances proliferation capacity. Left: Fluorescence microscope of Brdu in H460 cells treated with exosomes from indicated groups; Right: Quantification of Brdu positive cell proportion. **(D-I)** Effect of different exosome concentrations on tumor progression; approximate concentrations: A549: 36 μg; A549-CD147OE: 70 μg; H460: 58 μg; H460-CD147shRNA: 42 μg; Left: phase-contrast images or fluorescence microscope; Right: Quantification of corresponding images. **(J)** CD147-derived exosomes promote pulmonary colonization. Quantification of GFP^+^ LLC metastatic foci in lungs. **(K)** Representative images of lung metastases in metastatic tumors. The lung metastases from each mouse were counted. **(L)** Systemic tolerance to exosomal treatment. Lung metastasis effects of CD147-derived exosomes. Representative fluorescence images of luciferase signals captured from lung metastasis cancer cells are shown. **(M)** Endogenous CD147 expression in the receptor cell. CD147 expression in A549 and H460 cells treated with exosomes from indicated groups were detected via immunoblotting. **(N)** The EMT-related protein molecules were analyzed via immunoblotting in A549/H460 cells treated with CD147 overexpression or knockdown. **(O)** The EMT-related protein molecules were analyzed via immunoblotting in A549 and H460 cells treated with exosomes from indicated groups.


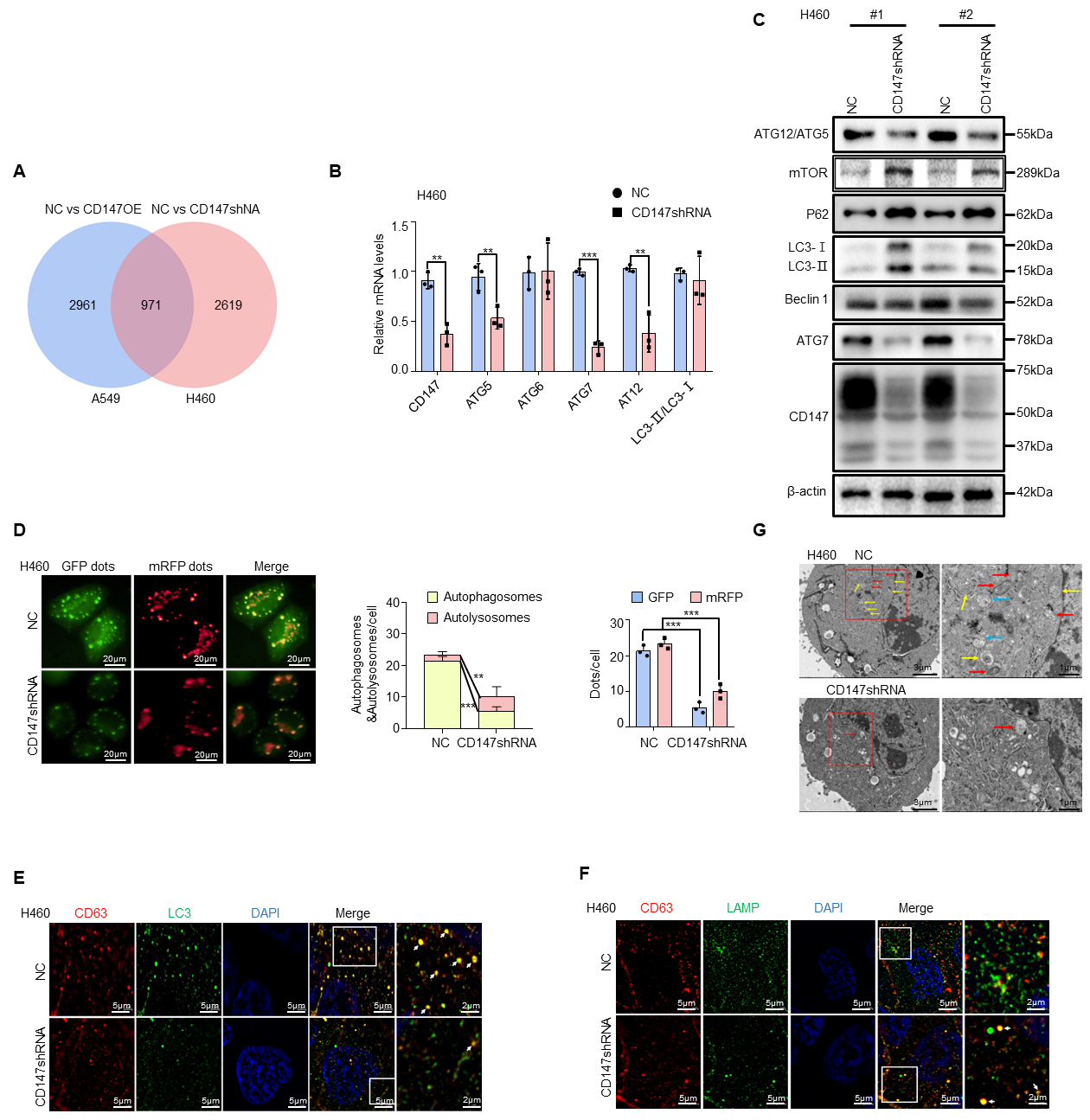
**Extended Data Fig. 4** **CD147 promotes amphisome formation in NSCLC. (A)** Transcriptomic Profiling of CD147-modulated Pathways. Venn intersection of differential genes altered by CD147 manipulation. **(B)** Validation of Autophagic Flux Regulation. qRT-PCR analysis of autophagy-related transcripts normalized to β-actin; **(C)** Immunoblot of autophagy-related proteins levels. Densitometry quantified versus β-actin. **(D)** Spatiotemporal Autophagosome-Lysosome Dynamics. Left: Fluorescence microscope of RFP-GFP-LC3 reporter cells. Yellow puncta: autophagosomes (RFP^+^GFP^+^); Red puncta: autolysosomes (RFP^+^GFP^−^); Right: Quantification of autophagic flux index. **(E, F)** Super-resolution Imaging of Organelle Crosstalk. Structured illumination microscopy (SIM) of CD63^+^ MVBs (Alexa Fluor 555, red) co-localizing with LC3^+^ autophagosomes and LAMP^+^ lysosome (Alexa Fluor 488, green). Scale bars: 2 μm. **(G)** Ultrastructural Evidence of Amphisome Biogenesis. Transmission electron micrographs showing: Red arrows–autophagosomes; Yellow arrows–amphisomes (autophagosome-MVB hybrids); Green arrows–multivesicular bodies (MVBs). Scale bars: 5 μm (overview).


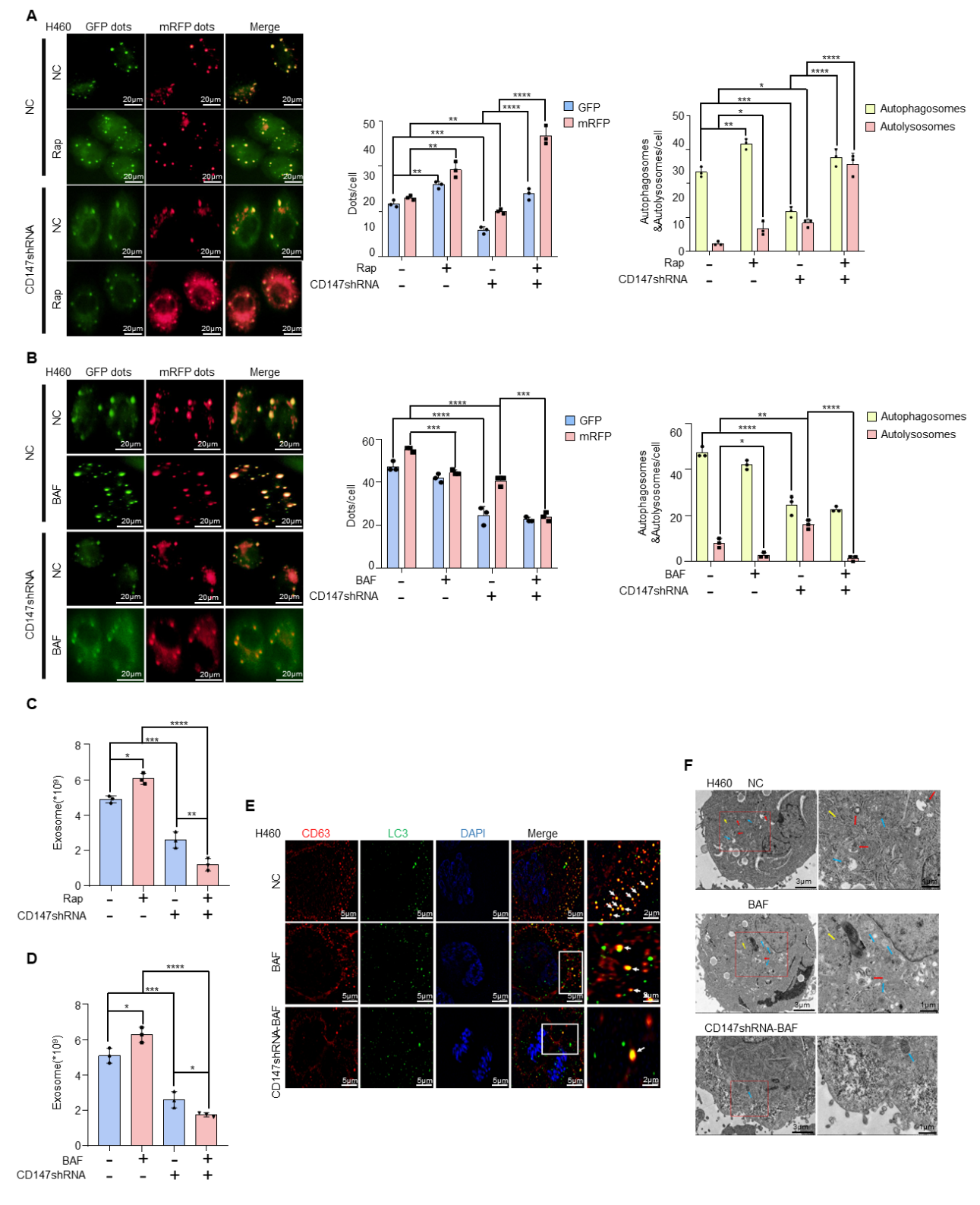
**Extended Data Fig. 5 CD147 is essential for amphisomes formation. (A-B)** Cells were transduced with lentiviral vectors expressing RFP-GFP-LC3 reporters. Representative fluorescence micrographs demonstrate subcellular localization patterns, with quantitative analysis of autophagic puncta: yellow signals (autophagosomes, GFP^+^RFP^+^) and red signals (autolysosomes, GFP^-^/RFP^+^). Data represent mean ± SEM. **(C-D)** Exosome secretion profiles of A549 and H460 cells under CD147 modulation were analyzed using ExoCET exosome quantification assay. Cells were subjected to CD147 overexpression or shRNA-mediated knockdown combined with Rapamycin (200 nM) or Bafilomycin A1 (100 nM) treatment. Exosome concentrations were normalized to 20 μg of exosomal protein. **(E)** Super-resolution microscopy (SIM) images showing spatial colocalization of CD63 (Alexa Fluor™ 555) and LC3(Alexa Fluor™ 488) in membrane compartments. Scale bars: 5 μm (overview), 2 μm (insets). **(F)** Ultrastructural evidence of autophagic flux by transmission electron microscopy. Red arrows–autophagosomes; Yellow arrows–amphisomes (autophagosome-MVB hybrids); Green arrows–multivesicular bodies (MVBs). Scale bars: 5 μm (overview).

**
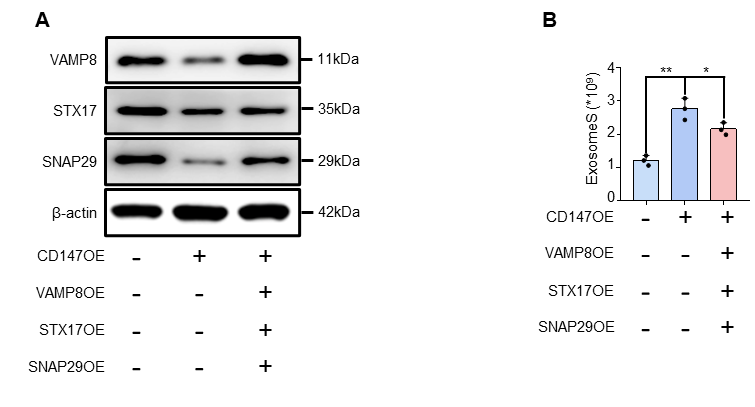
**

**Extended Data Fig. 6** **Autolysosome maturation induced by VAMP8/STX17/SNAP29 activation abrogate CD147-dependent exosome secretion (A)** Western blot (Left) and quantitative analysis (Right) of STX17, VAMP8, and SNAP29 protein expression in corresponding A549 cell line under CD147 overexpression and corresponding STX17/VAMP8, /SNAP29 co-expression by using lentiviral transduction. **(B)** Exosome secretion profiles of A549 cells under CD147 overexpression and corresponding STX17/VAMP8, /SNAP29 co-expression were analyzed using ExoCET exosome quantification assay.

**
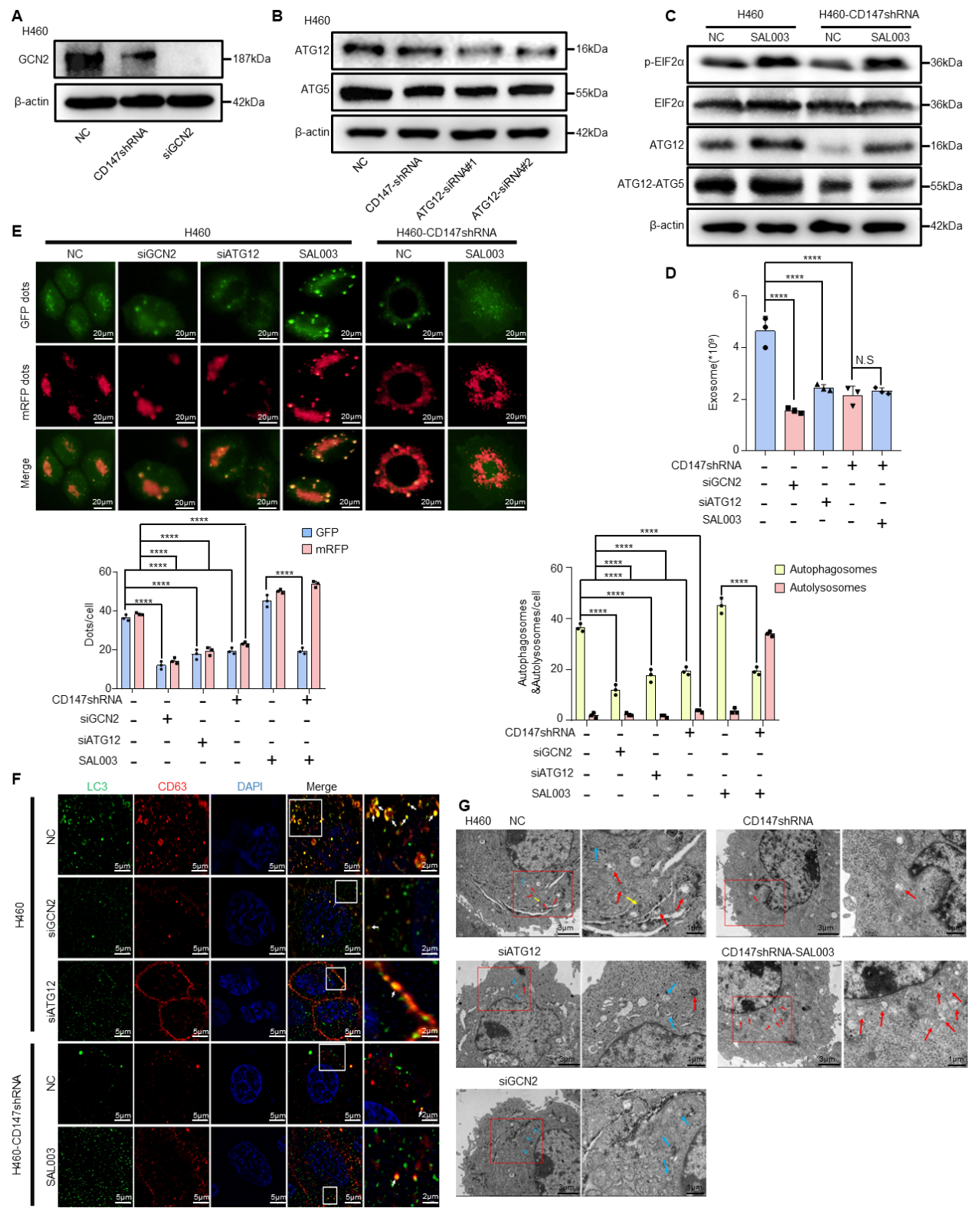
**

**Extended Data Fig. 7** **Pharmacological and genetic treatment of the GCN2/EIF2α/ATG12 axis compromises CD147-mediated amphisome formation (A, B)** Immunoblot validation of pathway dynamics in H460 cells following ATG12 or GCN2 silencing. **(C)** Pharmacological activation of EIF2α signaling using SAL003 in H460-CD147shRNA (CD147 shRNA) cells. **(D)** Exosomal secretion dynamics quantified by ExoCET assay in H460 cells under combinatorial modulation of CD147 (shRNA-mediated knockdown) with pathway perturbations (siGCN2/siATG12/SAL003). Data normalized to 20 μg exosomal protein. **(E)** Dual-fluorescence microscopy of RFP-GFP-LC3 reporters: Representative images (upper) and quantitation of autophagic compartments (down). Yellow puncta (GFP^+^RFP^+^): autophagosomes; Red puncta (GFP^-^RFP^+^): autolysosomes. **(F)** Super-resolution microscopy (SIM) images showing spatial colocalization of CD63 (Alexa Fluor™ 555) and LC3(Alexa Fluor™ 488) in membrane compartments. Scale bars: 5 μm (overview), 2 μm (insets). **(G)** Ultrastructural evidence of autophagic flux by transmission electron microscopy. Red arrows–autophagosomes; Yellow arrows–amphisomes (autophagosome-MVB hybrids); Green arrows–multivesicular bodies (MVBs). Scale bars: 5 μm (overview).

**
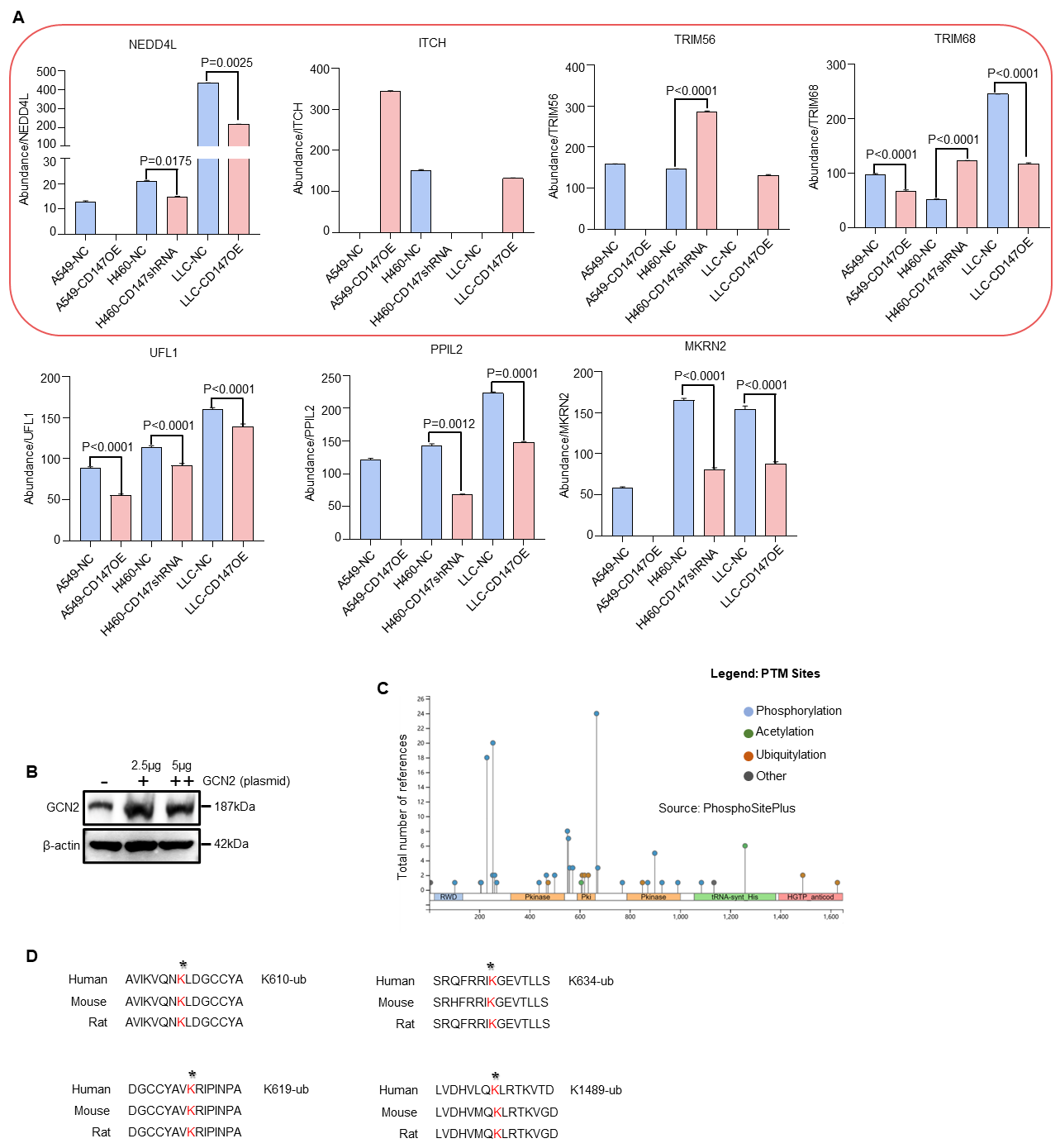
**

**Extended Data Fig. 8 Proteomic characterization of GCN2 ubiquitination regulation.** **(A)** Proteomic profiling identified candidate E3 ubiquitin ligases interacting with GCN2 through multi-platform LC-MS/MS validation across multiple cell lines. **(B)** Dose-dependent immunoblot validation of GCN2 expression in HEK293T cells transiently transfected with increasing concentrations of GCN2 expression plasmids (2.5, 5 μg), with 2.5 μg selected for optimal expression in subsequent assays. **(C, D)** Ubiquitination landscape characterization of GCN2: In silico prediction of lysine ubiquitination sites; Phylogenetic conservation analysis of critical ubiquitination residues across vertebrate species.

**
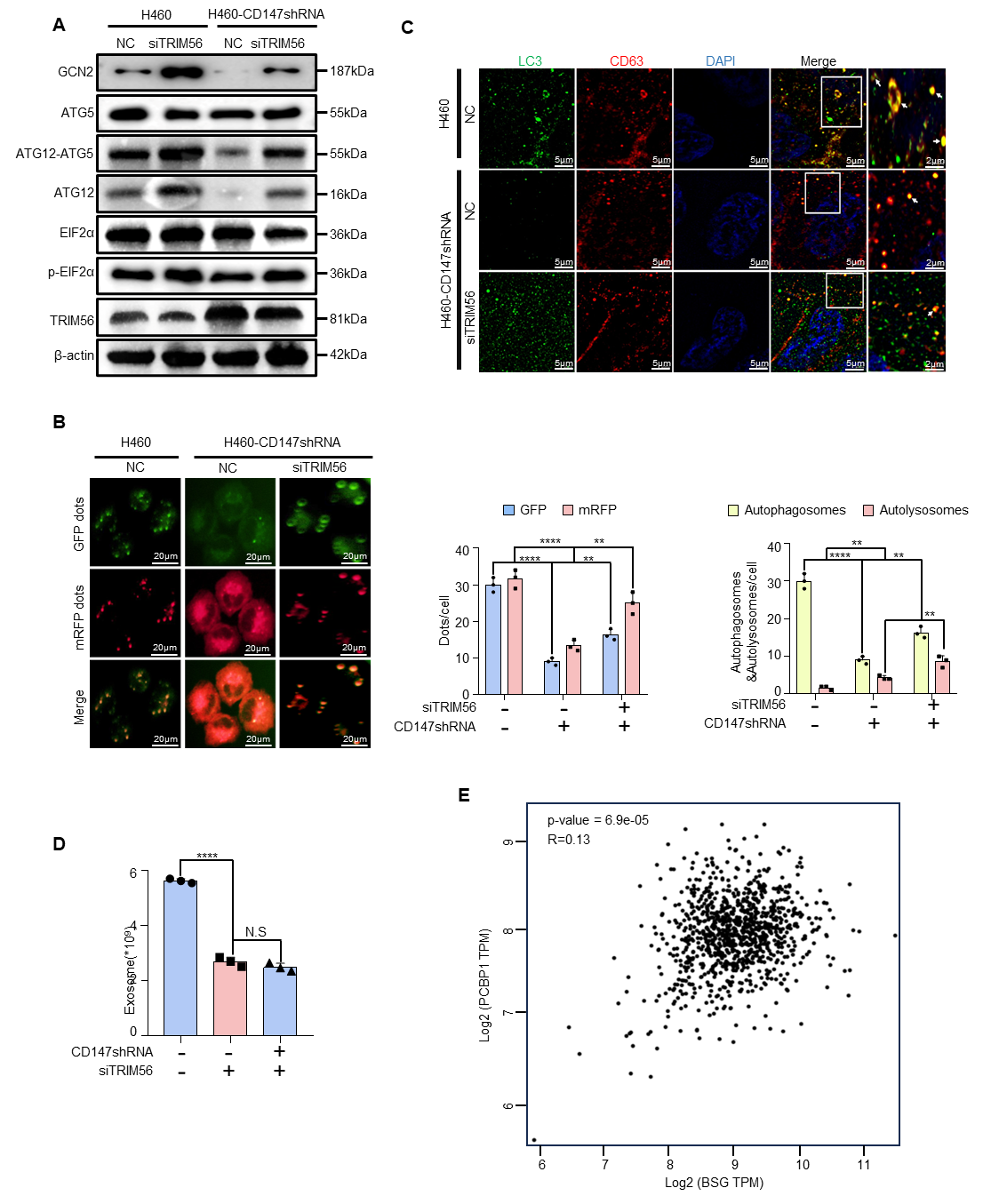
**

**Extended Data Fig. 9 TRIM56 suppresses CD147-mediated activation of the GCN2/EIF2α/ATG12 axis and exosome release.** **(A)** Immunoblot validation of pathway inhibition in H460-CD147shRNA (CD147 knockdown) cells subjected to TRIM56 silencing (siTRIM56). **(B)** Dual-fluorescence imaging of autophagic progression in H460-CD147shRNA models under TRIM56 depletion, with puncta quantification normalized to cytoplasmic area. **(C)** Super-resolution microscopy (SIM) images showing spatial colocalization of CD63 (Alexa Fluor™ 555) and LC3(Alexa Fluor™ 488) in membrane compartments. Scale bars: 5 μm (overview), 2 μm (insets). **(D)** Exosomal secretion profiles assessed by ExoCET assay in CD147-manipulated models (OE: overexpression; shRNA: knockdown) with concomitant TRIM56 modulation. Secretion levels normalized to 20 μg exosomal protein. **(E)** TCGA database was used to analyze the correlation between CD147 and PCBP1 in lung cancer. A Pearson value (R=0.13) > 0 indicates a positive correlation. p<0.05 represents statistically significant differences.


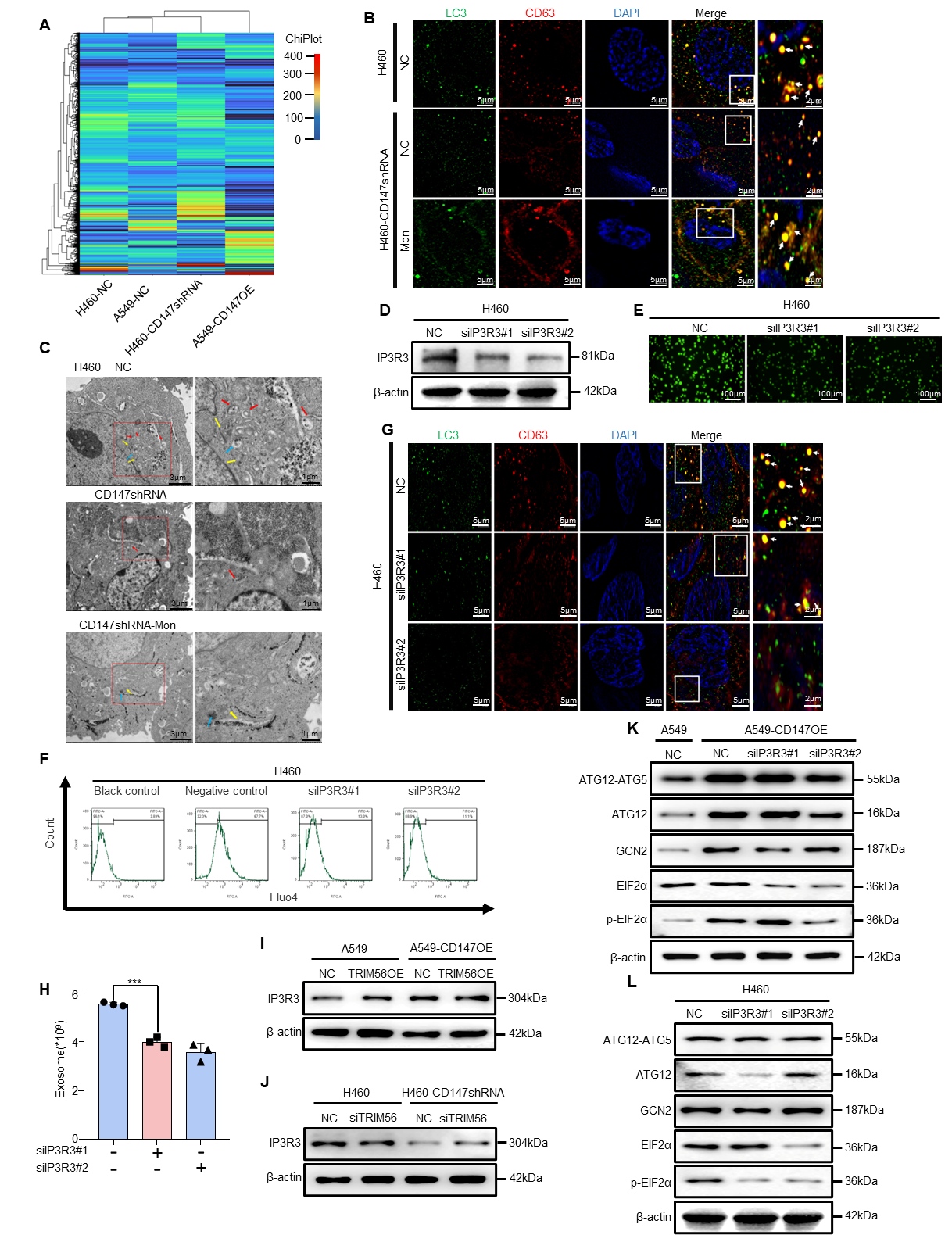
**Extended Data Fig. 10 IP3R3-mediated calcium overload promotes CD147-induced amphisome formation. (A)** Heat map of transcriptomic profiling of CD147-modulated models. **(B)** Structured illumination microscopy (SIM) demonstrating subcellular colocalization of CD63 (Alexa Fluor™ 555) and LC3 (Alexa Fluor™ 488) in H460 cells treated with calcium activator Mon (7 μM). **(C)** Transmission electron micrographs documenting autophagic progression: Autophagosomes (red arrows), amphisomes (yellow arrows; autophagosome-MVB hybrids), and multivesicular bodies (green arrows). Scale bars: 5 μm (overview). **(D)** Western blot analysis of IP3R3 in H460 cells subjected to siRNA-mediated knockdown of IP3R3 (siIP3R3). **(E)** The cells were measured by the fluorescence intensity of Fluo-4 AM (green fluorescence, 5 mM) and observed fluorescence microscope. **(F)** The cells were measured by the fluorescence intensity of Fluo-4 AM (green fluorescence, 5 mM) and observed by FlowJ. **(G)** SIM visualization of CD63-LC3 spatial coordination in IP3R3-depleted (siIP3R3) H460 cell models. Scale bars: 5 μm (overview), 2 μm (insets). **(H)** Exosomal secretion dynamics assessed by ExoCET assay in CD147-OE models (or H460 cells) with IP3R3 perturbation. Secretion levels normalized to 20 μg exosomal protein (BCA quantification). **(I)** Western blot analysis of IP3R3 in A549 and A549-CD147OE cells subjected to TRIM56 overexpression. **(J)** Western blot analysis of IP3R3 in H460 and H460-CD147shRNA cells subjected to siRNA-mediated knockdown of TRIM56 (siTRIM56). **(K)** Western blot analysis of GCN2/EIF2α/ATG12 pathway in A549 cells subjected to siRNA-mediated knockdown of IP3R3 (siIP3R3) with concomitant CD147 overexpression (CD147OE). **(L)** Western blot analysis of GCN2/EIF2α/ATG12 pathway in H460 cells subjected to siRNA-mediated knockdown of IP3R3 (siIP3R3).

**
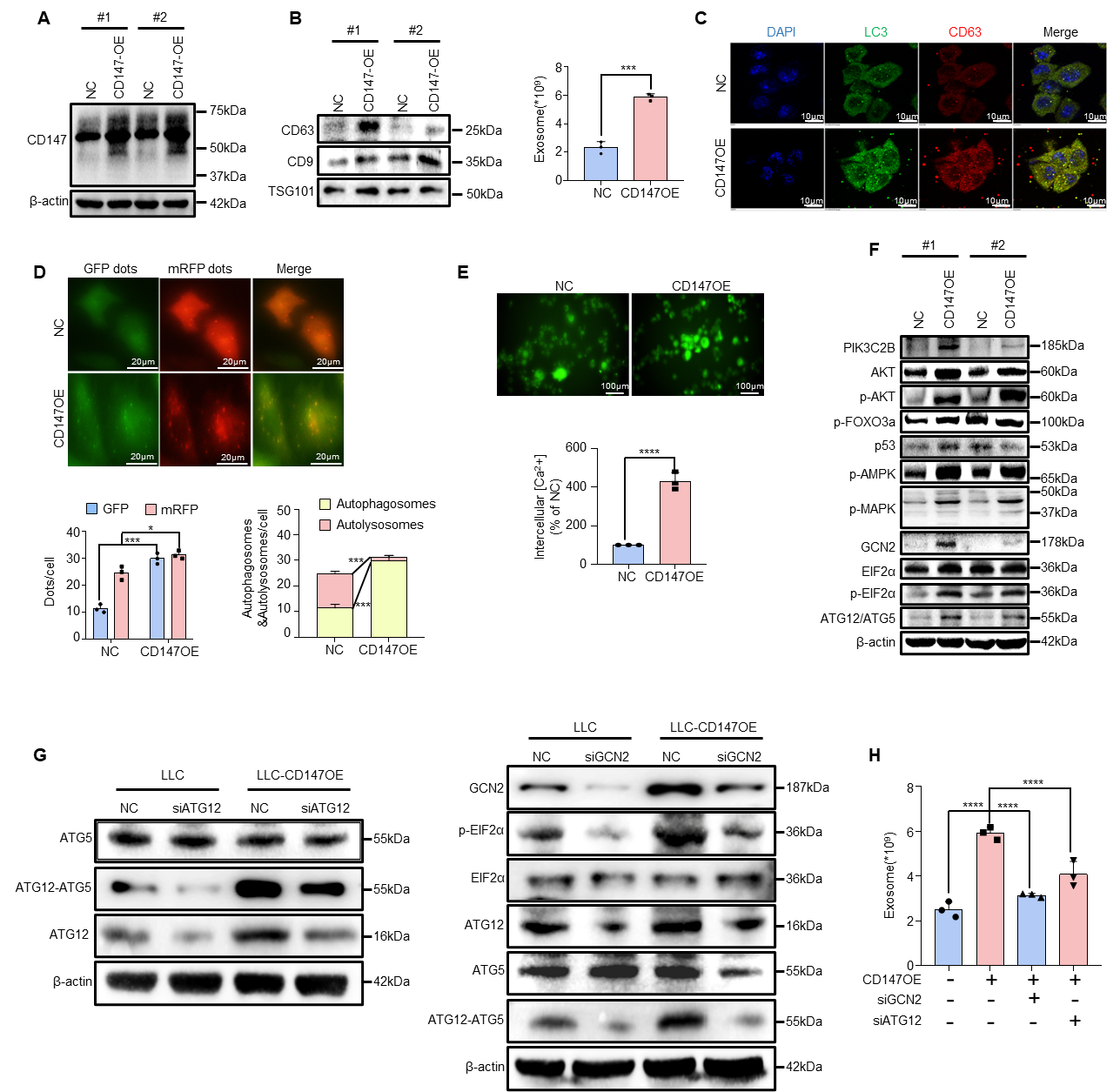
Extended Data Fig. 11 CD147 promotes exosomal secretion through calcium-mediated autophagic flux in Lewis lung carcinoma models.** **(A)** Immunoblot validation of CD147 expression in LLC cells subjected to CD147 overexpression (OE) versus empty vector control (EV). β-actin served as loading control. **(B)** Exosomal biogenesis characterization: Left: Immunoblot profiling of exosomal markers (CD9/CD63/TSG101) in LLC-derived exosomes under CD147 modulation; Right: Exosomal secretion quantified by Exo-CET assay normalized to 20 μg exosomal protein. **(C)** Super-resolution confocal microscopy demonstrating CD63 (Alexa Fluor™ 555) and LC3 (Alexa Fluor™ 488) colocalization in CD147-enriched membrane domains. **(D)** Autophagic flux analysis via RFP-GFP-LC3 lentiviral reporters: Representative images of autophagic compartments (yellow: autophagosomes; red: autolysosomes) (upper); Quantitative analysis of puncta (lower). **(E)** Intracellular calcium imaging: Left: Fluo-4 AM (5 μM) pseudocolor visualization of calcium dynamics; Right: ImageJ-based fluorescence intensity quantification. **(F)** Immunoblot analysis of autophagy-related proteins in LLC-NC vs. LLC-CD147OE cells. **(G)** GCN2/EIF2α/ATG12 pathway activation assessed by immunoblotting in CD147-modulated LLC models. **(H)** Exosomal output quantification showing CD147 dose-dependent secretion (ExoCET assay, 20 μg protein normalization).

|  | NSCLC patients (n=40) |
| --- | --- |
| Age (years) |  |
| ≥65 years | 18 |
| <65 years | 22 |
| Gender |  |
| Male | 23 |
| Female | 17 |
| Tumor stage |  |
| I/II/III/IV | 14/10/5/11 |
| Smoking history |  |
| Smoking | 21 |
| No-smoking | 19 |
| Primary tumor (T) /size (cm) |  |
| ≥3 cm | 15 |
| <3 cm | 25 |
| Pathological pattern |  |
| Adenocarcinoma | 28 |
| Squamous cell carcinoma | 12 |
| Lesion site |  |
| Left | 23 |
| Right | 17 |

**Supplementary Table 1. Clinical information of 40 NSCLC patients**

**Supplementary Table 2. Antibodies Information**

| Antibodies | Vendor | Catalogue No. | Dilution |
| --- | --- | --- | --- |
| AKT | Abcam | ab8805 | 1:1000 |
| ATG5 | CST | 12994 | 1:1000 |
| ATG7 | CST | D12B11 | 1:1000 |
| ATG12/ATG5 | CST | D88H1 | 1:1000 |
| Beclin-1 | Proteintech | 11306-1-AP | 1:1000 |
| BrdU | CST | 5292S | 1:1000 |
| CD147 | Produced by our lab | ­ | 1:1000  Exo: 1:500 |
| CD9 | Proteintech | 60232-1-Ig | 1:500(IF) |
| CD9 | Absin | abs136085 | 1:1000 |
| CD63 | Proteintech | 67605-1-Ig | 1:500(IF) |
| CD63 | Absin | abs132700 | 1:1000 |
| E-cadherin | CST | 3195S | 1:1000 |
| EIF2S1 | Proteintech | 11170-1-AP | 1:2000 |
| GCN2(EIF2AK4) | Proteintech | 27944-1-AP | 1:1000 |
| GM130 | Proteintech | 11038-1-AP | 1:1000 |
| His*TRIM68 | Proteintech | 66005-1-Ig | 1:50000 |
| ICTH | Proteintech | 67757-1-Ig | 1:1000 |
| IP3R3 | Proteintech | 20729-1-AP | 1:500 |
| IP3R2 | Santa Cruz | sc-398434 | 1:500 |
| IP3R1 | CST | 8568s | 1:500 |
| LAMP | Proteintech | 7300-1-Ig | 1:500 |
| LC3 | CST | CST-12741S | WB: 1:1000  IF:1:500 |
| mTOR | Proteintech | 67778-1-Ig | 1:1000 |
| Myc-ubi | CST | 2276 | 1:1000 |
| NEDD4L | Proteintech | 67276-1-Ig | 1:3000 |
| P53 | CST | 9282S | 1:1000 |
| P62 | sigma | P0067 | 1:1000 |
| P-AKT | Abcam | ab38449 | 1:1000 |
| P-AMPK | CST | 5759S | 1:1000 |
| PCBP1 | Abcam | ab168377 | 1:1000 |
| P-EIF2S1(Ser51) | Proteintech | 28740-1-AP | 1:1000 |
| P-FOXO3a | HUABIO | ST49-01 | 1:1000 |
| PIK3C2B | Proteintech | 24788-1-AP | 1:1000 |
| P-MAPK | CST | 2325S | 1:1000 |
| SNAP29 | Proteintech | 12704-1-AP | 1:1000 |
| Syntaxin17 | Proteintech | 17815-1-AP | 1:1000 |
| TRIM56 | Proteintech | 25509-1-AP | 1:1000 |
| TSG101 | Absin | abs122785 | 1:1000 |
| VAMP8 | Proteintech | 15546-1-AP | 1:1000 |
| Vimentin | CST | 5741S | 1:1000 |
| β-actin | CST | 3700S | 1:1000 |

**Supplementary Table 3. The primers used in the present study**

| **Gene** | **Primer sequence (5’- 3’)** |
| --- | --- |
| H-CD147 | F: ACTCCTCACCTGCTCCTTGA  R: GCCTCCATGTTCAGGTTCTC |
| M-CD147 | F: TGGCAAGTATGTGGTGGTAT  R: GTGAGATGGTTTCCCGAGT |
| GCN2 (EIF2AK4) | F: CGATGGAGAGCATGCAAAGC  R: TTCCCATCCACGTTGGTCAG |
| STX17 | F: CAGCTGTTACCAGGGAGGTC  R: ACCTTTCCAGGTCTGTTGGG |
| VAMP8 | F: AATGATCGTGTGCGGAACCT  R: GTGCTCAGATGTGGCTTCCA |
| SNAP29 | F: CTGGCCCTCATGTACGAGTC  R: AGGGTGCCATTCTGTTCAGG |
| ATG5 | F: GCATCAAGTTCAGCTCTTCCTTGG  R: GATGTTCACTCAGCCACTGCAGAGG |
| ATG6 | F: ATCCTGGACCGTGTCACCATCCAGG  R: GTTGAGCTGAGTGTCCAGCTGG |
| ATG7 | F: CGGCGGATCCAATTCCTGTA  R: GGATGCACTGGATACCAGCA |
| LC3A | F: TTCCGAGTTGCTGACTGACC  R: CCCTTGTAGCGCTCGATGAT |
| LC3B | F: TTCAGGTTCACAAAACCCGC  R: TCTCACACAGCCCGTTTACC |
| ATG12 | F: AAGTGGGCAGTAGAGCGAAC  R: CACGCCTGAGACTTGCAGTA |
| β-actin | F: TCTACAATGAGCTGCGTGTGG  R: CTGGATAGCAACGTACATGGC |

| DESCRIPTION  **Supplementary Table 4. Original and mutated sequences** | FASTA | SEQUENCES | mutations |
| --- | --- | --- | --- |
| GCN2  (K610R) | Original:  AVIkVQNKLDGCCYA  Mutations:  AVIkVQNRLDGCCYA | GCTGTCATCAAGGTGCAGAACAAGTTGGACGGCTGCTGCTACGCA | GCTGTCATCAAGGTGCAGAACAGGTTGGACGGCTGCTGCTACGCA |
| GCN2  (K619R)) | Original:  DGCCYAVKRIPINPA  Mutations:  DGCCYAVRRIPINPA | GACGGCTGCTGCTACGCAGTGAAGCGCATCCCCATCAACCCGGCC | GACGGCTGCTGCTACGCAGTGAGGCGCATCCCCATCAACCCGGCC |
| GCN2  (K634R) | Original:  SRQFRRIKGEVTLLS  Mutations:  SRQFRRIRGEVTLLS | AGCCGGCAGTTCCGCAGGATCAAGGGCGAAGTGACACTGCTGTCA | AGCCGGCAGTTCCGCAGGATCAGGGGCGAAGTGACACTGCTGTCA |
| GCN2  (K1489R) | Original:  LVDHVLQKLRTKVTD  Mutations:  LVDHVLQRLRTKVTD | CTTGTGGACCATGTACTGCAGAAACTGAGGACTAAAGTCACTGAT | CTTGTGGACCATGTACTGCAGAGACTGAGGACTAAAGTCACTGAT |
|  |  |  |  |
|  |  |  |  |
