# Supplementary material for: CD147 promotes NSCLC metastasis by inducing secretory autophagy-dependent exosome secretion via TRIM56-mediated ubiquitination and degradation of GCN2
Source: Cell Death Differ. 2025 Dec 18;33(6):1152–74. doi: 10.1038/s41418-025-01636-y (PMC13247162; doi:10.1038/s41418-025-01636-y)

Fig.1G

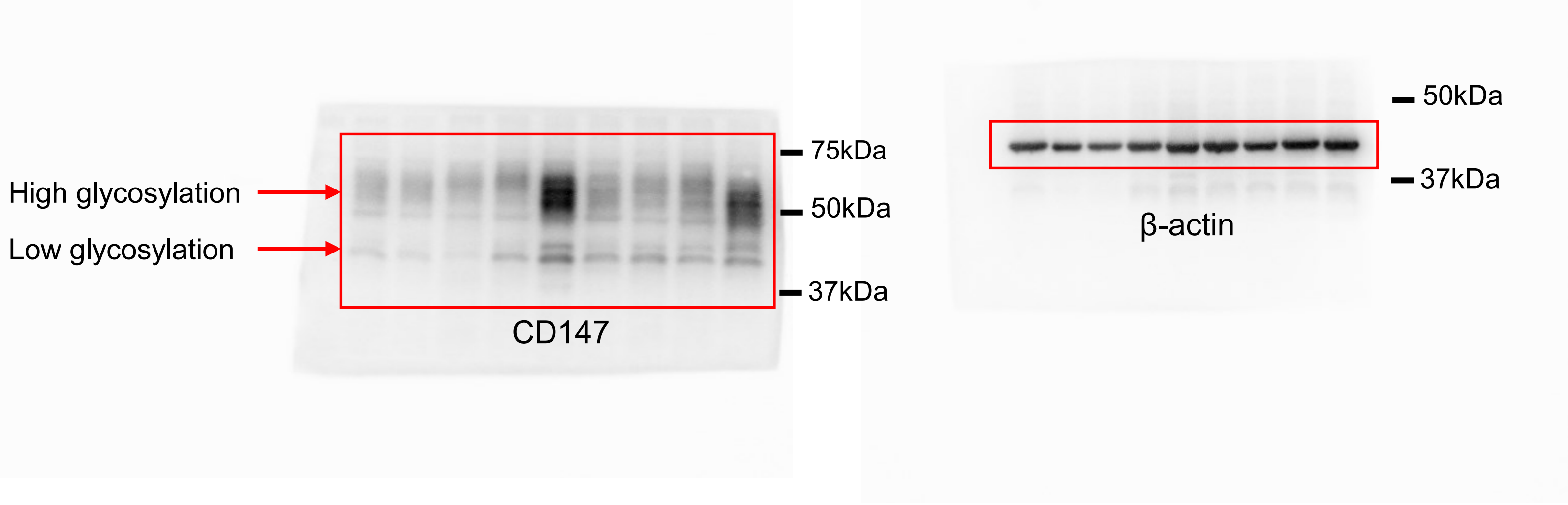
High glycosylation
Low glycosylation
75kDa
50kDa
37kDa
CD147
50kDa
37kDa
β-actin

Fig.2A, B

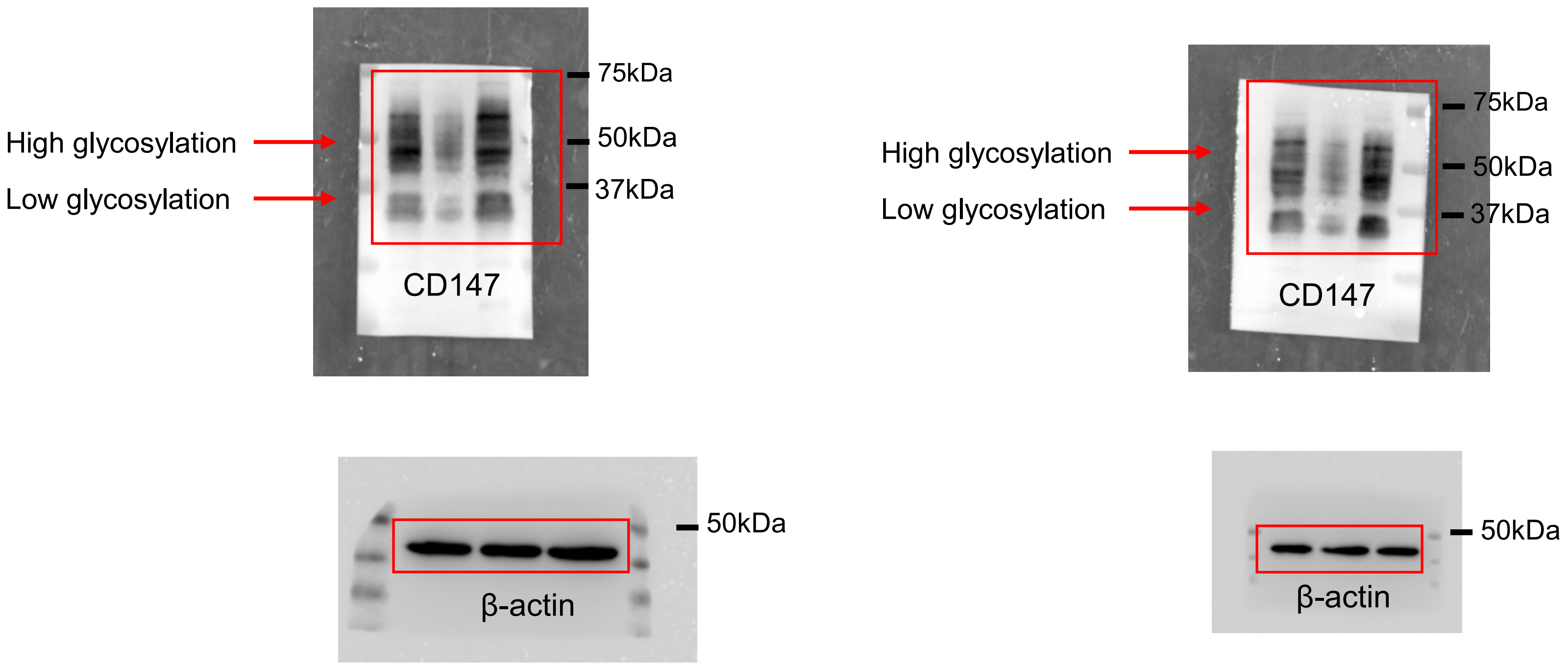

Fig.2C, D

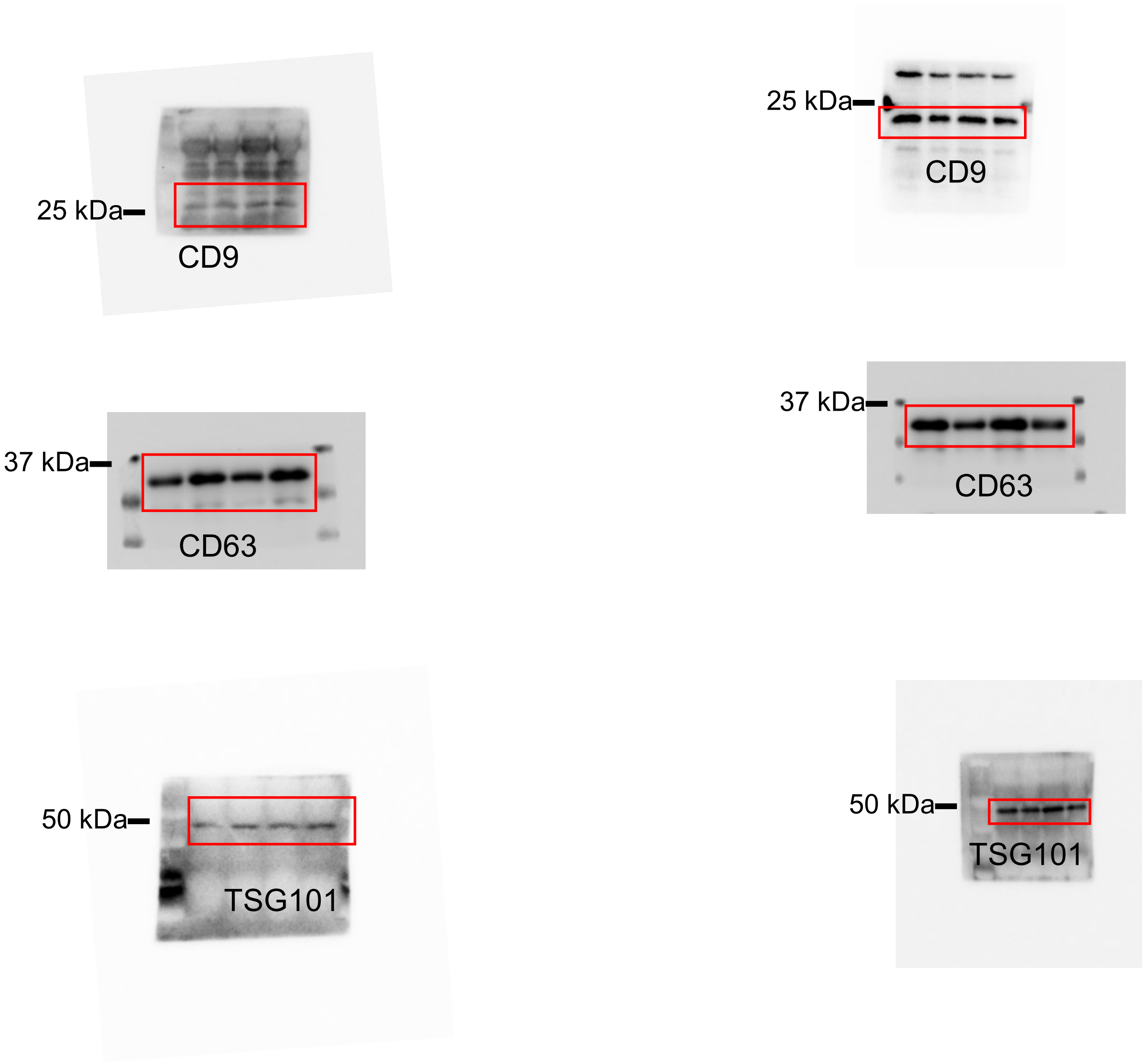

Fig.3A

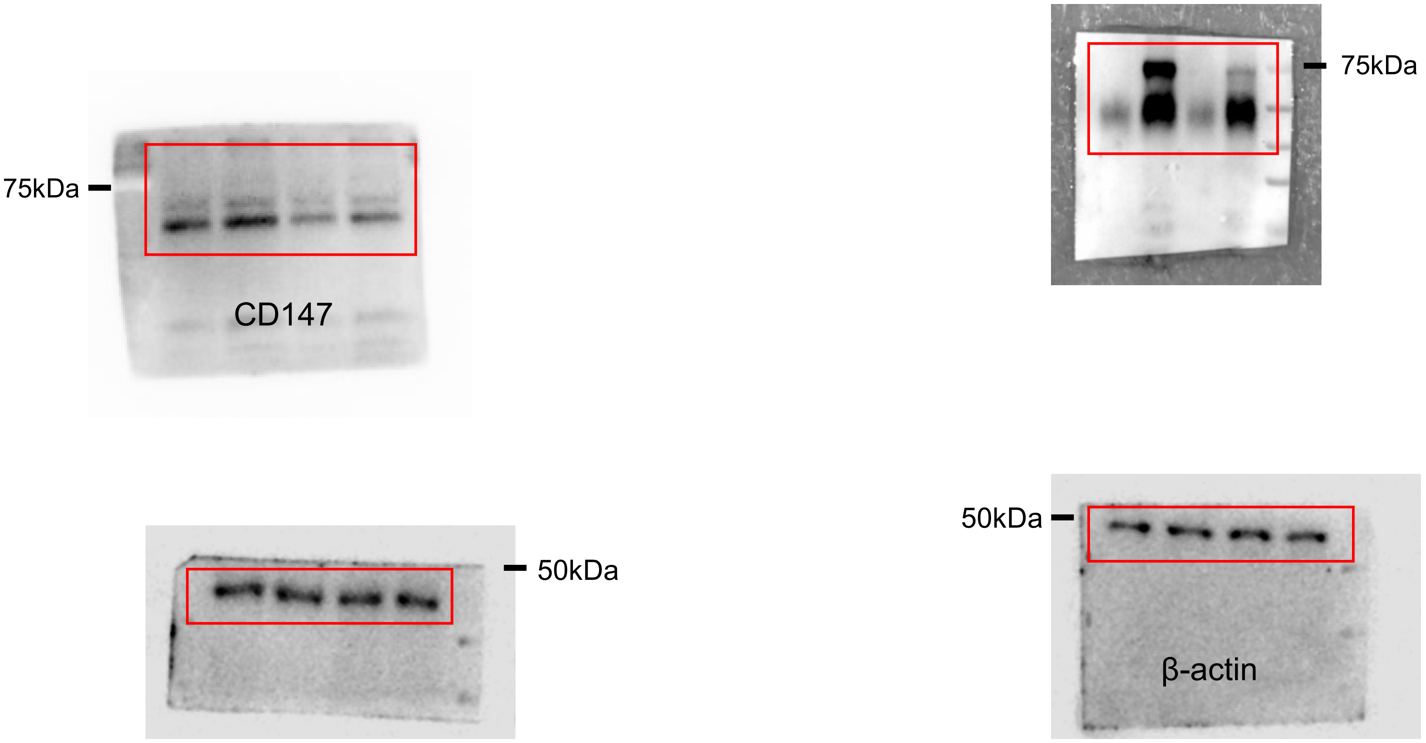

Fig.4D

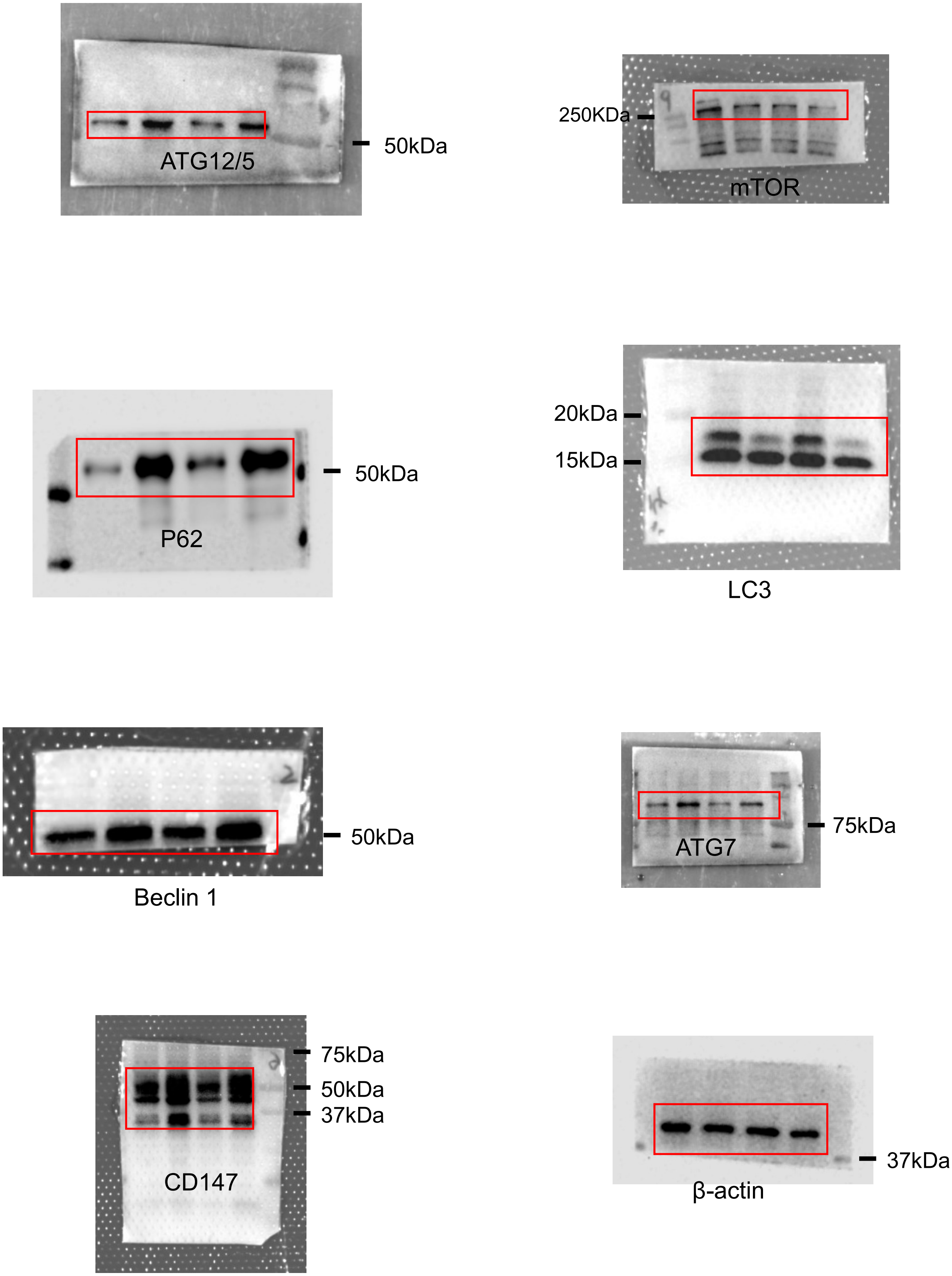
ATG12/5
50kDa
250KDa
mTOR
P62
50kDa
20kDa
15kDa
LC3
50kDa
Beclin 1
75kDa
ATG7
75kDa
50kDa
37kDa
CD147
37kDa
β-actin

Fig.6B-A549

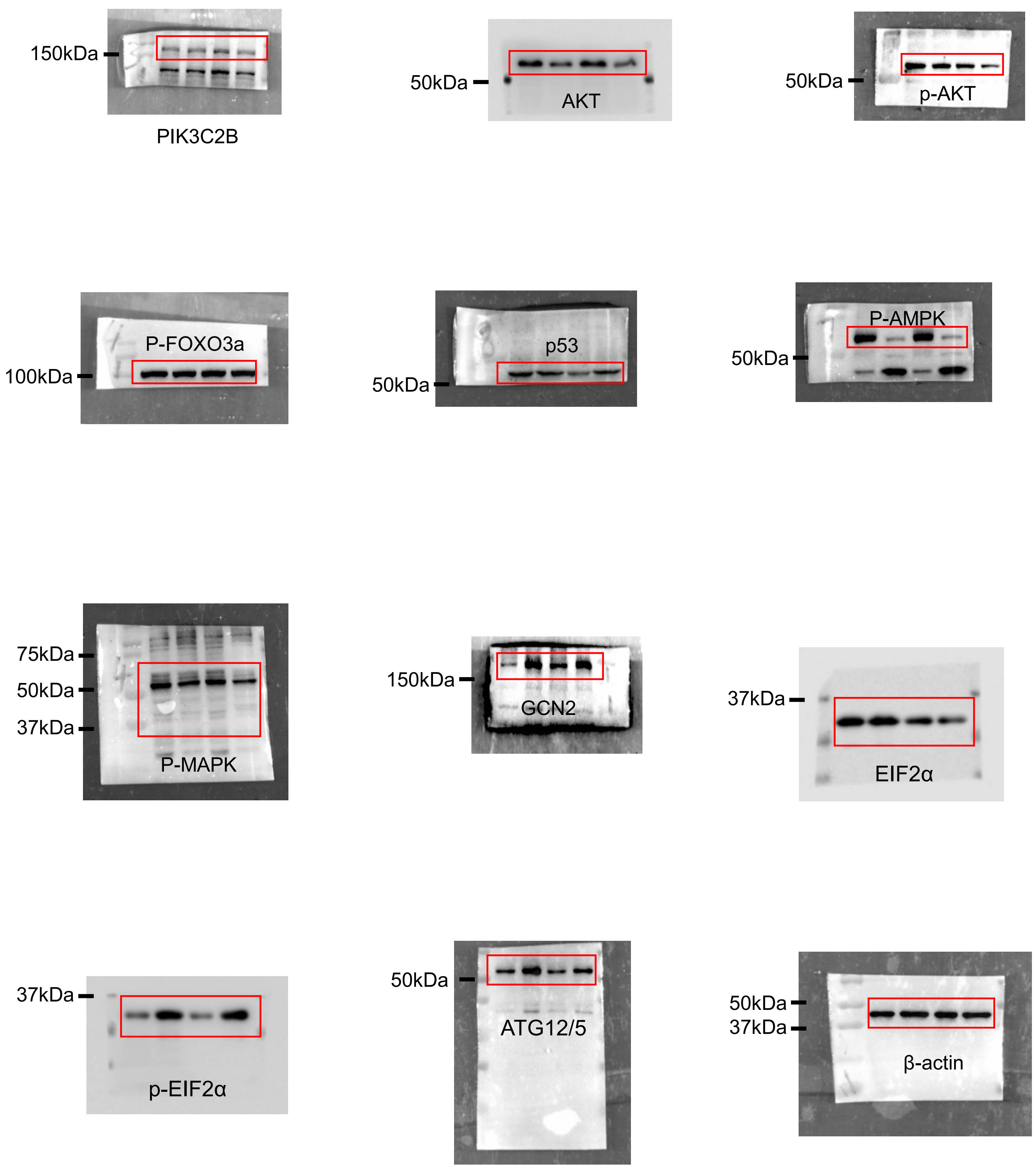
150kDa
PIK3C2B
50kDa
AKT
50kDa
p-AKT
100kDa
P-FOXO3a
50kDa
p53
50kDa
P-AMPK
75kDa
50kDa
37kDa
P-MAPK
150kDa
GCN2
37kDa
EIF2α
37kDa
p-EIF2α
50kDa
ATG12/5
50kDa
37kDa
β-actin

Fig.6B-H460

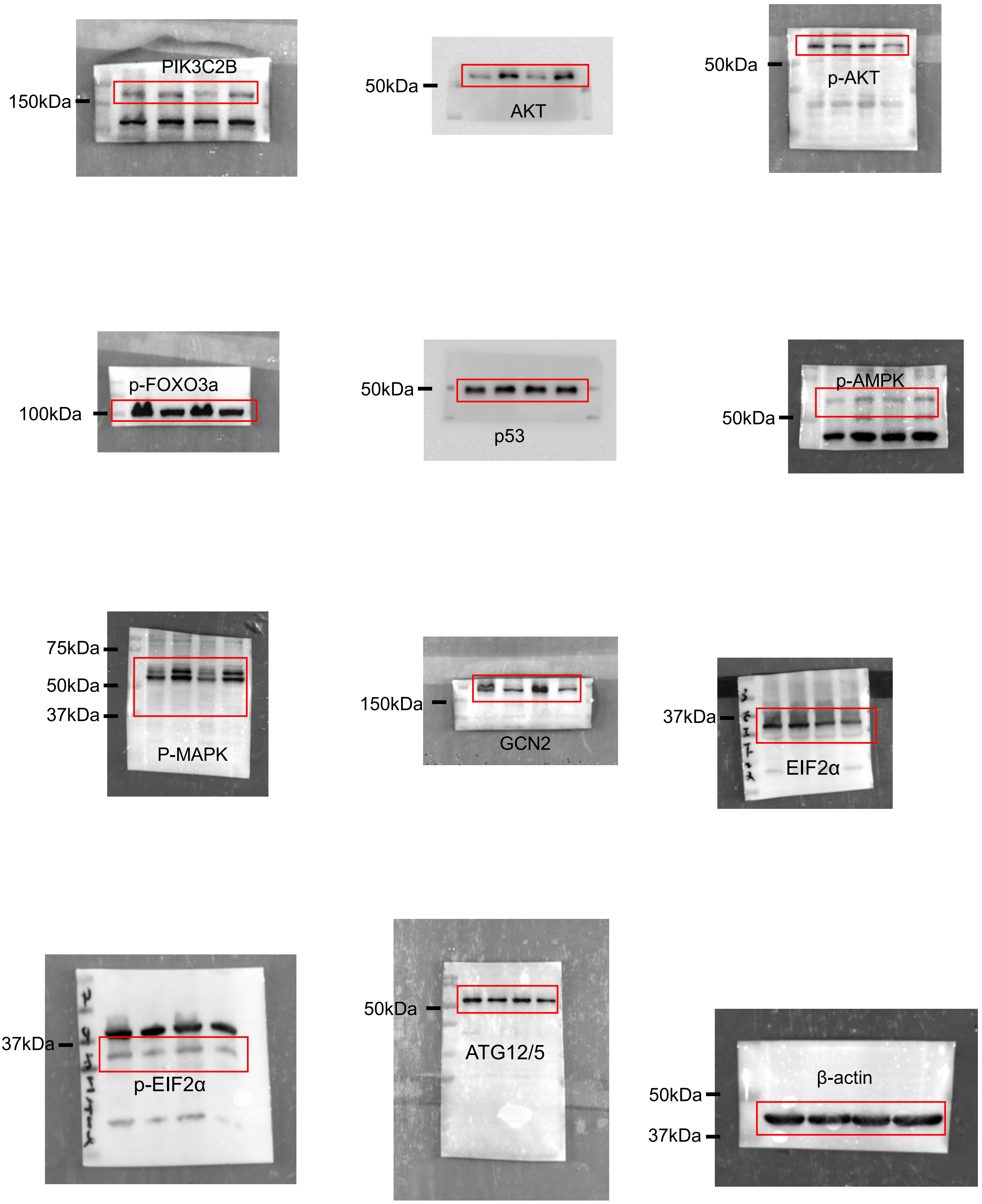
PIK3C2B
150kDa
AKT
50kDa
p-AKT
50kDa
p-FOXO3a
100kDa
p53
50kDa
p-AMPK
50kDa
P-MAPK
75kDa
50kDa
37kDa
GCN2
150kDa
EIF2α
37kDa
p-EIF2α
37kDa
ATG12/5
50kDa
β-actin
50kDa
37kDa

Fig.6E, G

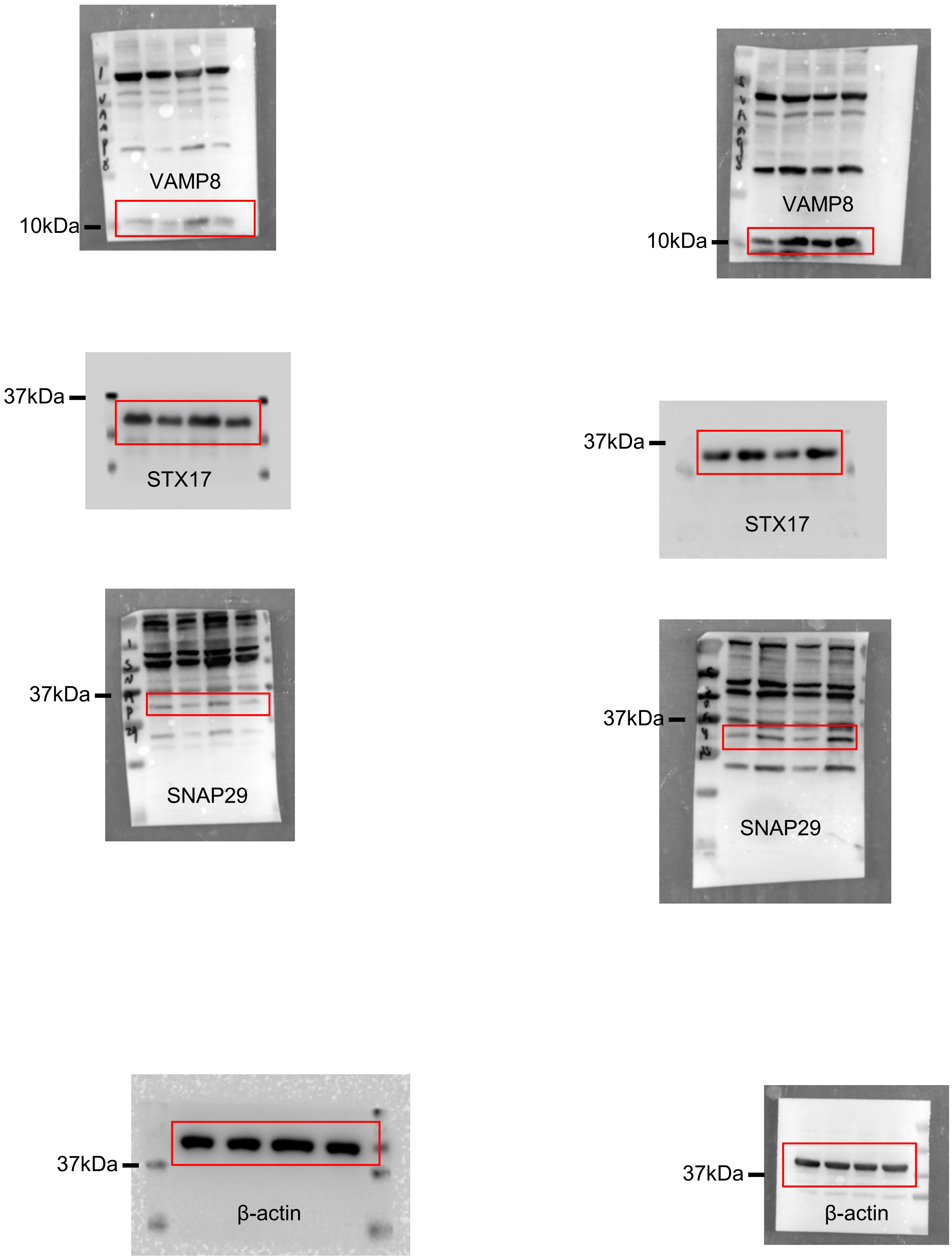
VAMP8
10kDa
VAMP8
10kDa
37kDa
STX17
37kDa
STX17
37kDa
SNAP29
37kDa
SNAP29
37kDa
β-actin
37kDa
β-actin

Fig.7B

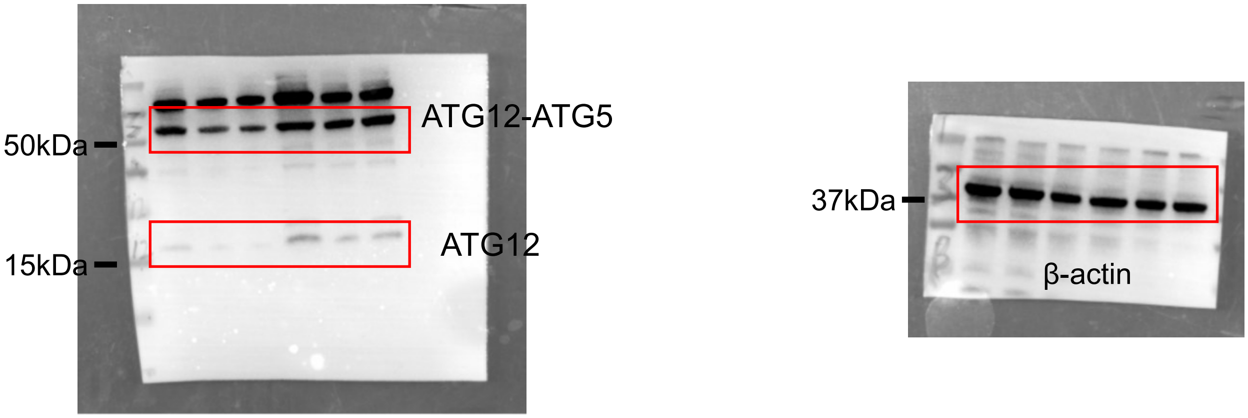

ATG12-ATG5
50kDa
ATG12
15kDa
37kDa
β-actin

Fig.7C

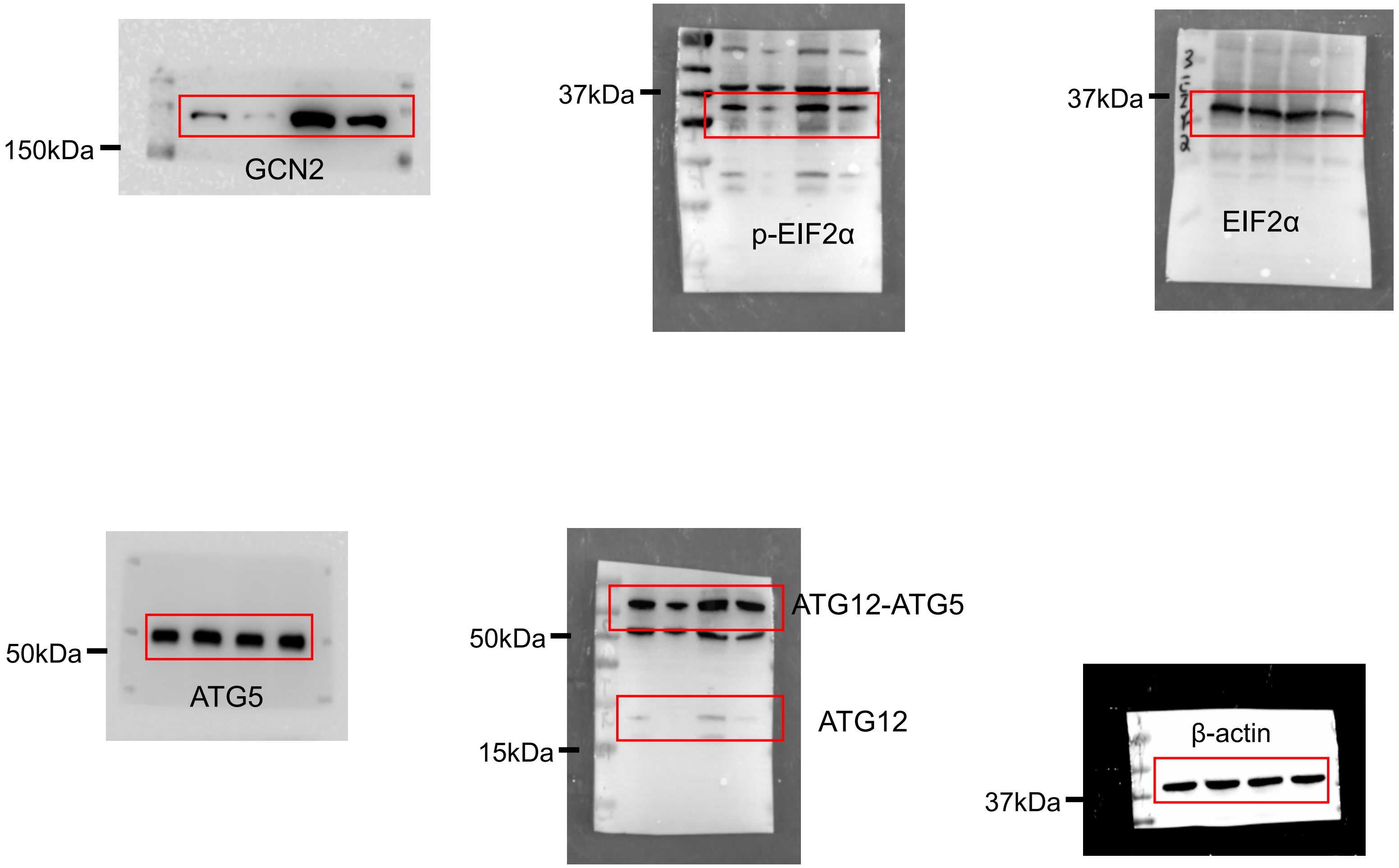

150kDa
GCN2
37kDa
p-EIF2α
37kDa
EIF2α
50kDa
ATG5
ATG12-ATG5
50kDa
ATG12
15kDa
β-actin
37kDa

Fig.8B

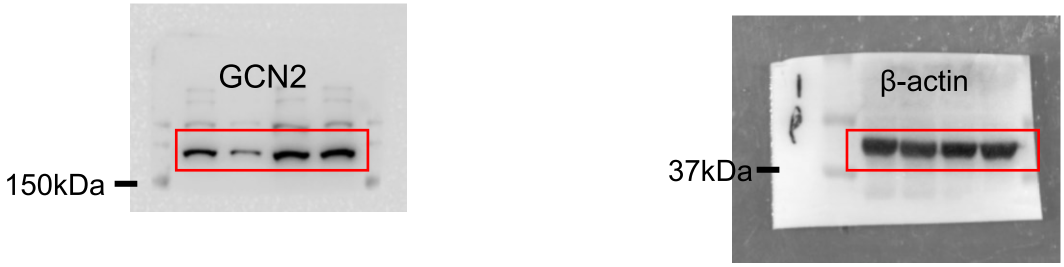
GCN2
150kDa
β-actin
37kDa

Fig.8D

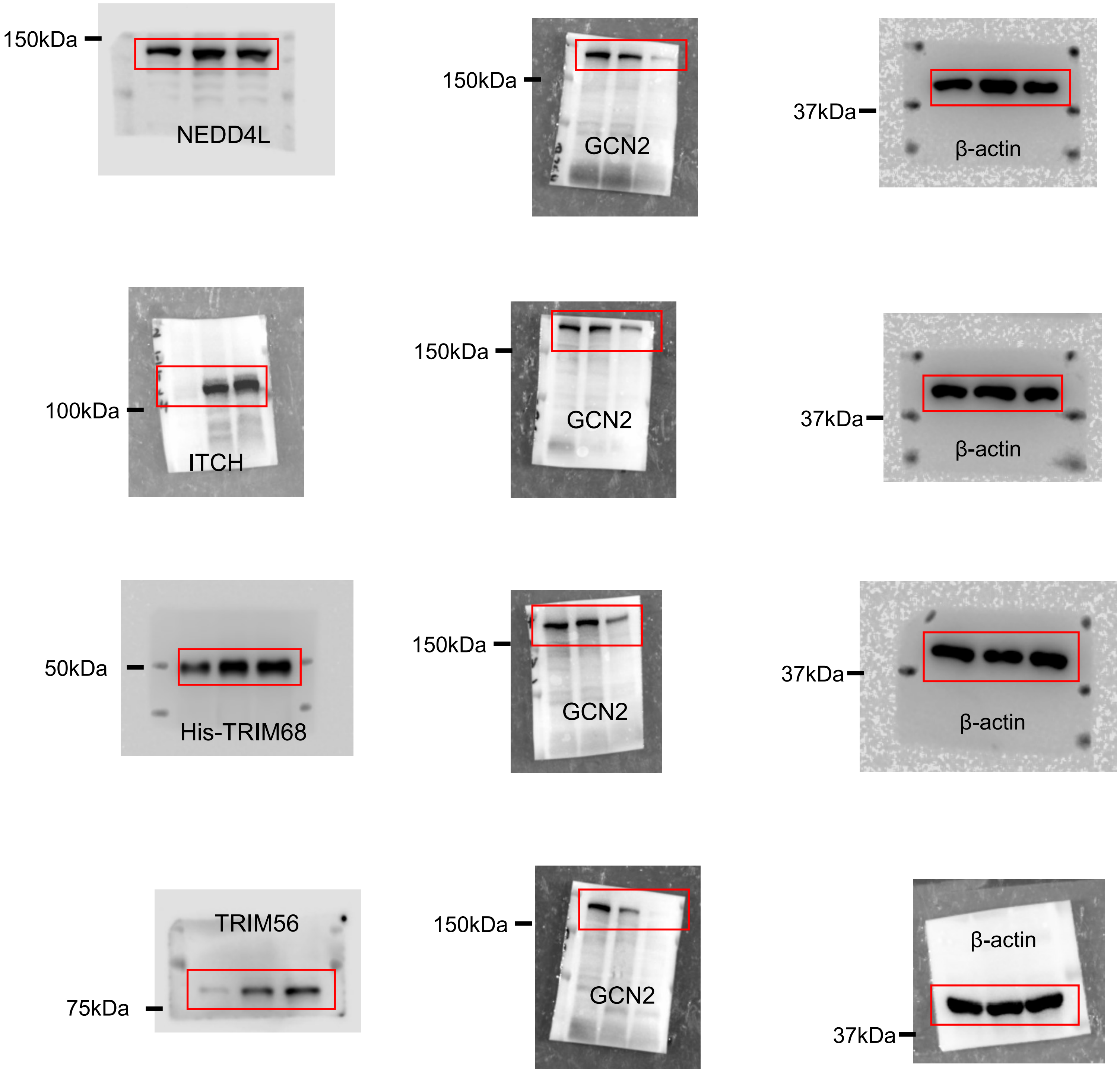
150kDa
NEDD4L
150kDa
GCN2
37kDa
β-actin
100kDa
ITCH
150kDa
GCN2
37kDa
β-actin
50kDa
His-TRIM68
150kDa
GCN2
37kDa
β-actin
TRIM56
75kDa
150kDa
GCN2
β-actin
37kDa

Fig.8E

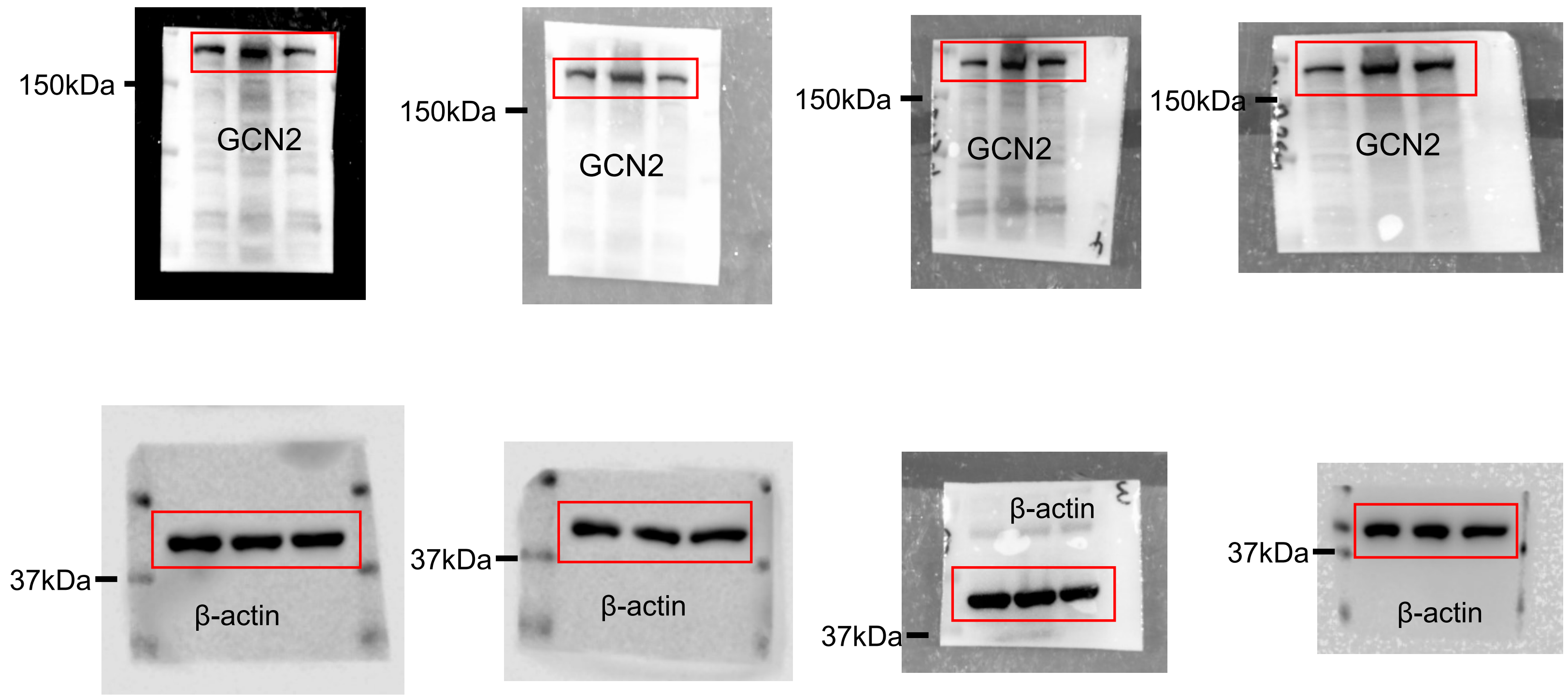
150kDa
GCN2
150kDa
GCN2
150kDa
GCN2
150kDa
GCN2
37kDa
β-actin
37kDa
β-actin
β-actin
37kDa
37kDa
β-actin

Fig.8F

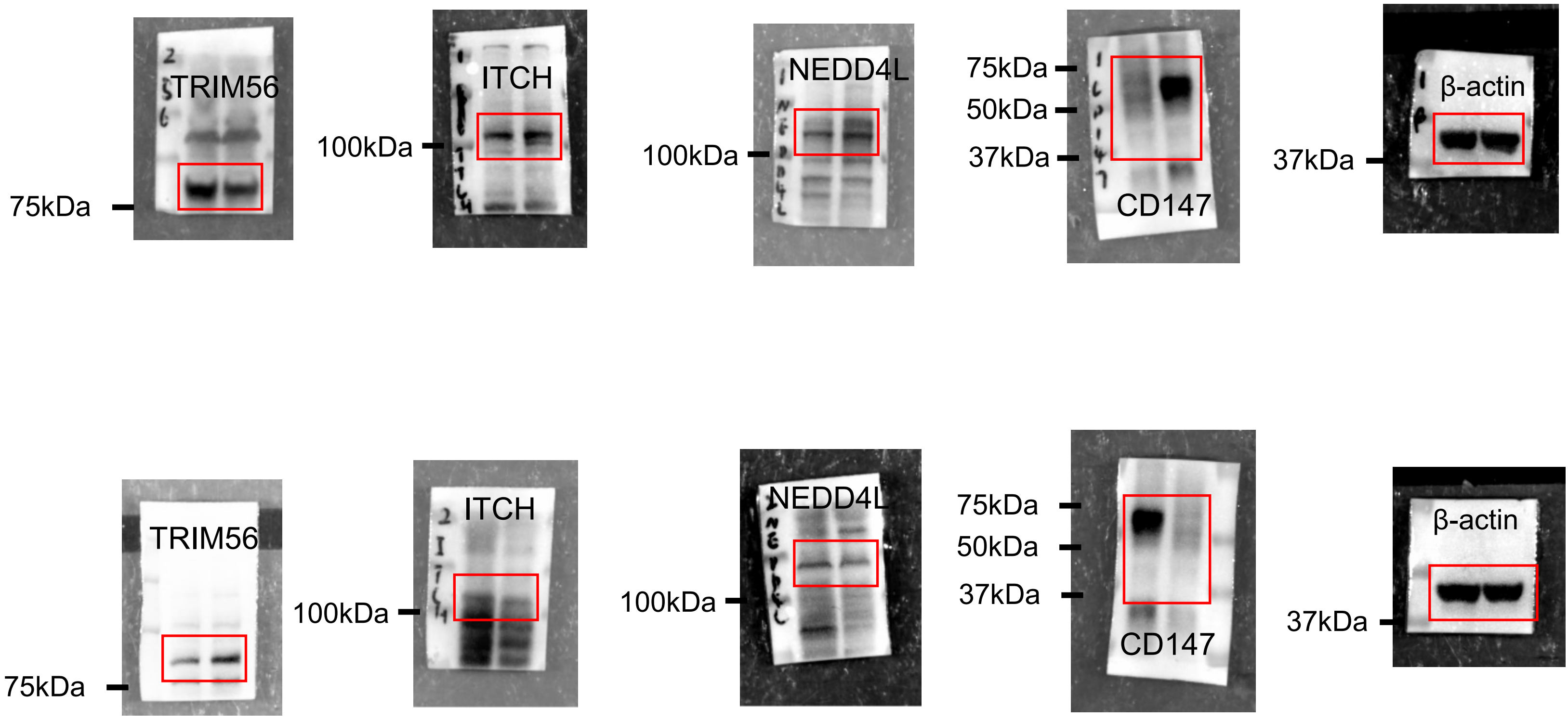
TRIM56
75kDa
ITCH
100kDa
NEDD4L
100kDa
75kDa
50kDa
37kDa
CD147
β-actin
37kDa
TRIM56
75kDa
ITCH
100kDa
NEDD4L
100kDa
75kDa
50kDa
37kDa
CD147
β-actin
37kDa

Fig.8G

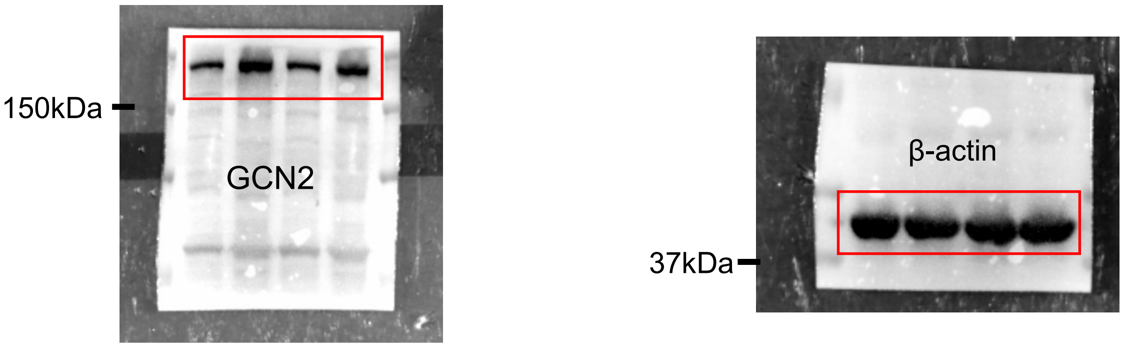

Fig.8H

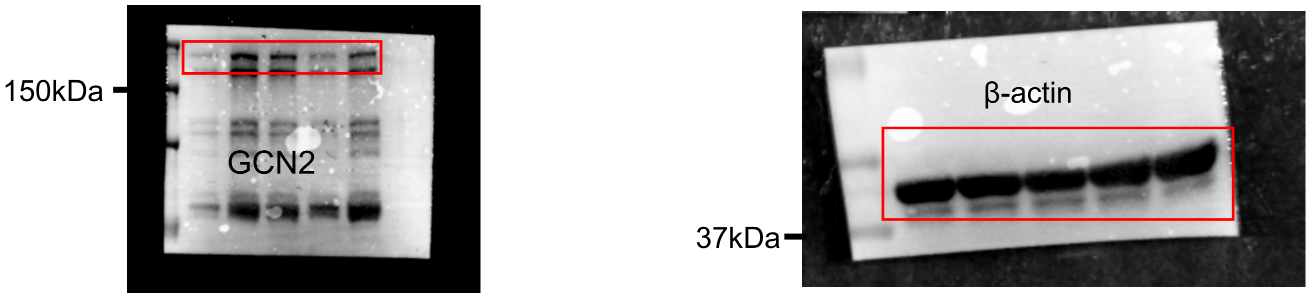

Fig.8I

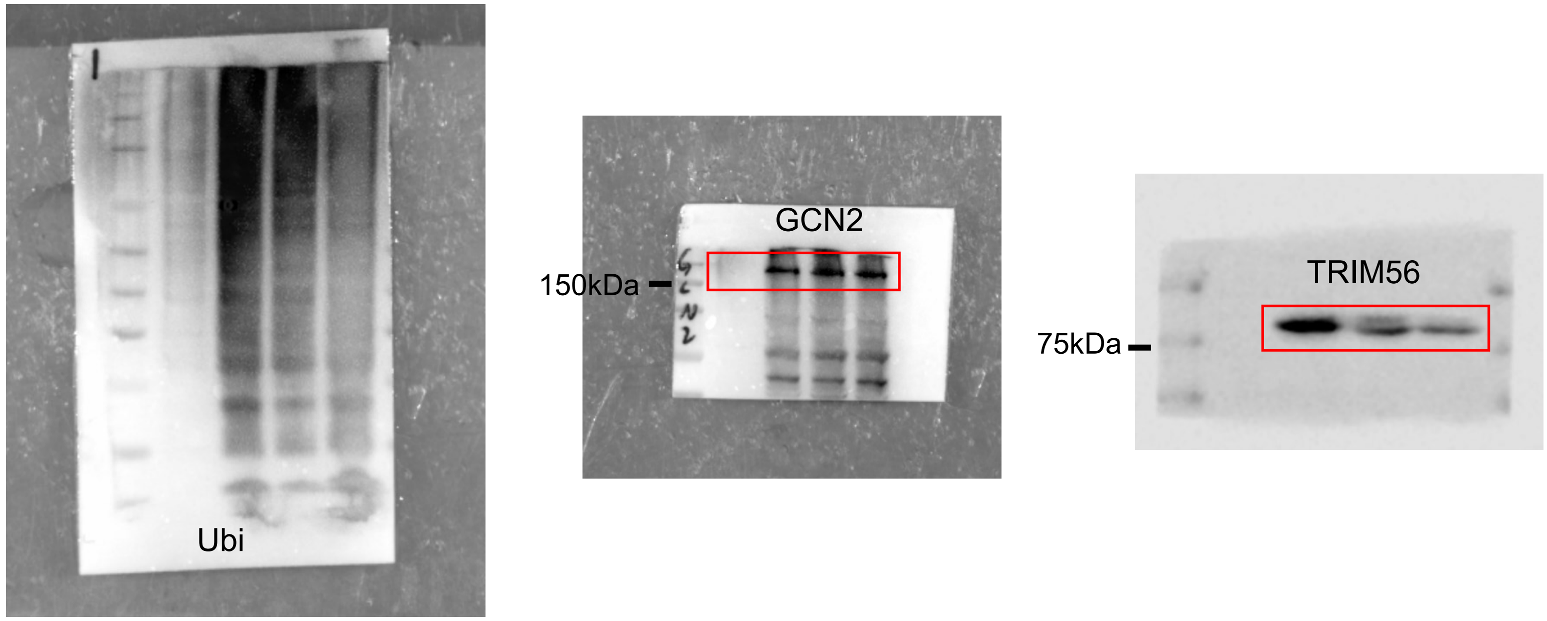

Fig.8J

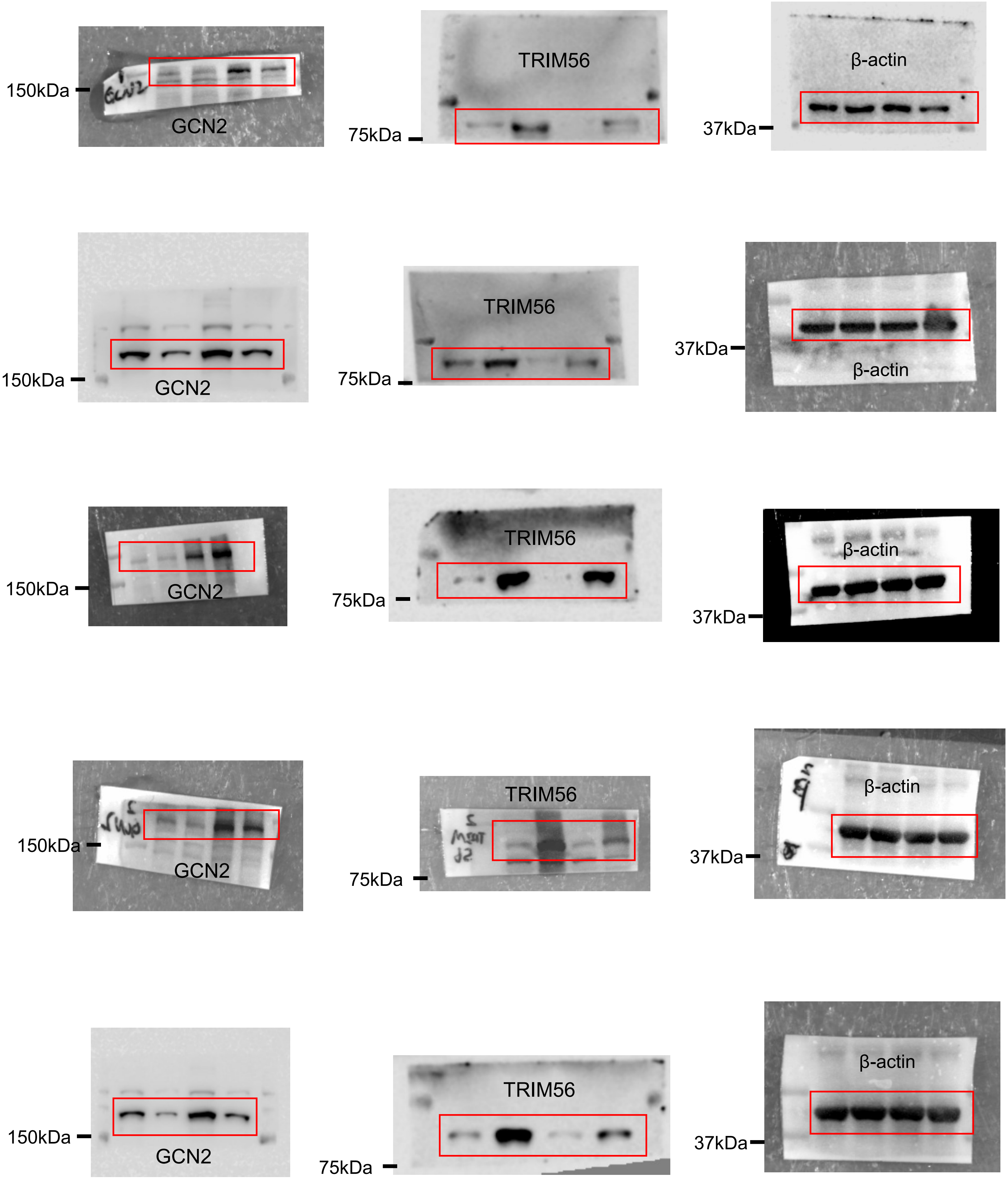
150kDa
GCN2
TRIM56
75kDa
β-actin
37kDa
150kDa
GCN2
TRIM56
75kDa
37kDa
β-actin
150kDa
GCN2
TRIM56
75kDa
β-actin
37kDa
150kDa
GCN2
TRIM56
75kDa
β-actin
37kDa
150kDa
GCN2
TRIM56
75kDa
β-actin
37kDa

Fig.9A

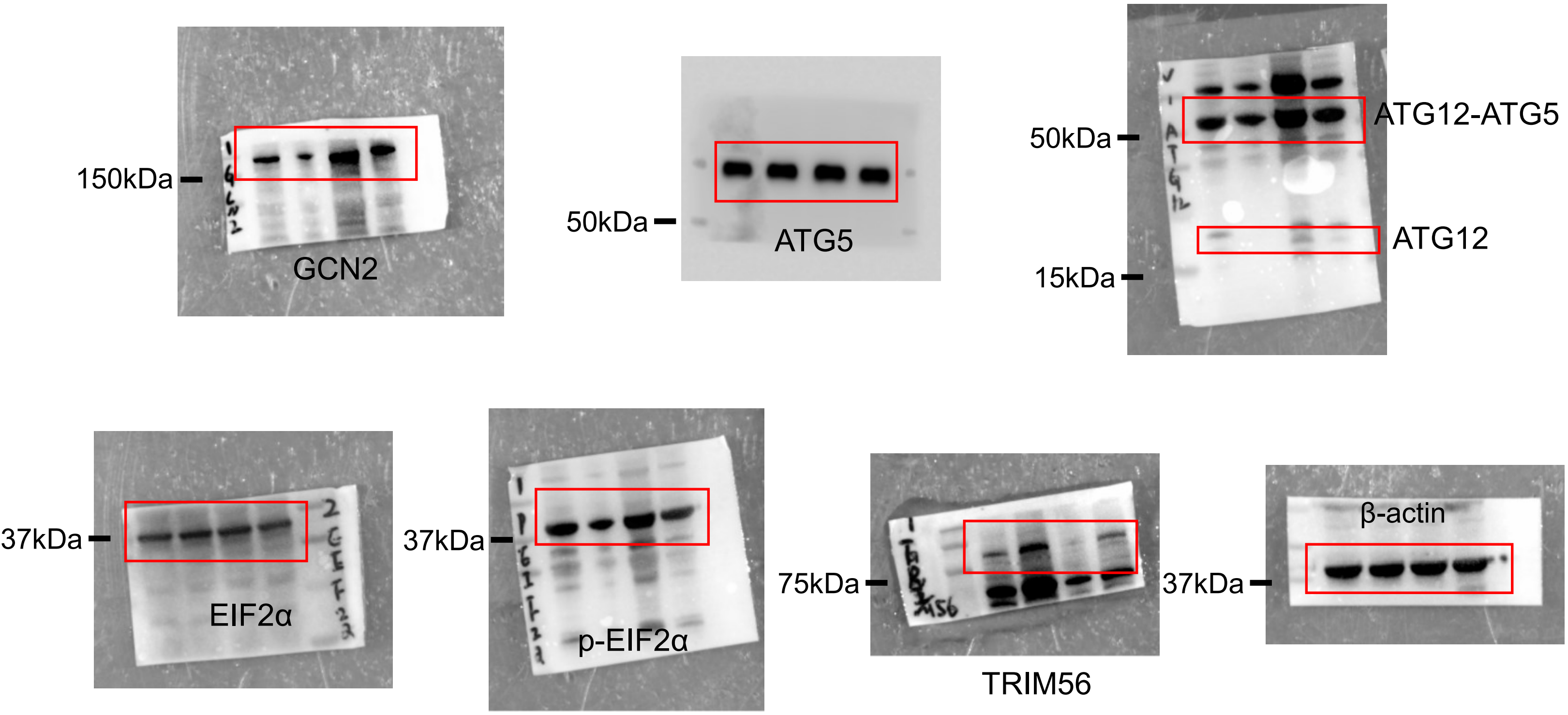
150kDa
GCN2
50kDa
ATG5
50kDa
ATG12-ATG5
ATG12
15kDa
37kDa
EIF2α
37kDa
p-EIF2α
75kDa
TRIM56
37kDa
β-actin

Fig.9E

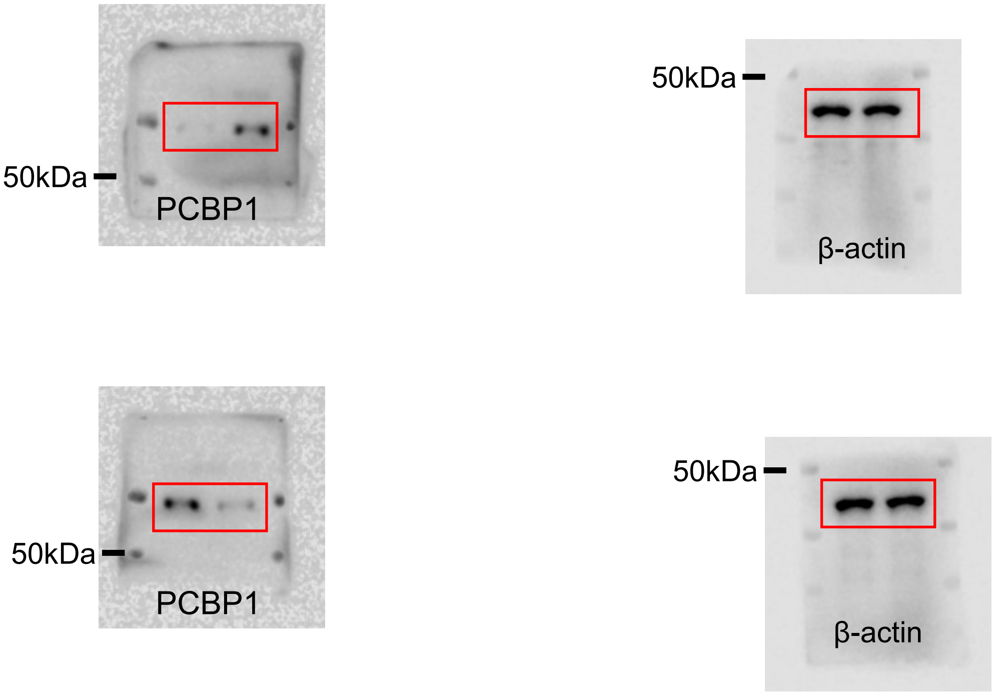
50kDa
PCBP1
50kDa
β-actin
50kDa
PCBP1
50kDa
β-actin

Fig.9F

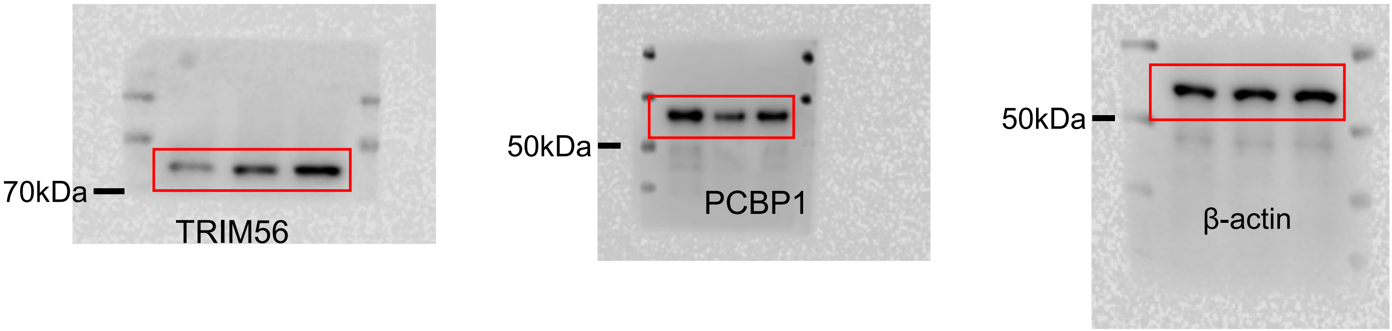
70kDa
TRIM56
50kDa
PCBP1
50kDa
β-actin

Fig.9G

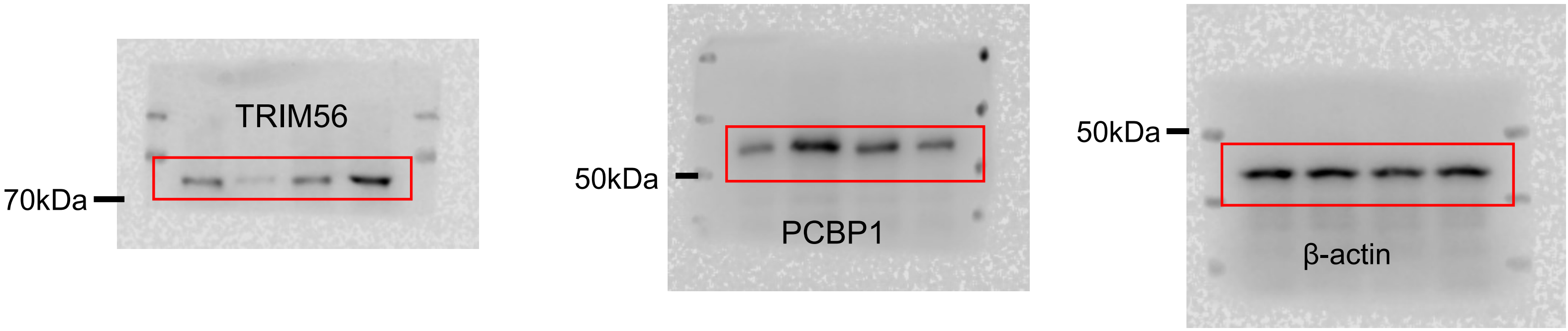
TRIM56
70kDa
50kDa
PCBP1
50kDa
β-actin

Fig.10E

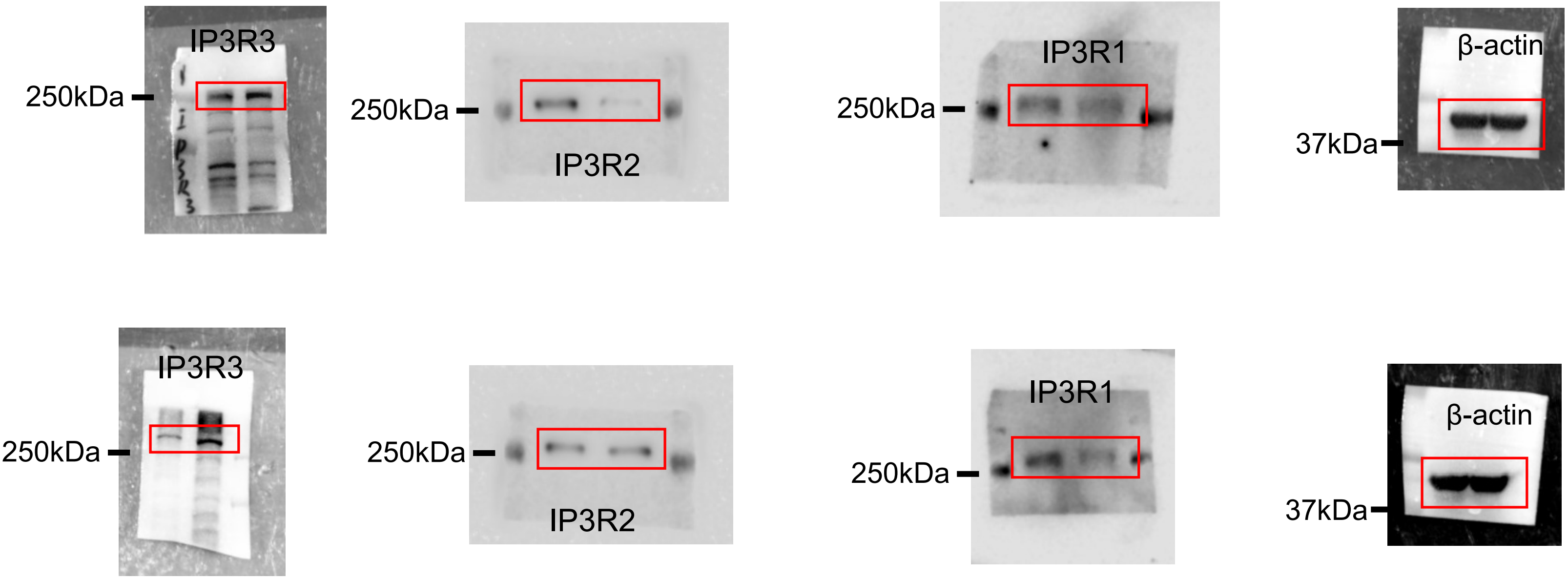

Fig.10F

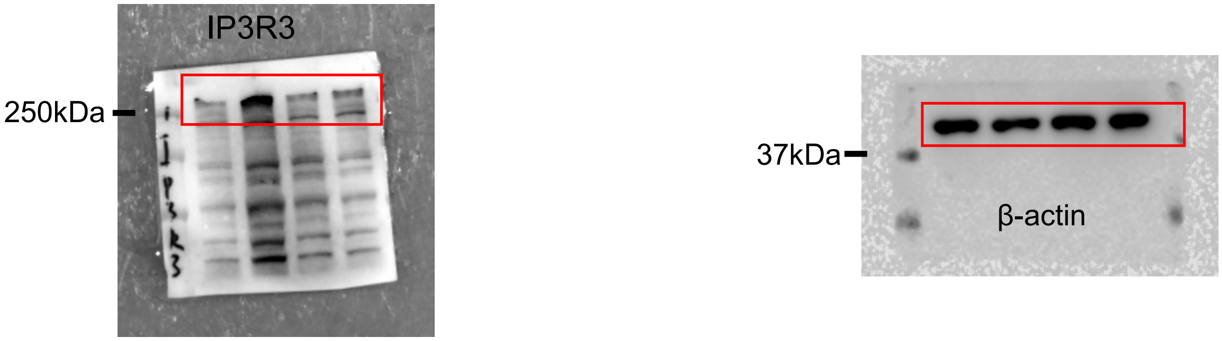

Extended Data Fig.1H

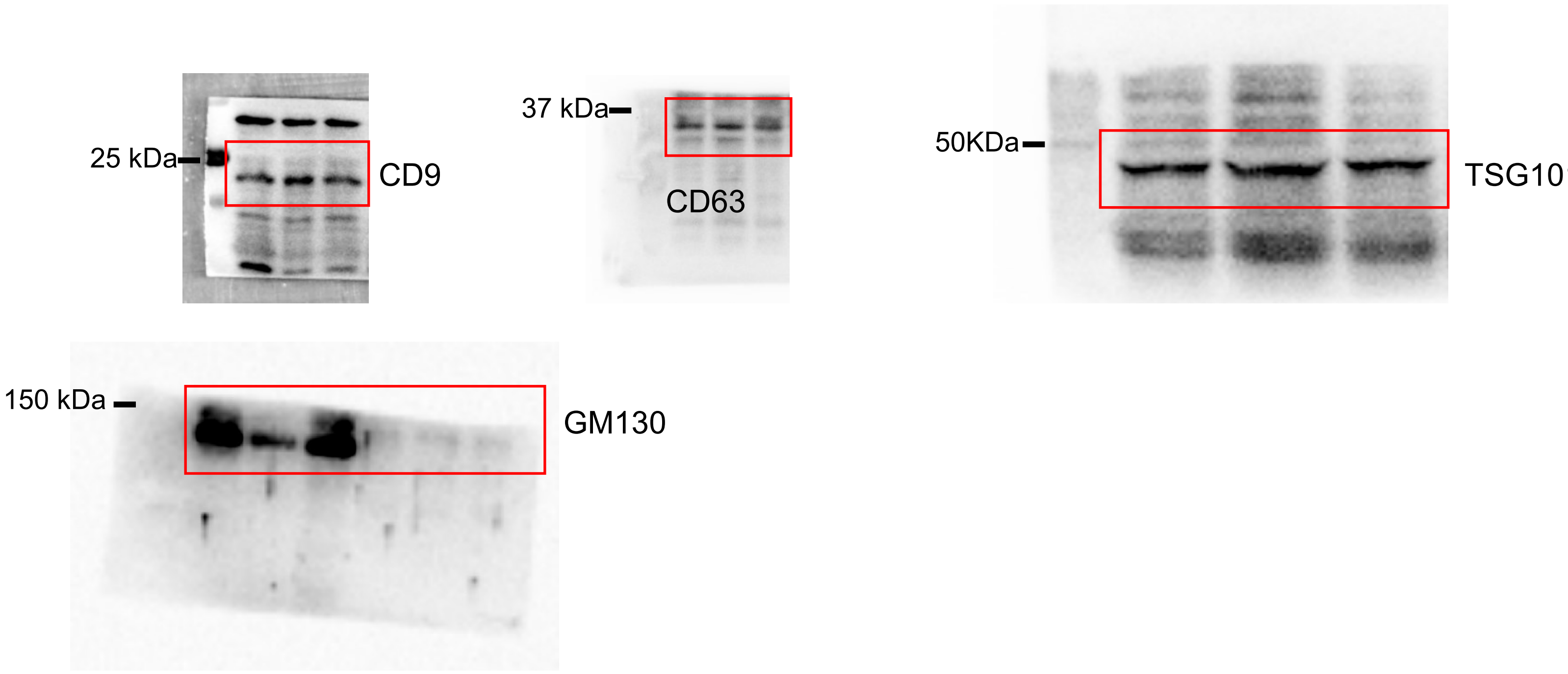

Extended Data Fig. 1L

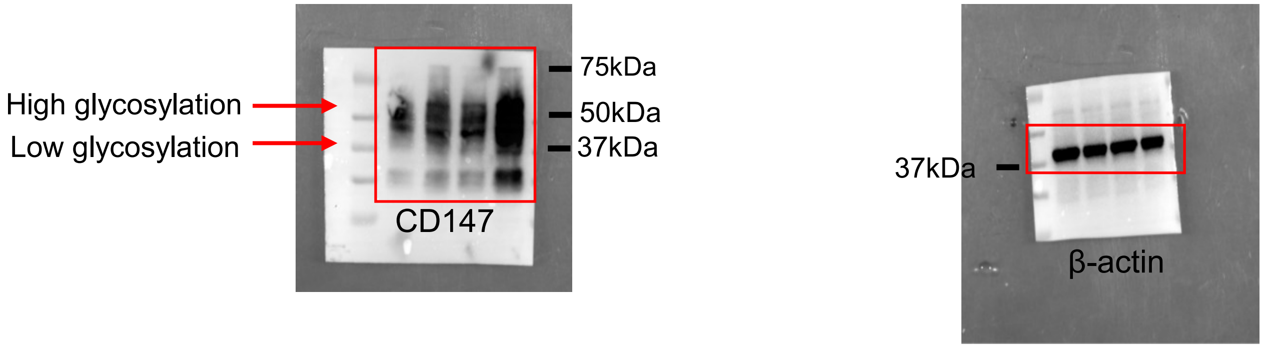

Extended Data Fig. 2A,B

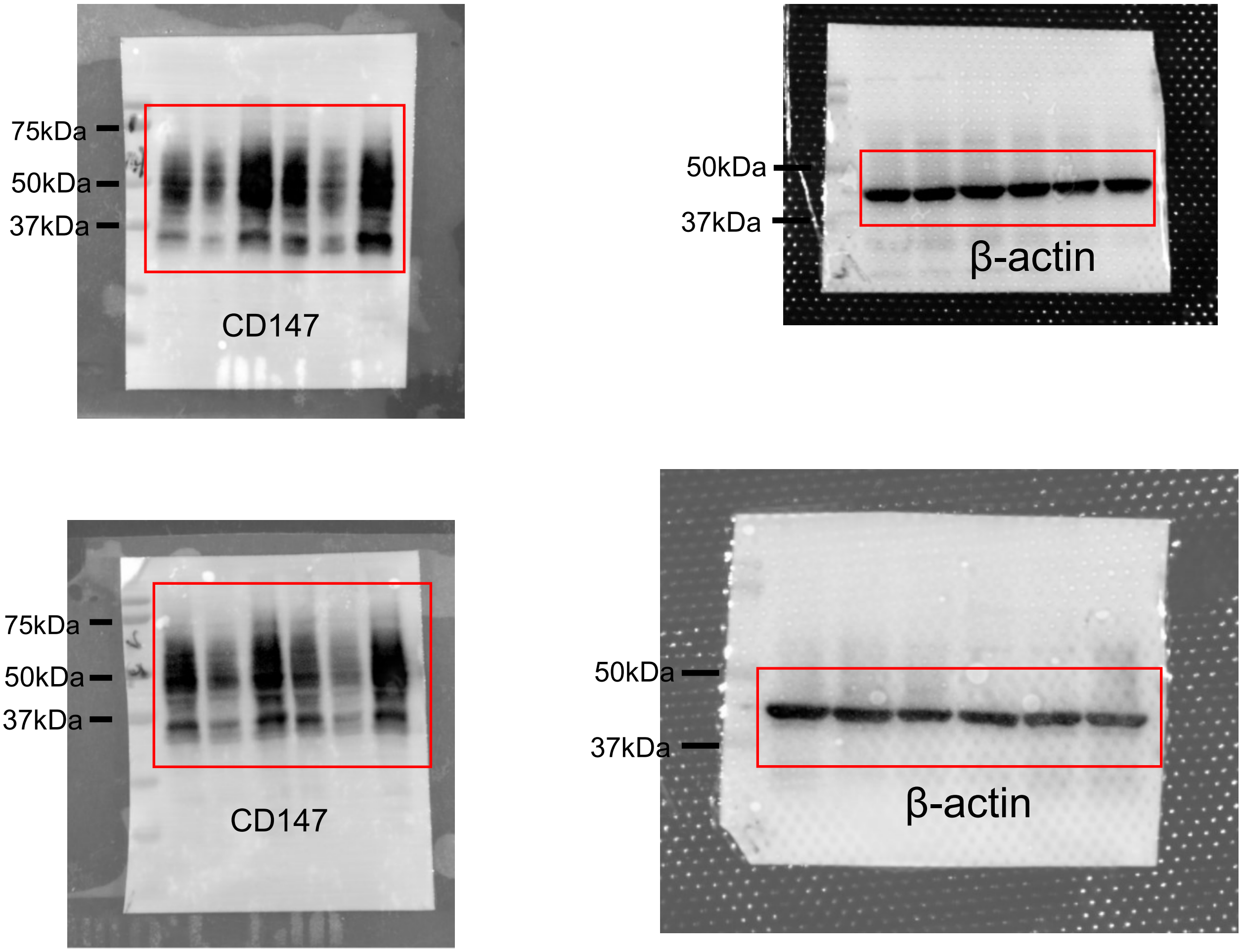

Extended Data Fig. 3L

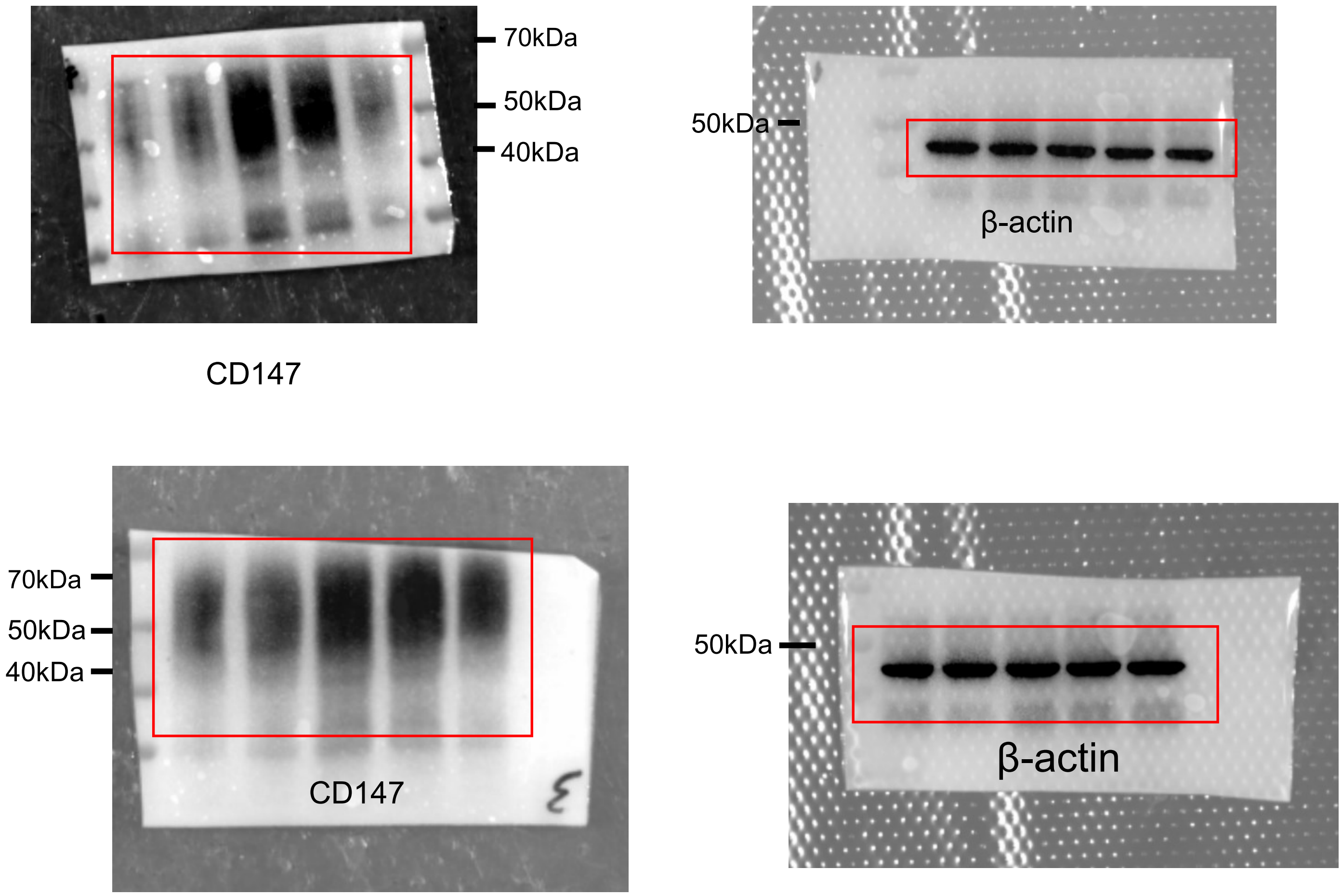

Extended Data Fig. 3M

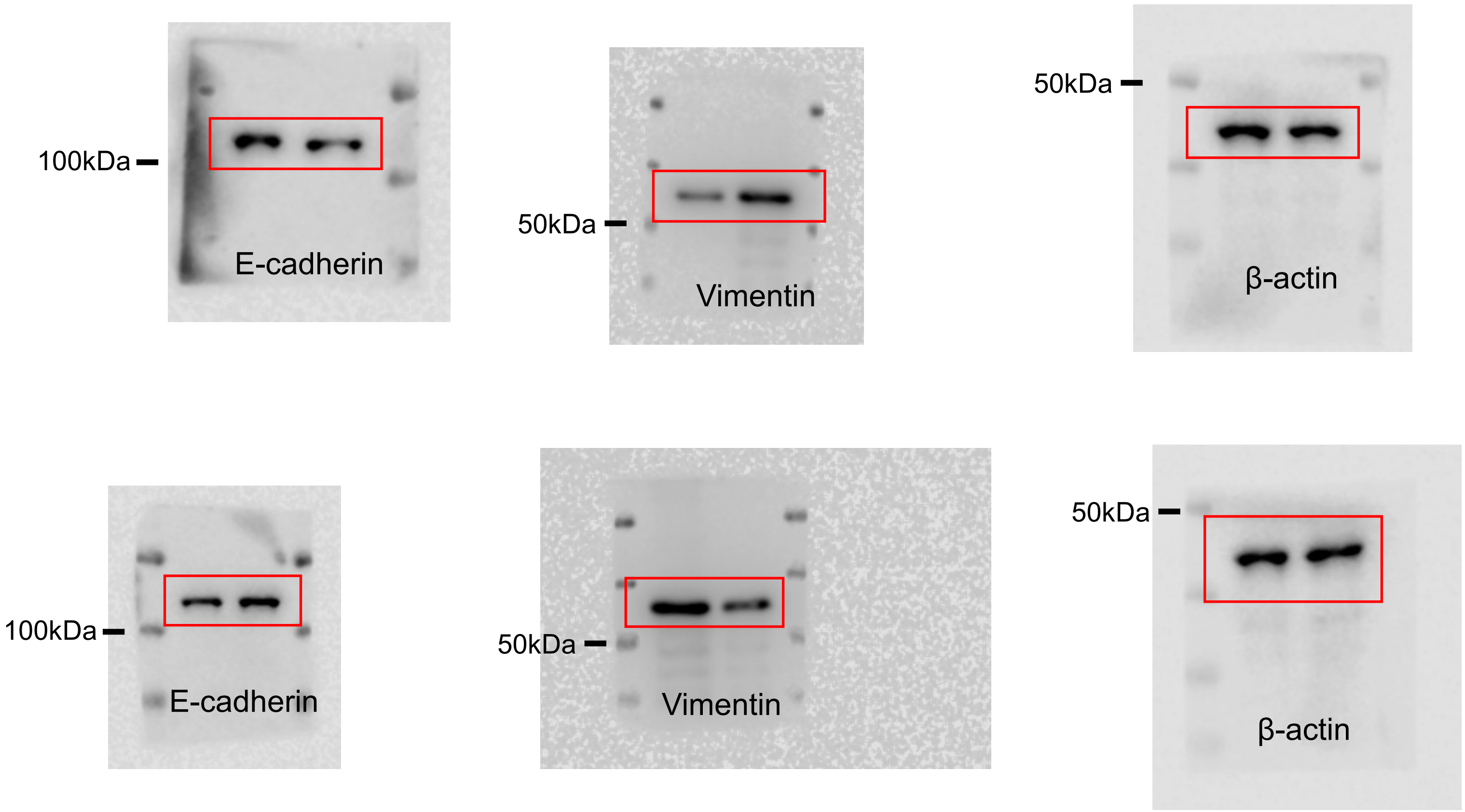

Extended Data Fig. 3N

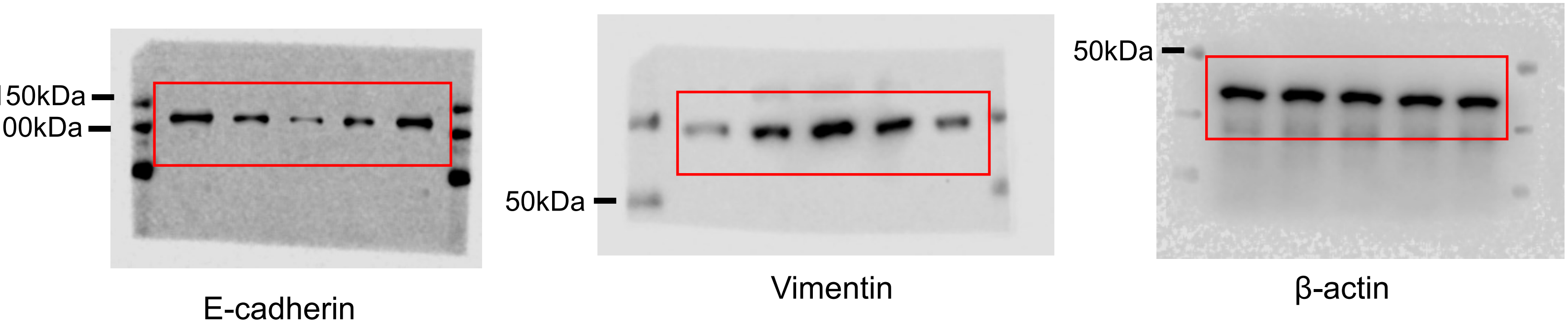

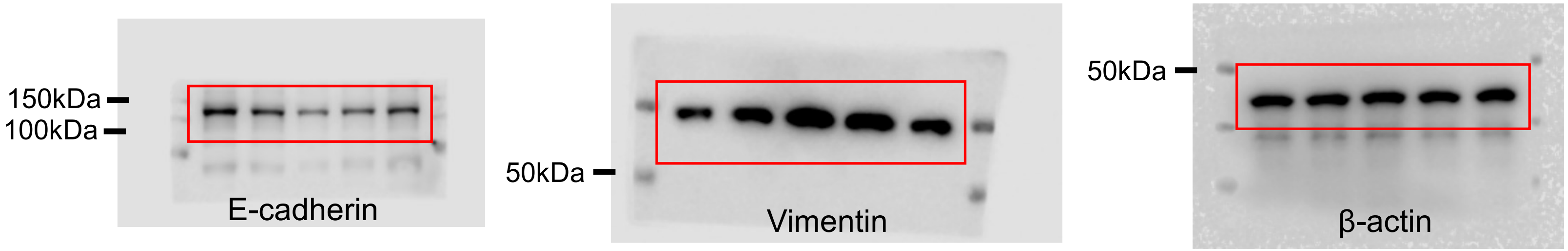

Extended Data Fig.4C

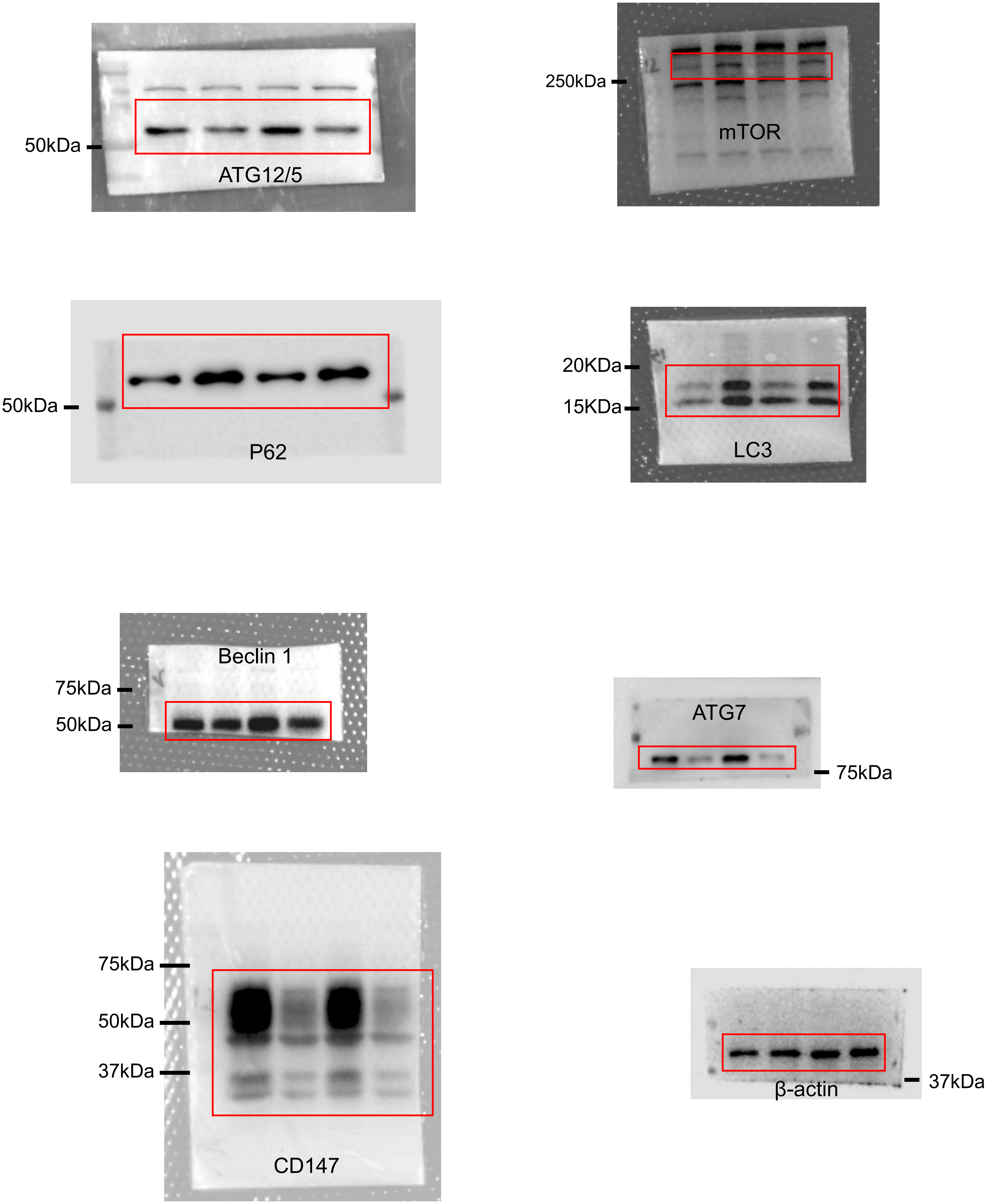

Extended Data Fig.6A

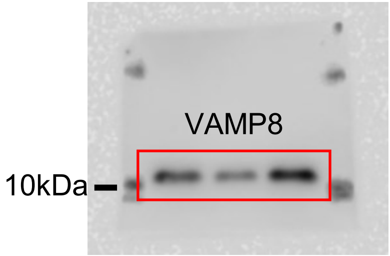

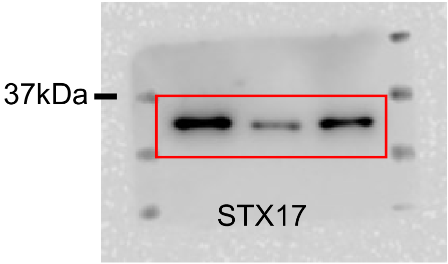

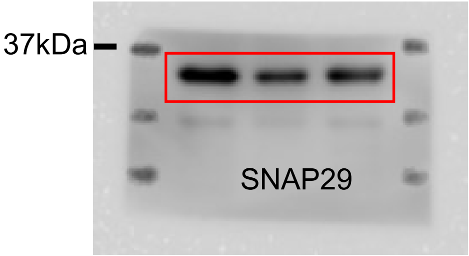

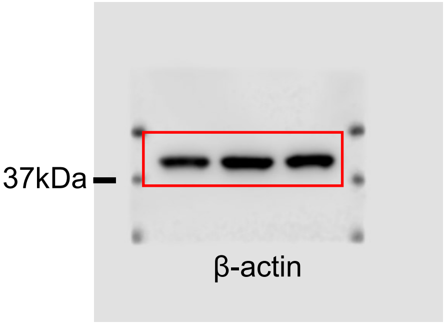

Extended Data Fig.7A

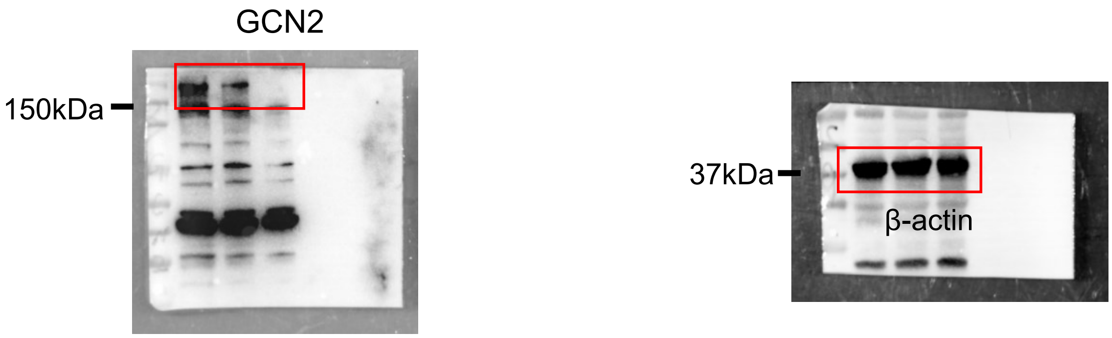

Extended Data Fig.7B

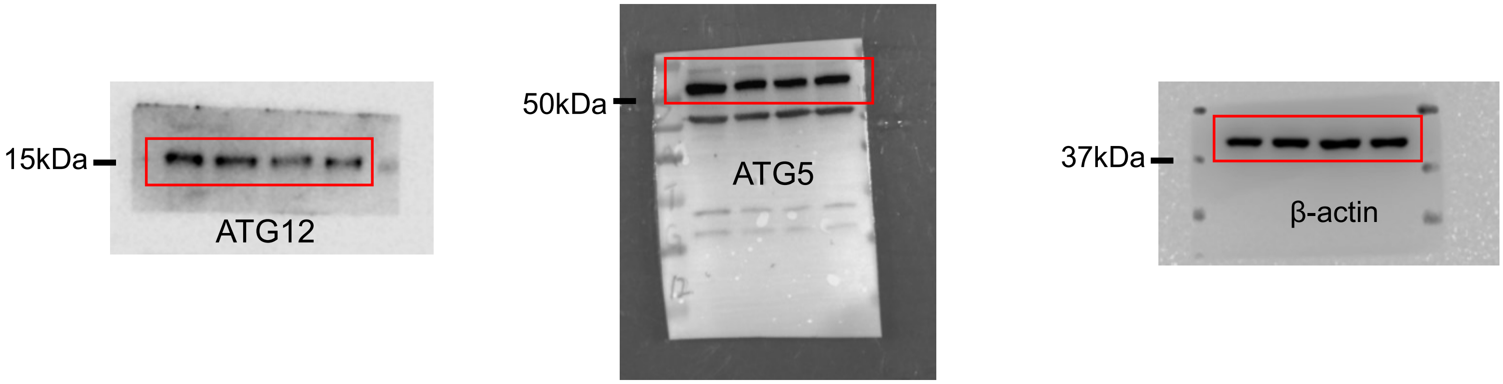

Extended Data Fig.7C

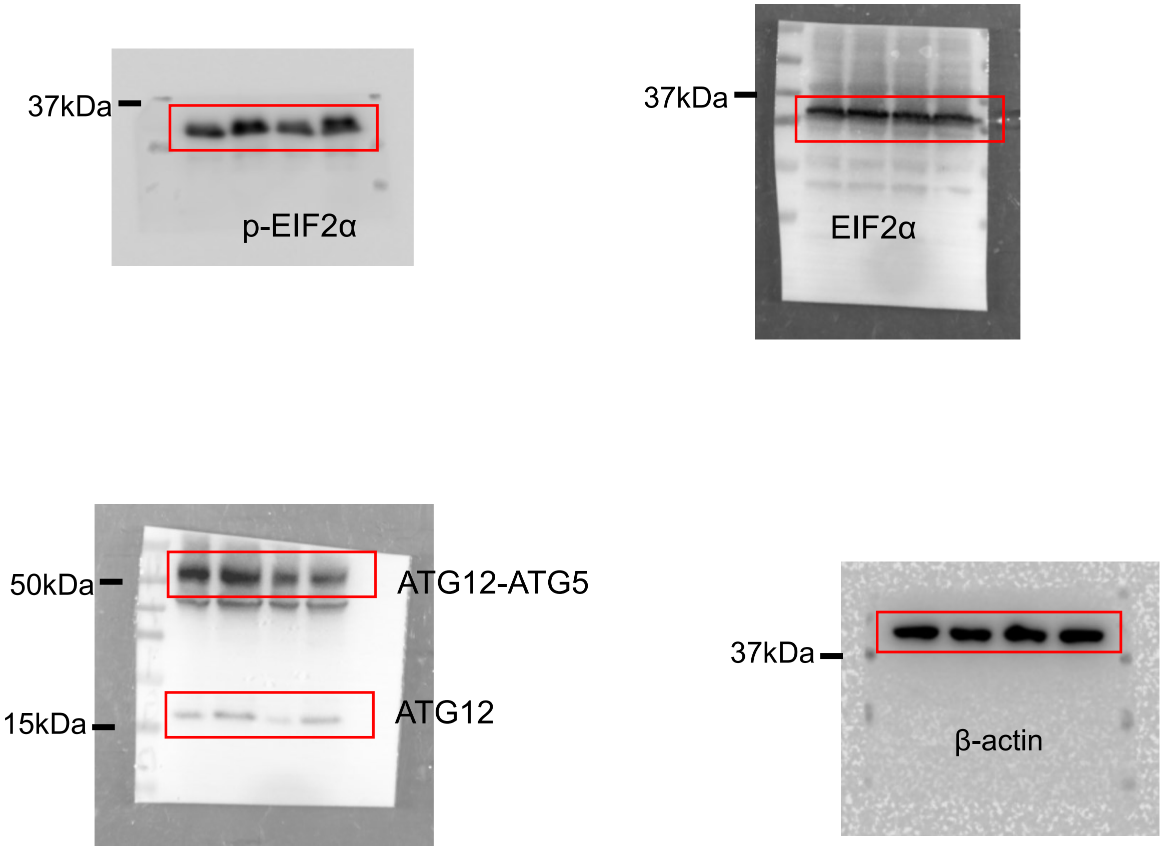

Extended Data Fig. 8B

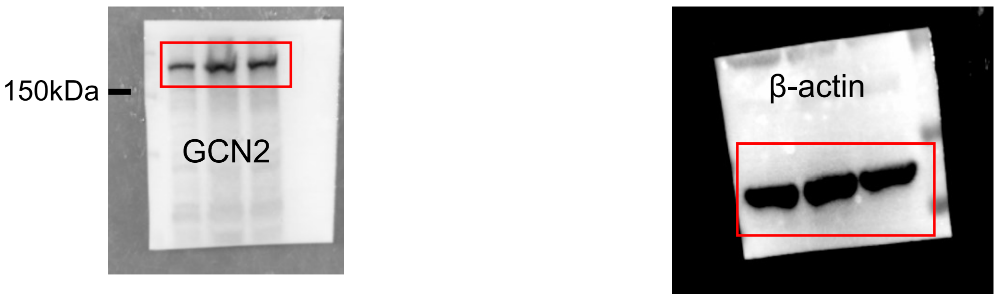

Extended Data Fig. 9A

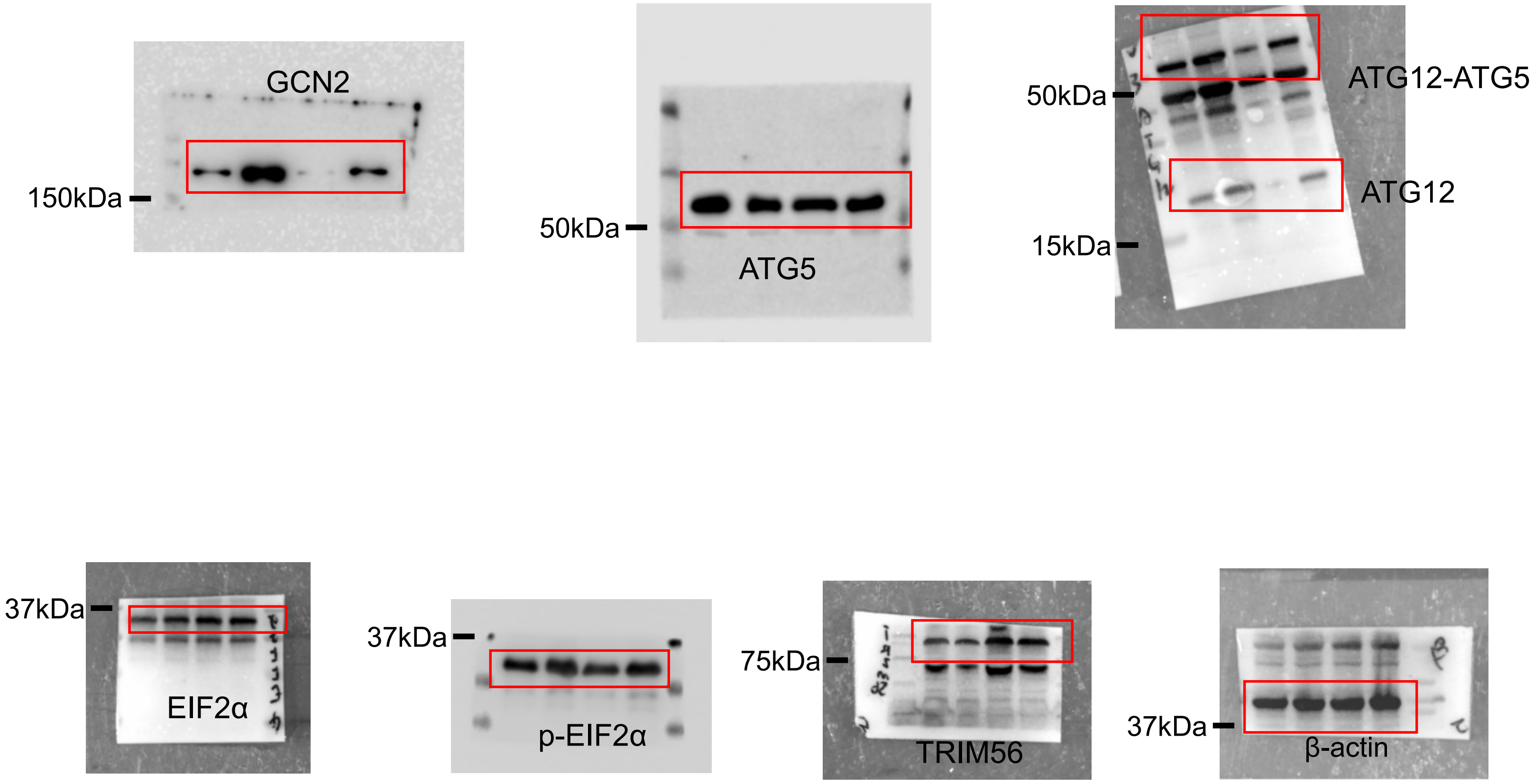

Extended Data Fig. 10D

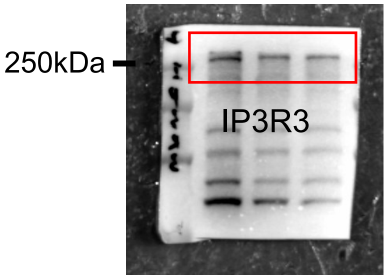

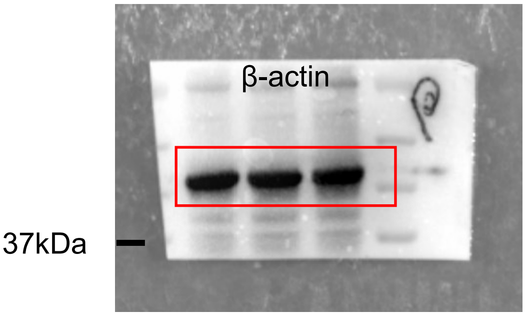

Extended Data Fig. 10I

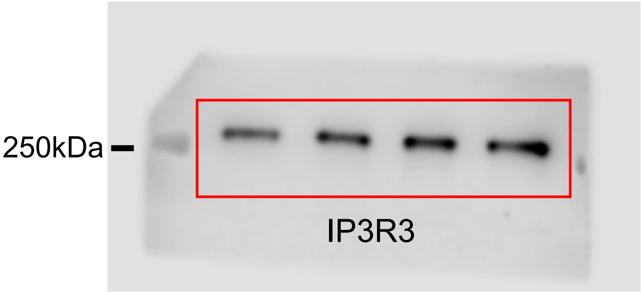

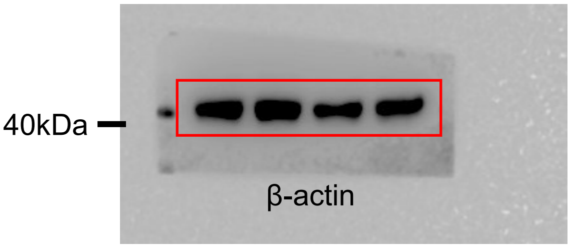

Extended Data Fig. 10J

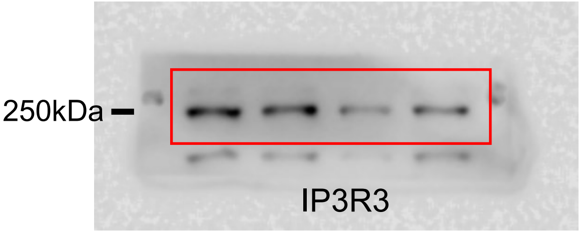

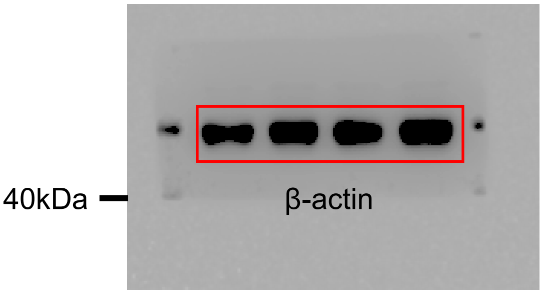

Extended Data Fig. 10K

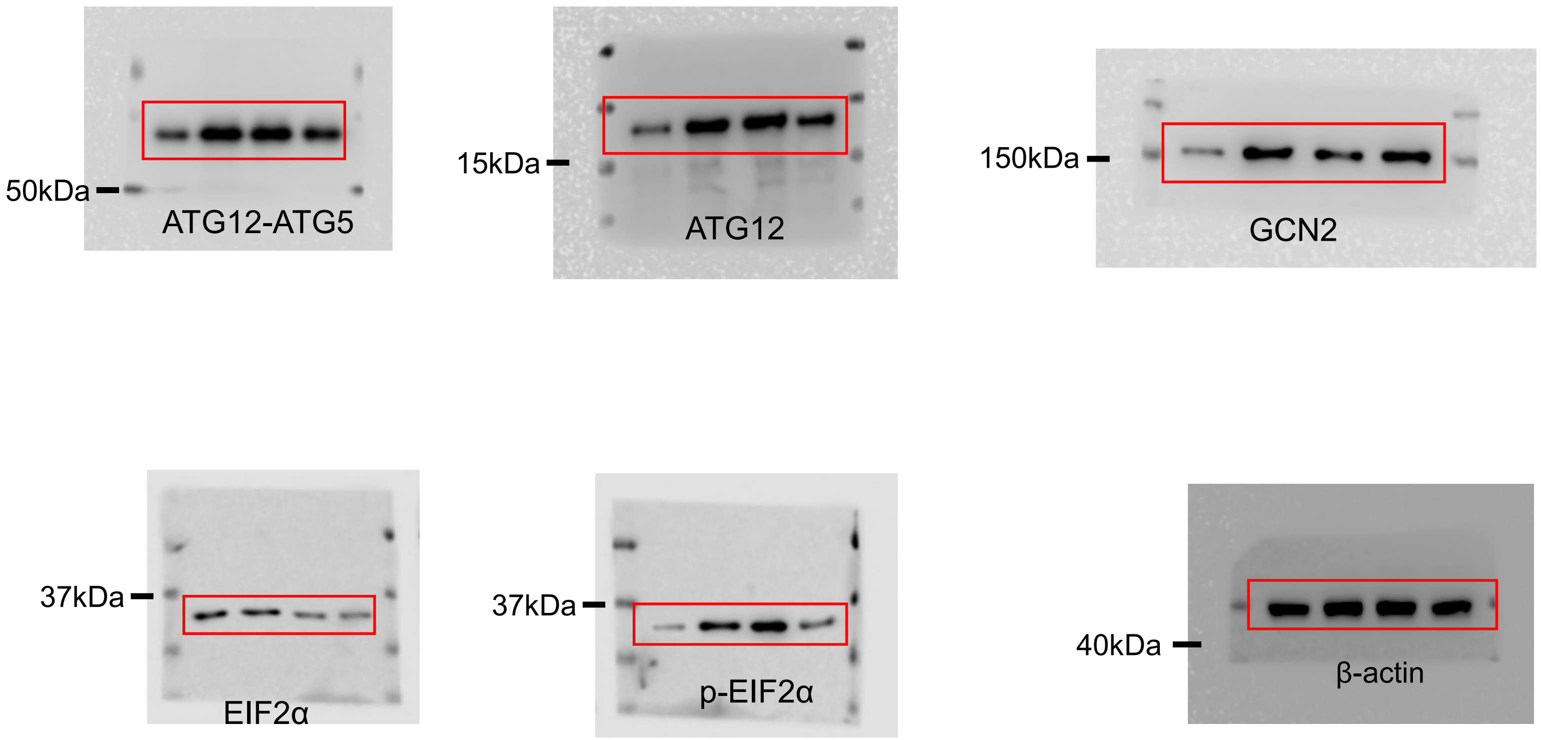

Extended Data Fig. 10L

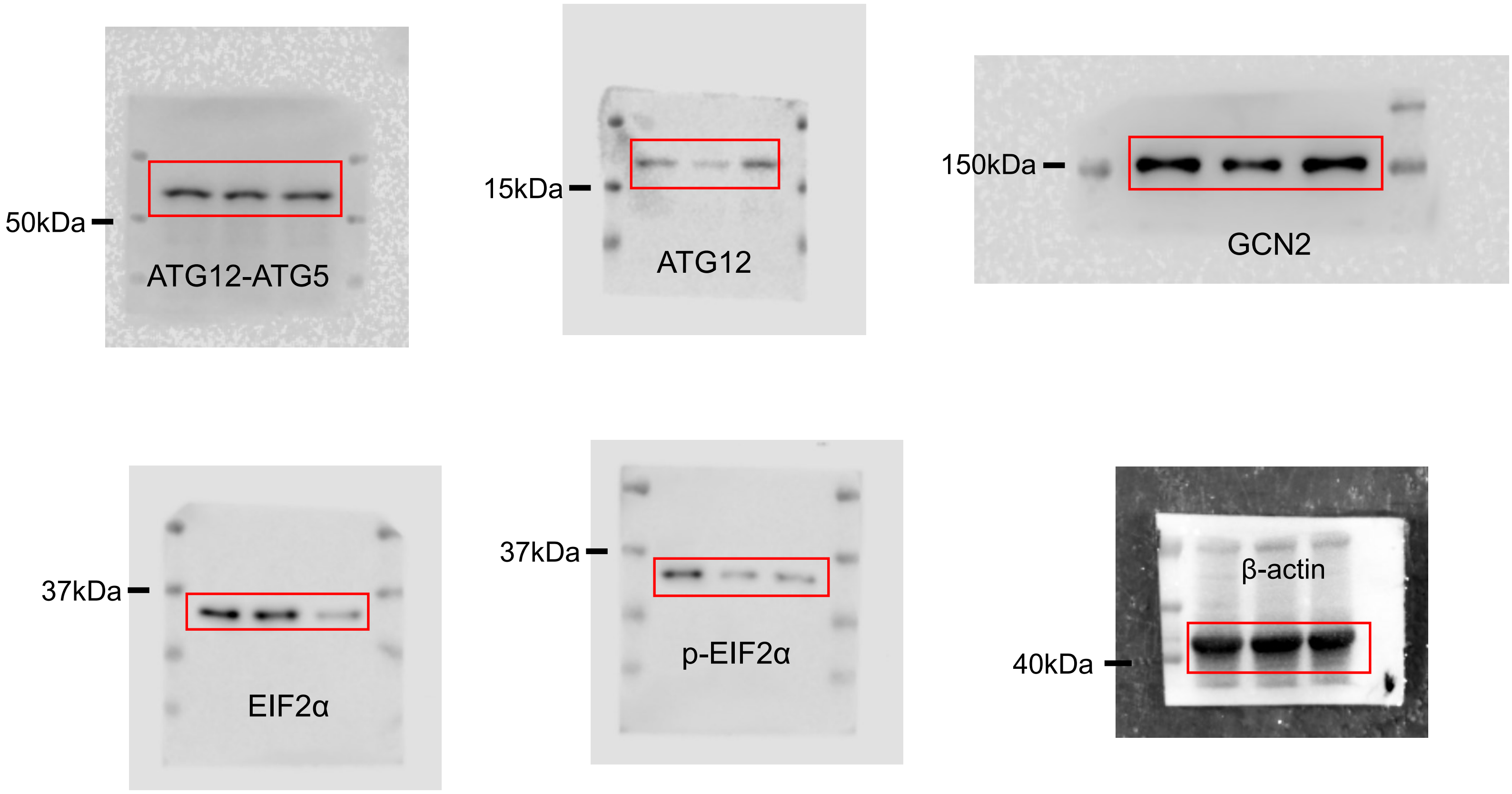

Extended Data Fig. 11A, B

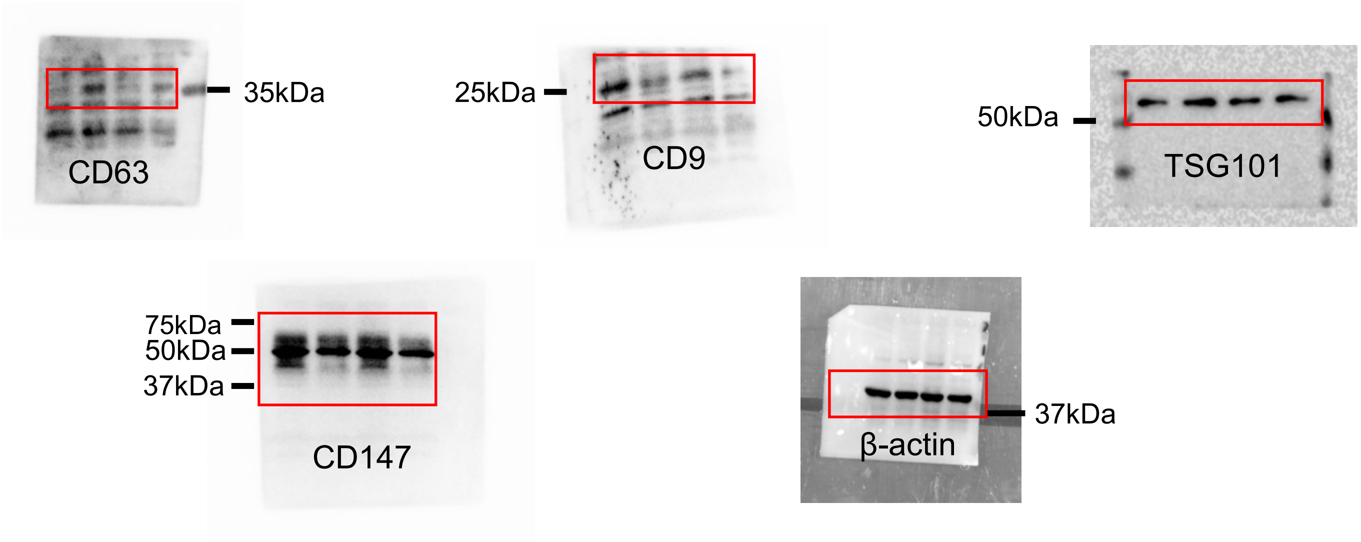

Extended Data Fig. 11F

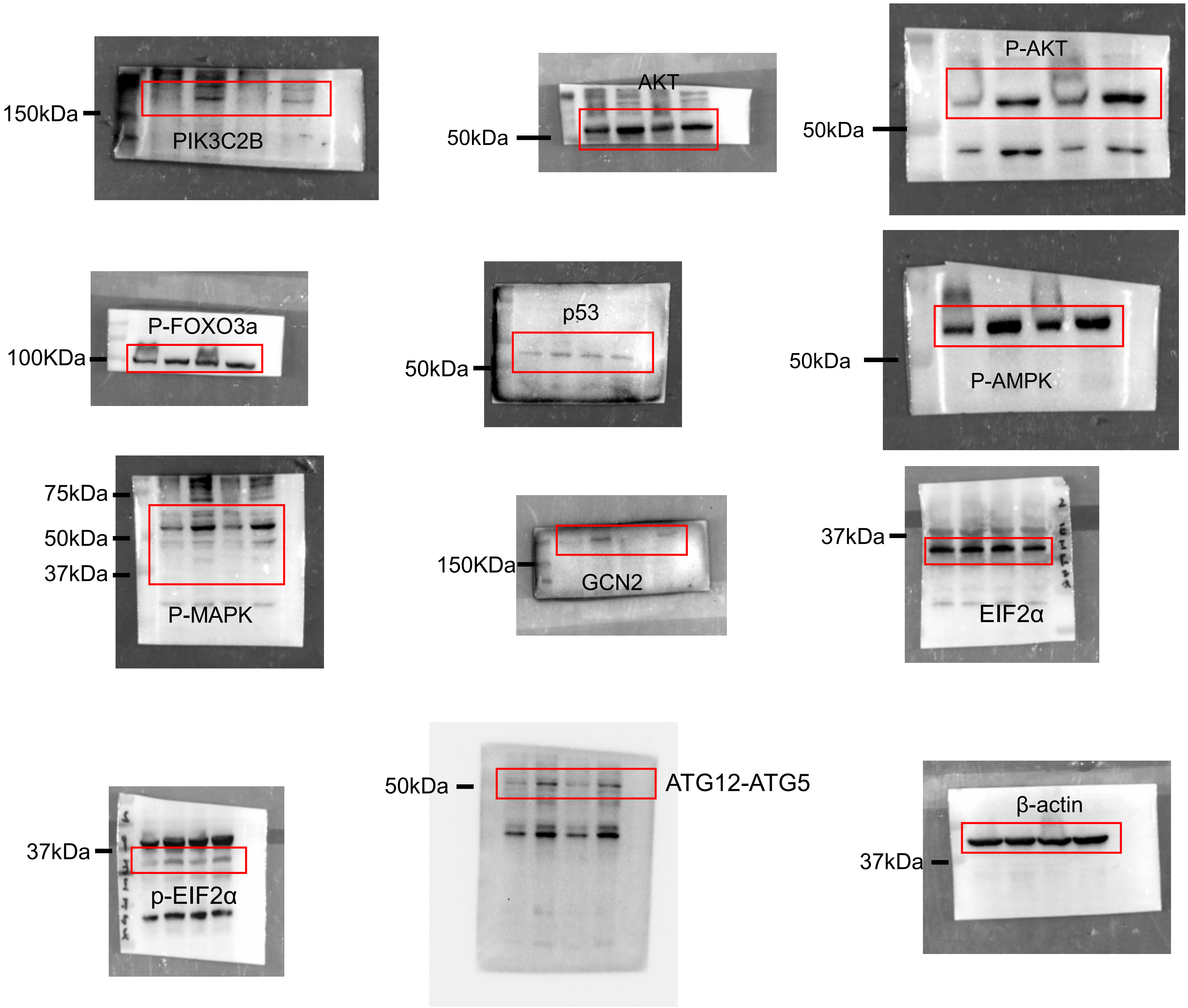

Extended Data Fig. 11G

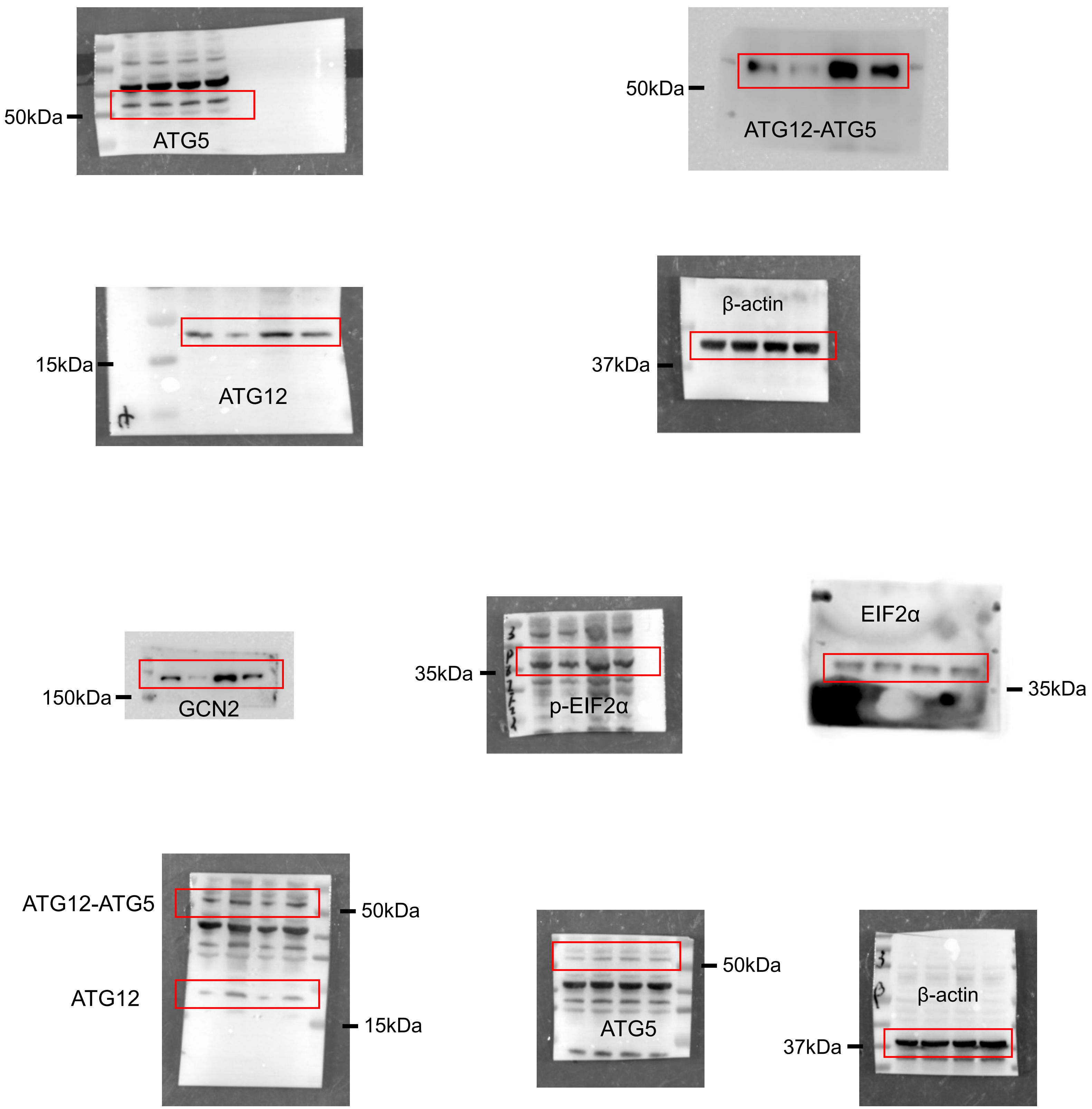

Supplement: Supplementary file 4 — Unprocessed western blots [file 41418_2025_1636_MOESM4_ESM.pdf]
